# Molecular epidemiology and HIV-1 variant evolution in Poland between 2015 and 2019

**DOI:** 10.1038/s41598-021-96125-w

**Published:** 2021-08-16

**Authors:** Karol Serwin, Anna Urbańska, Kaja Scheibe, Magdalena Witak-Jędra, Maria Jankowska, Maria Hlebowicz, Monika Bociąga-Jasik, Anna Kalinowska-Nowak, Martyna Biała, Hubert Ciepłucha, Władysław Łojewski, Anita Olczak, Elżbieta Jabłonowska, Aldona Kowalczuk-Kot, Błażej Rozpłochowski, Adam Witor, Miłosz Parczewski

**Affiliations:** 1 Department of Infectious, Tropical Diseases and Immune Deficiency, Pomeranian Medical University in Szczecin, Arkońska 4, 71-455 Szczecin, Poland; 2 Pomeranian Center for Infectious Diseases and Tuberculosis, Gdańsk, Poland; 3 Pomeranian Hospital, Gdansk, Poland; 4 Department of Family Medicine and Infectious Diseases, University of Warmia and Mazury in Olsztyn, Olsztyn, Poland; 5 Department of Infectious and Tropical Diseases, Jagiellonian University Medical College, Kraków, Poland; 6 Department of Infectious Diseases, Liver Disease and Acquired Immune Deficiencies, Wroclaw Medical University, Wrocław, Poland; 7 Department of Infectious Diseases, Regional Hospital in Zielona Gora, Zielona Góra, Poland; 8 Department of Infectious Diseases and Hepatology, Faculty of Medicine, Nicolaus Copernicus University Ludwik Rydygier Collegium Medicum in Bydgoszcz, Bydgoszcz, Poland; 9 Department of Infectious Diseases and Hepatology, Medical University of Łódź, Łódź, Poland; 10 Department of Infectious Diseases, Medical University of Białystok, Białystok, Poland; 11 Department of Infectious Diseases, Hepatology and Acquired Immunodeficiencies, Karol Marcinkowski University of Medical Sciences, Poznań, Poland; 12 Regional Hospital, Out-Patient’s Clinic for Immune Deficiency, Chorzów, Poland

**Keywords:** Infectious diseases, Phylogeny, Evolutionary biology, Sequencing, Virology

## Abstract

The occurrence of HIV-1 subtypes differs worldwide and within Europe, with non-B variants mainly found across different exposure groups. In this study, we investigated the distribution and temporal trends in HIV-1 subtype variability across Poland between 2015 and 2019. Sequences of the *pol* gene fragment from 2518 individuals were used for the analysis of subtype prevalence. Subtype B was dominant (n = 2163, 85.90%). The proportion of subtype B-infected individuals decreased significantly, from 89.3% in 2015 to 80.3% in 2019. This was related to the increasing number of subtype A infections. In 355 (14.10%) sequences, non-B variants were identified. In 65 (2.58%) samples, recombinant forms (RFs) were noted. Unique recombinant forms (URFs) were found in 30 (1.19%) sequences. Three A/B recombinant clusters were identified of which two were A6/B mosaic viruses not previously described. Non-B clades were significantly more common among females (n = 81, 22.8%, p = 0.001) and heterosexually infected individuals (n = 45, 32.4%, p = 0.0031). The predominance of subtype B is evident, but the variability of HIV-1 in Poland is notable. Almost half of RFs (n = 65, 2.58%) was comprised of URFs (n = 30, 1.19%); thus those forms were common in the analyzed population. Hence, molecular surveillance of identified variants ensures recognition of HIV-1 evolution in Poland.

## Introduction

Human immunodeficiency virus type 1 (HIV-1) has caused a pandemic due to a significant genetic variation produced by high replication rate (viral doubling time is 0.65 days)^1^ and lack of the reverse transcriptase (RT) proofreading activity, which generates a considerable error rate (4.1 ± 1.7) × 10^−3^ per base per cell^2^. Four phylogenetically distinct groups (O, P, N, M) have been identified so far^3^, and the predominant group M (main) has evolved into nine subtypes (A–D, F–H, J, K), of which subtypes A and F have been further subdivided into sub-lineages (A1–A4, A6, and F1, F2)^4^.

Furthermore, inter-subtype recombination events within a single infected individual result in the emergence of novel variants such as circulating recombinant forms (CRFs) and unique recombinant forms (URFs). A specific variant of new circulating viruses is defined when a monophyletic clade of the same inter-subtype structure is found among at least three epidemiologically separated individuals. While URF is observed less frequently with no evidence of onward transmission in the population. To date, based on the LANL-HIV (Los Alamos National Laboratory) database, 104 CFRs have been reported. The most recent, found in China, is composed of the CRF01 and CRF07 types, indicating secondary CRF formation—CRF104_0107^5^.

The most common variant of HIV-1 observed worldwide remains subtype C (46.6% of the infected global population) followed by subtype B (12.1%), and subtype A (10.3%). In western and central Europe, the most ubiquitous are subtypes B (83.3%), C (3.9%), and A (1.9%)^6^. Subtype B vs. A proportion is inverse in eastern Europe (A—52.8% vs. B—17.4%).

From the first recorded case in 1985 until the end of 2019, there were 25,544 people diagnosed with HIV-1 in Poland. Infection prevalence is stable at 0.1% of the population. In recent years, men aged 30–39 years predominate among infected individuals, with the primary route of transmissions being men who have sex with men (MSM)^7^. Around 1200 new cases are recorded annually in the country, with a slow increasing trend. In Poland, thus far, subtype B has been the most prevalent (86.9%) followed by subtype A (5.2%)^8^. There are also non-B monophyletic clusters containing a range of divergent variants and recombinants^9^.

Genetic variability of HIV-1, reflected in subtypes and recombinant forms, impacts the rate of progression, virus tropism, and patterns of drug resistance^10^. Therefore, reconnoitering subtype diversity across populations provides valuable information about virus spread^11^. In this study, we aimed to present molecular surveillance data on HIV variant evolution in the recent years, with the inclusion of the transmission route and the clinical characteristics of non-B clades. For the first time, we include sub-lineages of variant A in the Polish epidemic nomenclature. Moreover, we performed a detailed recombination breakpoints analysis to reveal unique recombinant clusters.

## Results

### Prevalence of HIV-1 subtypes and recombinants

**Figure 1:**
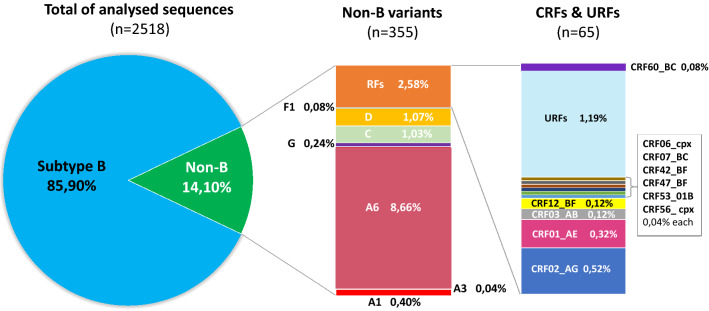
Distribution of HIV-1 variants in Poland between 2015–2019 according to phylogenetic and recombination analyses. RFs—(recombinant forms) comprise of CRF—(circulating recombinant forms) and URFs—(unique recombinant forms).

**Figure 2:**
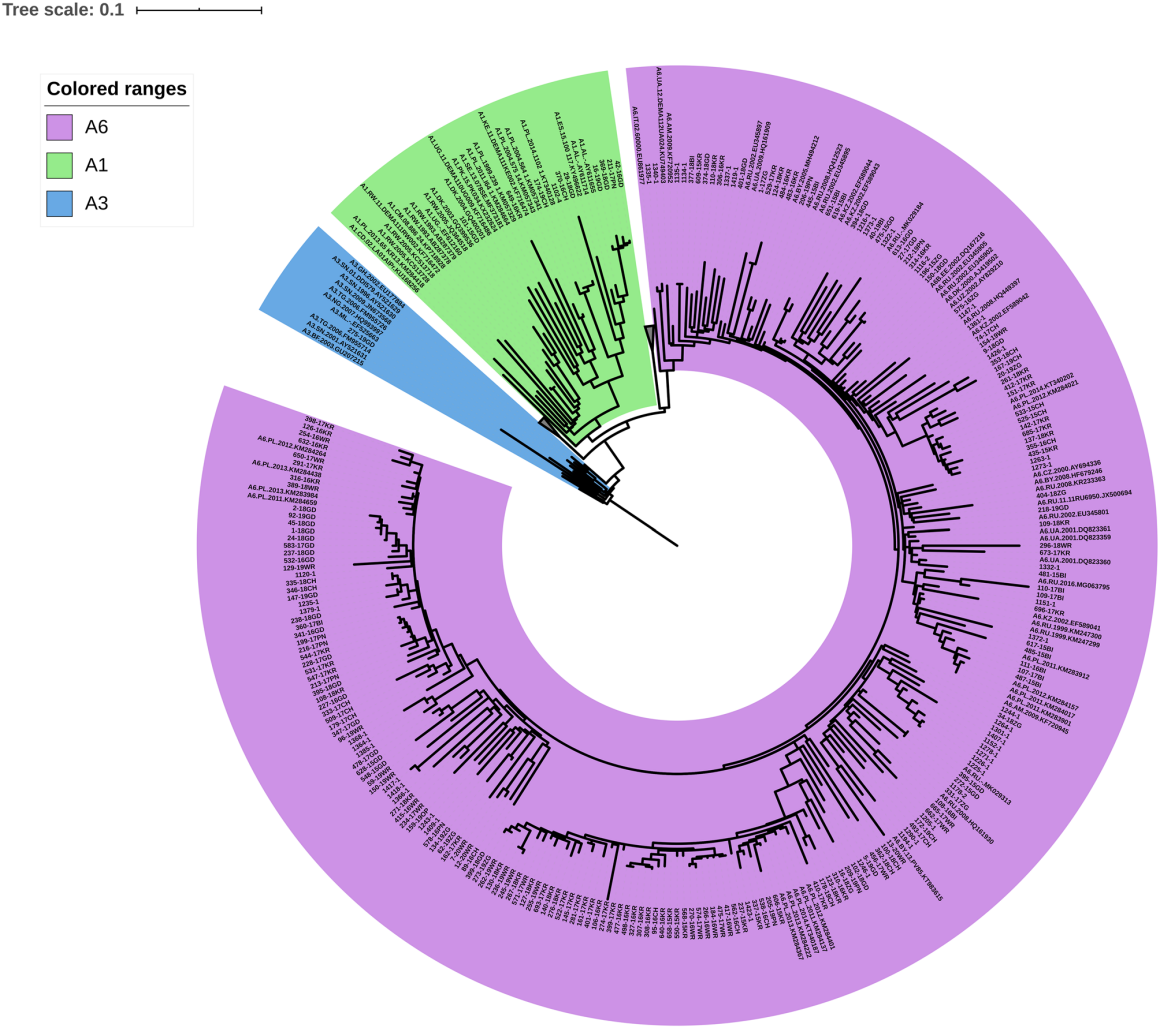
Phylogenetic tree inferred using the maximum likelihood method with HIV references (only Subtype A n = 15, CRF01_AE n = 10, and CRF02_AG n = 10) from HIV sequence compendium 2017, supplemented with HIV-1 sequence database-deposited sequences for subtype A (n = 71) with 229 sequences (of partial 1302 bp HIV-1 *pol* gene) for A-clade that were found in the studied population. Branches containing CRF01_AE and CRF02AG clade have been collapsed. The tree was rooted with group O (accession no KY953205), however, this root was removed from the final figure. The Figure was made using iTol (43).

**Figure 3:**
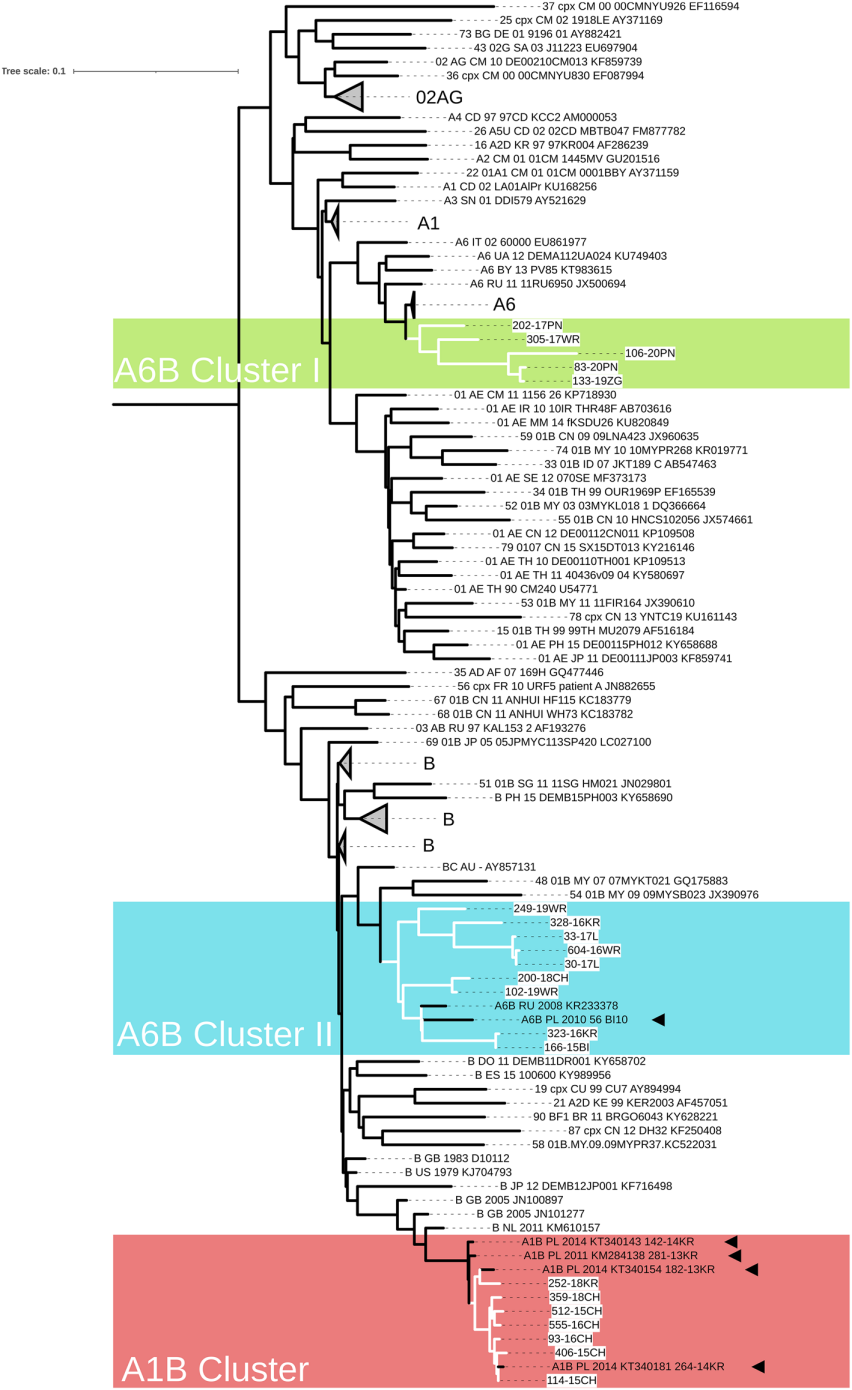
Phylogenetic tree of partial HIV-1 *pol* sequences (1302 bp) inferred using ML method with HIV-1 reference from HIV sequence compendium 2017 (all A and B subtypes sequences and CRFs with at least one fragment of either A or B variant), supplemented with HIV-1 sequence database-deposited sequences for subtype A and B recombinants with 21 unique recombinant forms found in the studied group. White highlights indicate A/B recombinant sequences. The black triangles mark the URF sequences found in our previous publication (from 2008–2014). Branches containing the same HIV-1 clade have been collapsed. The tree was rooted with group O (accession no KY953205), however, this root was removed from the final figure. The Figure was made using iTol (43).

In the study group, subtype B was dominant (n = 2163, 85.90%). In 355 (14.10%) sequences, non-B subtypes (A6, n = 218, 8.66%; D, n = 27, 1.07%; C, n = 26, 1.03%; A1, n = 10, 0.40%; G, n = 6, 0.24%; F1, n = 2, 0.08%; and A3, n = 1, 0,04%) were identified (Fig. 1). As the online subtyping tools did not allow proper identification of subtype A sub-lineages, a tree with inclusion of the reference and regional sub-subtype A sequences was inferred (Fig. 2). Phylogenetic tree indicated that most of the analyzed A6 variants cluster within local reference sequences from Poland, Ukraine, Czech Republic, and Russia. In 65 samples (2.58%), recombinant forms were noted, including: CRF02_AG (n = 13, 0.52%), CRF01_AE (n = 8, 0.32%), for both CRF03_AB and CRF12_BF (n = 3, 0.12%), and CRF60_BC (n = 2, 0.08%). A single case (0.04%) was observed for each of CRF06_cpx, CRF07_BC, CRF42_BC, CRF47_BF, CRF53_01B, and CRF56_cpx. A phylogenetic tree containing all non-B, non-A viruses, with exception of the URF variants is shown in Supplementary Figure S1. URFs were found in thirty (1.19%) sequences, of which 22 (0.87%) were A/B recombinants (Fig. 3). Four of the remaining sequences were B/F1 recombinants and the last four were composed of subtypes CRF_02AG/H/G; A1/D/B; F2/CRF02_AG; CRF11_cpx/CRF02_AG (Supplementary Figure S2).

### Distribution of subtypes over the analyzed regions

**Figure 4:**
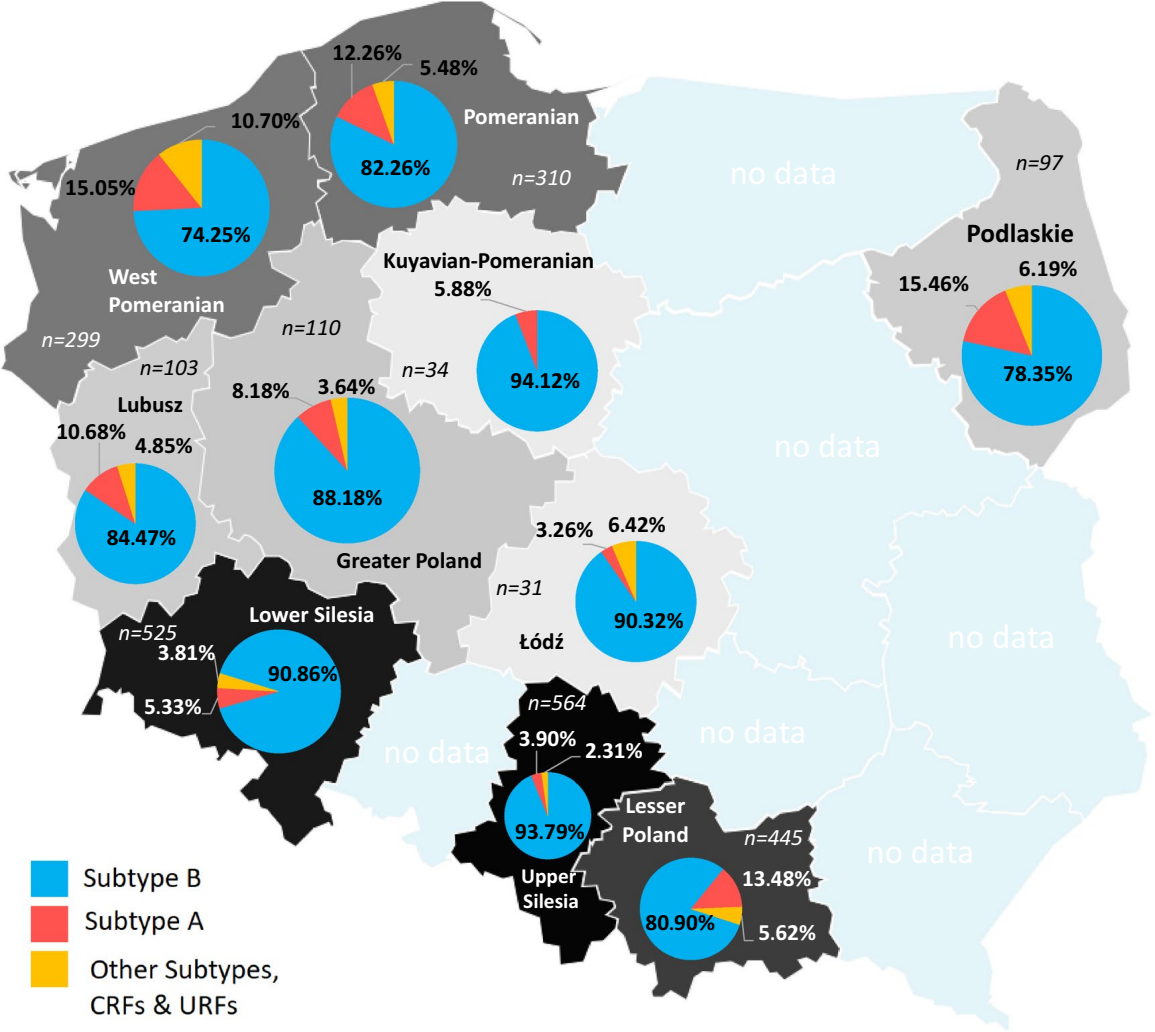
Distribution of subtype B, subtype A and all non-B, and non-A variants across analysed provinces.

The incidence of non-B subtypes between 2015 and 2019, varied from 5.88% in the mid-northern region (Kuyavian-Pomeranian) to 25.75% in the West Pomerania. In six of the ten investigated regions, non-B clades had significantly higher proportions compared to the entire studied sample (Fig. 4 and Supplementary Table 1). In north-western Poland, the increase in non-B variants (OR: 2.42, 95% CI 1.82–3.23, p < 0.001) was mainly due to the increased representation of subtype A (OR: 1.96, 95% CI 1.34–2.78, p < 0.001) and subtype D (OR: 27.9, 95% CI 11.1–69.6, p < 0.001). A similar situation occurred in three provinces, where the lower distribution of subtype B was driven by a significant share of subtype A infections compared to the whole analyzed population. Firstly, Podlaskie with 78.3% for subtype B (OR: 0.58, 0.35–0.95, p < 0.05) and 15.5% for subtype A (OR: 1.88, 95% CI 1.07–3.33, p < 0.05). Secondly, Lesser Poland with 80.9% for subtype B (OR: 0.63, 95% CI 0.48–0.83, p < 0.001), and 13,5% for subtype A (OR: 1.76, 95% CI 1.28–2.40, p < 0.001). Lastly, the increment of subtype A (12.3%) (OR: 1.62, 95% CI 1.12–2.35, p < 0.05) in Pomerania is associated with decreased occurrence of subtype B (82.3%) (OR: 0.73, 95% CI 0.53–0.99, p < 0.05) below the national calculated value. In contrast, in south-western Poland, there were two regions with a notable high prevalence of B clade versus the total studied population. Upper Silesia with only 3.9% subtype A contribution (OR: 0.34, 95% CI 0.22–0.54, p < 0.001) and high share (93.8%) of subtype B infections (OR: 2.96, 95% CI 2.06–4.25, p < 0.001). Moreover, Lower Silesia possessed a large proportion of subtype B (90.9%) (OR: 1.81, 95% CI 1.31–2.49, p < 0.001) contrary to subtype A individuals (5.3%) (OR: 0.50, (95% CI 0.33–0.76), p < 0.001).

### Characterization of the A/B clusters

#### Identified monophyletic clusters

Phylogenetic analysis revealed that the identified A/B variants formed three monophyletic clusters. These three clusters contained sequences obtained from males of Polish citizenship and Caucasian ethnicity but there was no information available on epidemiological relationships between the patients. The A6/B sequence Cluster-I covered three sequences identified in the city of Poznan, and two in the centers of Wroclaw and Zielona Gora. The A6/B Cluster-II contained nine isolates from five distinct provinces (Wroclaw = 3, Lodz = 2, Cracow = 2, Chorzow = 1, and Bialystok = 1). The A1/B Cluster contained six sequences obtained from the patients followed up in the city of Chorzow and one from Cracow. Furthermore, one unclustered A6/B URF sequence (no 258-19WR) was found. A BLAST search analysis indicated two different *pol* regions with high homology to sequences within the A6/B Cluster-II and four to sequences within the A1/B Cluster. The sequences for PR, RT, and IN (consisting of 2168 bp length) were used in the bootscaning analysis for unveiling the recombination profiles of all three AB Clusters.

##### Cluster I

**Figure 5:**
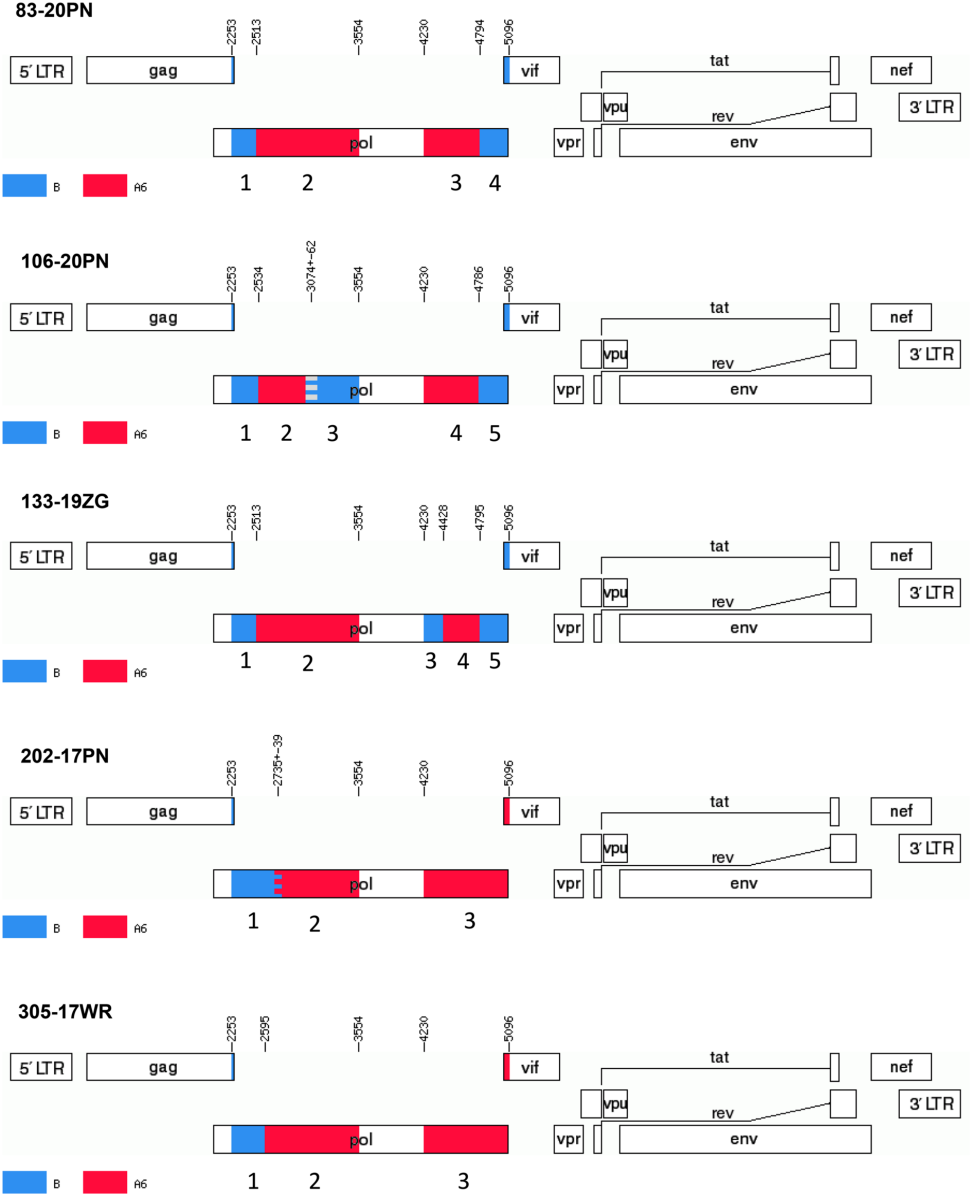
Recombination pattern of the five partial HIV-1 *pol* sequences from the identified A6B Cluster 1. Analysed region include the protease with a fragment of the reverse transcriptase gene (2253–3554 bp) and the integrase gene (4230–5096 bp). Recombination breakpoints (according to HXB2 genome positions) were obtained using Simplot v3.5.1 software and jumping-profile hidden Markov model (jpHMM). The blue color stands for HIV-1 subtype B, red color stands for sub-subtype A6. Component subtype regions are labeled from 1 up to 5 on the genome map, corresponding with numbered on phylogenetic trees in Supplementary Figure S3. The mosaic map was generated using the Recombinant HIV-1 Drawing Tool (https://www.hiv.lanl.gov/content/sequence/DRAW_CRF/recom_mapper.html).

Phylogenetic and bootscanning analyses of fragments from the A6/B Cluster-I showed a complex breakpoint profile (Fig. 5; Supplementary Figure S3 and Figure S4). In two sequences (202-17PN and 305-17WR) one split region was observed (subtype B to A6 breaks occurred between 2595 to 2774 bp positions of HXB2 genome) in the PR_RT region, but the entire IN sequence was identified as of the A6 lineage. In one sequence (83-20PN) two breakpoints were observed: the first one in the PR_RT region (subtype B to A6 ranged from 2498 to 2521 bp) and the second in IN sequence (subtype A6 to B break between 4788–4800 bp). In the last two sequences five subregions were identified corresponding to four break events. The following genome fragments and subtypes were identified: for the 106-20PN isolate, subtype B included the fragments of 2253–2533, 3012–3554, and 4786–5096 bp whereas subtype A6 included the fragments 2534–3011 and 4230–4785 bp; for the 133-19ZG isolate, subtype B included the fragments 2253–2512, 4230–4427, and 4795–5096 bp whereas subtype A6 included the fragments 2513–3554 and 4428–4794 bp.

##### Cluster II

**Figure 6:**
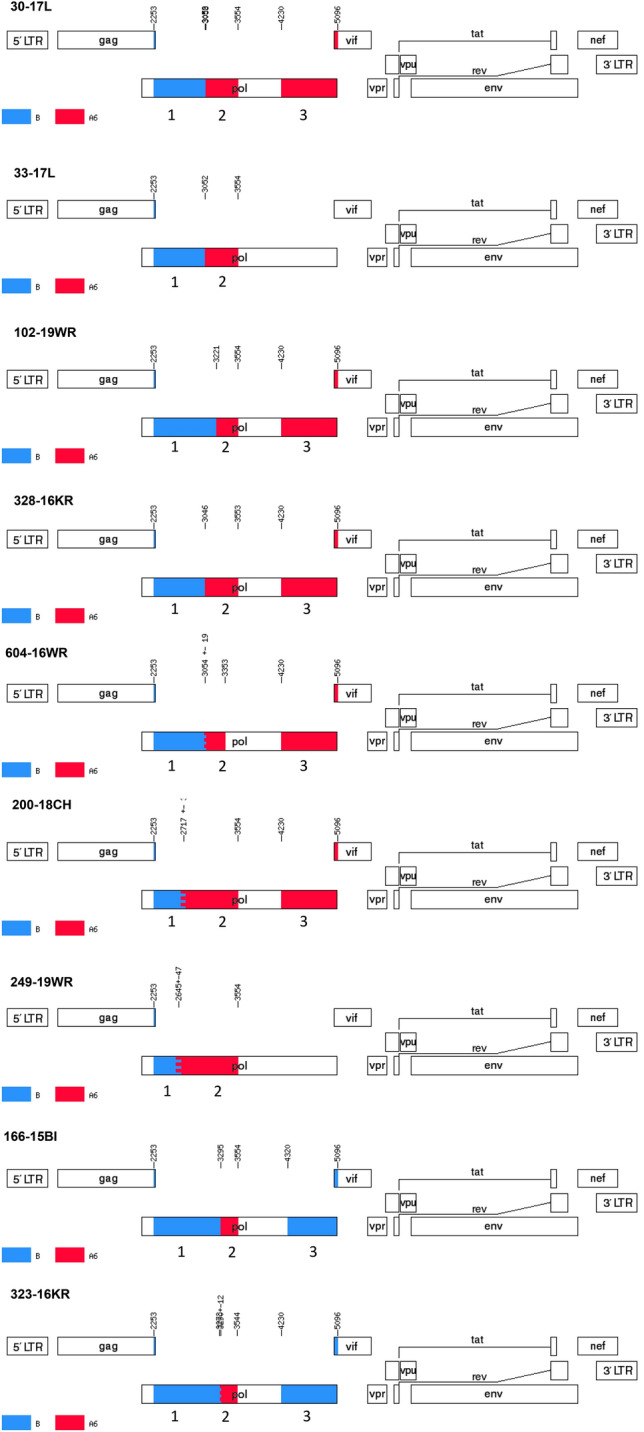
Recombination pattern of the nine partial HIV-1 *pol* sequences from the identified A6B Cluster II. Analysed region include the protease with a fragment of the reverse transcriptase gene (2253–3554 bp) and the integrase gene (4230–5096 bp). In two cases (33-17L, 249-19WR) IN gene was not sequenced. Recombination breakpoints (according to HXB2 genome positions) were obtained using Simplot v3.5.1 software and jumping-profile hidden Markov model (jpHMM). The blue color stands for HIV-1 subtype B, red color stands for sub-subtype A6. Component subtype regions are labeled from 1 up to 3 on the genome map, corresponding with numbered on phylogenetic trees in Supplementary Figure S5. The mosaic map was generated using the Recombinant HIV-1 Drawing Tool (https://www.hiv.lanl.gov/content/sequence/DRAW_CRF/recom_mapper.html).

There were no breakpoints within the IN region among the analyzed HIV-1 sequences that formed the A6/B Cluster-II (Fig. 6; Supplementary Figure S5 and Fig. S6). Two sequences (166-15BI and 323-16KR) had a single breakpoint in PR_RT (subtype B to A6 over 3288–3303 bp) and were of subtype B for the IN region. In these cases, the breaks must have occurred in a 677 bp fragment of the *pol* gene that we had not sequenced. In the remaining isolates (249-19WR, 328-16KR, 33-17L, 604-16WR, 30-17L, 200-18CH, and 102-19WR) one break occurred in the PR_RT sequence (subtype B to A6 break from 2598 to 3221 bp). The IN region for five of these variants (249-19WR, 328-16KR, 33-17L, 604-16WR) were of the A6 sublineage, in two cases (249-19WR and 33-17L) this fragment was not sequenced.

##### Cluster III

**Figure 7:**
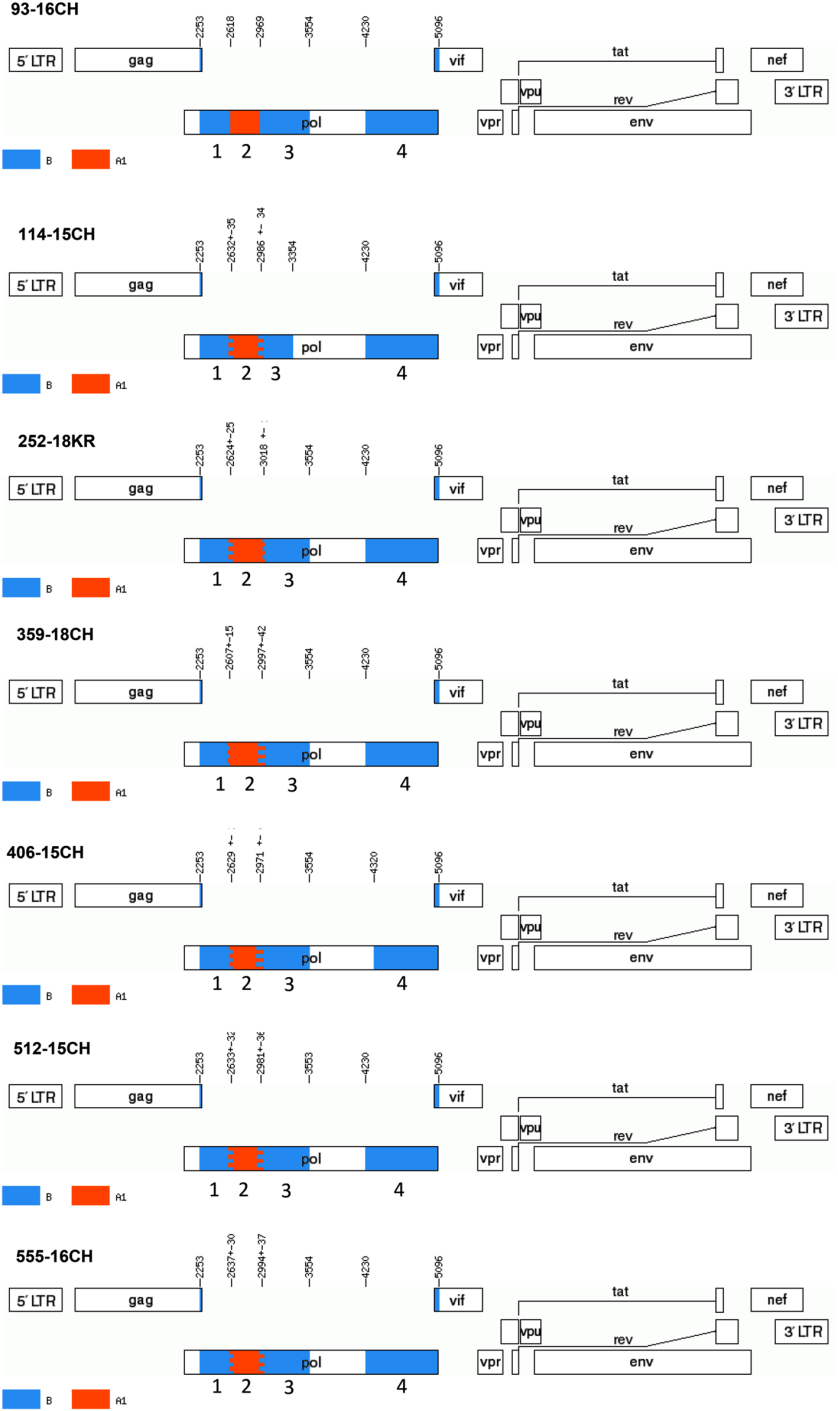
Recombination pattern of the seven partial HIV-1 *pol* sequences from the identified A1B Cluster. Analysed region include the protease with a fragment of the reverse transcriptase gene (2253–3554 bp) and the integrase gene (4230–5096 bp). Recombination breakpoints (according to HXB2 genome positions) were obtained using Simplot v3.5.1 software and jumping-profile hidden Markov model (jpHMM). The blue color stands for HIV-1 subtype B and red color stands for sub-subtype A1. Component subtype regions are labeled from 1 up to 4 on the genome map, corresponding with numbered on phylogenetic trees in Supplementary Figure S7. The mosaic map was generated using the Recombinant HIV-1 Drawing Tool (https://www.hiv.lanl.gov/content/sequence/DRAW_CRF/recom_mapper.html).

The A1B Cluster comprises seven newly described isolates (252-18KR, 359-18CH, 512-15CH, 555-16CH, 93-16CH, 406-15CH, and 114-15CH) that had a very similar pattern of recombination. All IN sequences were subtype B. Within the PR_RT sequences, a short (351 to 415 bp long) insertion of the A1 fragment was noted (Fig. 7; Supplementary Figure S7 and Fig. S8).

### Clinical characteristics of the identified HIV-1 variants

Non-B subtypes were notably more common among females (n = 81, 22.8% for non-B variants versus n = 291, 13.5% for subtype B; p = 0.001). Specifically, a higher proportion of women, compared to subtype B was observed for the A (n = 45, 19.7%; p = 0.01), D (n = 18, 66.7%; p = 0.001), and G (n = 3, 50%; p = 0.036) subtypes. Non-B variants were also more frequent among heterosexually infected (HET) individuals (n = 45, 32.4%) compared to MSM (n = 88, 63.3%; p = 0.0031) and injection drug users (IDU) (n = 6, 4.3%; p < 0.0031), especially for subtype D (n = 4, 66.7%; p = 0.001) and CRF02_AG (n = 2, 100%; p = 0.033) (detailed in Table 1).

**Table 1:** Group characteristics across the analysed subtypes and differences compared to the subtype B.

	Subtype A	p	Subtype B	p	Subtype C	p	Subtype D	p	Subtype F1	p	Subtype G	p	CRF01_AE	p	CRF02_AG	p	Other CRFs (n = 14) and URFs (n = 30)	p	Total non-B	p
	n = 229		n = 2163		n = 26		n = 27		n = 2		n = 6		n = 8		n = 13				n = 355	
**Male, n (%)**	184 (80.3)	0.01 *	1872 (86.5)	ref	20 (76.9)	0.15	9 (33.3)	0.001 *	2 (100)	1	3 (50.0)	0.036 *	6 (75.0)	0.30	11 (84.6)	0.69	39 (88.6)	0.69	274 (77.2)	0.001 *
**Female, n (%)**	45 (19.7)		291 (13.5)		6 (23.1)		18 (66.7)		0		3 (50.0)		2 (25.0)		2 (15.4)		5 (11.4)		81 (22.8)	
***HIV infection stage at genotyping, n (%)(a)***																				
• non-AIDS	70 (84.3)	0.38	473 (80,3)	ref	5 (83.3)	0.85	2 (50.0)	0.13	1 (50.0)	0.28	2 (100)	0.48	3 (75.0)	0.79	3 (100)	0.39	6 (50.0)	0.0098 *	92 (79.3)	0.81
• AIDS	13 (15.7)		116 (19,7)		1 (16.7)		2 (50.0)		1 (50.0)		0		1 (25.0)		0		6 (50.0)		24 (20.7)	
***Dominant transmission rout, n (%)***																				
• Intravenous drug use	4 (3,9)	0.26	37 (5.4)	ref	0	0.40	2 (33.3)	0.001 *	0	0.71	0	0.71	0	0.64	0	0.033 *	0	0.28	6 (4,3)	0.0031 *
• Men having sex with men	71 (69,6)		517 (76)		4 (57.1)		0		1 (50.0)		1 (50.0)		3 (60.0)		0		8 (61.5)		88 (63,3)	
• Heterosexual	27 (26,5)		125 (18,4)		3 (42.9)		4 (66.7)		1 (50.0)		1 (50.0)		2 (40.0)		2 (100)		5 (38.5)		45 (32,4)	
• Vertical	0		1 (0.2)		0		0		0		0		0		0		0		0	
***Lymphocyte CD4* + *T cell*** ***counts at baseline,*** ***median***	457	0.006 *	373	ref	581	0.073	301	0.76	308	0.71	399	0.91	651	0.025 *	387	0.77	176	0.10	454	0.014 *
(IQR)	(291—630)		(176—572)		(471—609)		(139—552)		(230—387)		(271—526)		(570—842)		(280—446)		(28—484)		(244—629)	
***Nadir lymphocyte CD4* + *T cell counts*, median**	451	0.015 *	358	ref	562	0.18	301	0.89	308	0.77	80	0.081	651	0.015 *	387	0.42	140	0.26	444	0.022 *
(IQR)	(282—579)		(174—541)		(423—606)		(301—426)		(230—387)		(60—99)		(570—842)		(280—446)		(42—490)		(249—586)	
***HIV viral load at baseline, mean log copies/ml***	4.7	0.72	4.6	ref	4.4	0.44	4.3	0.36	5.8	0.055	4.2	0.19	4.0	0.096	4.0	0.28	5.5	0.001 *	4.7	0.69
(SD)	(3.8—5.6)		(3.7—5.5)		(3.2—5.6)		(3.1—5.5)		(5.5—6.1)		(3.7—4.7)		(3.1—4.9)		(2.7—5.3)		(4.5—6.5)		(3.6—5.8)	
***Age at diagnosis, median years***	34	0.12	35	ref	37	0.18	40	0.001 *	40	0.92	39	0.19	39	0.21	34	0.88	34	0.24	34	0.21
(IQR)	(29—40)		(29—42)		(31—51)		(39—58)		(32—48)		(35—46)		(34—47)		(30—39)		(29—43)		(30—41)	

(a) HIV infection stage available for 705 patients, transmission route for 819 patients, baseline lymphocyte CD4 + T cell count for 846 patients, nadir lymphocyte CD4 + T cell counts for 791 patients, HIV viral load for 1392 patients and age at diagnosis for 2517 patients.

Overall, cases infected with non-B clades presented with significantly higher CD4 + T-lymphocyte counts at care entry (median: 454 cells/μl, IQR: 244–629) compared to B lineage (median: 373 cells/μl, IQR: 176–572). Similarly, values of *nadir* lymphocyte CD4 + counts were higher for non-B variants [median: 444 cells/μl, IQR: 249–586] vs. subtype B [median: 358 cells/μl, (IQR: 174–541]. These differences were driven mainly by subtype A [median lymphocyte CD4 + count: 457 cells/μl, IQR: 291–630; p = 0.006; median *nadir* lymphocyte CD4 + counts: 451 cells/μl, IQR: 282–579, p = 0.015] as well as CRF01_AE [median lymphocyte CD4 + count: 651 cells/μl, IQR: 570–842, p = 0.025; median *nadir* lymphocyte CD4 + counts: 651 cells/μl, IQR: 570–842, p = 0.015]. Lastly, individuals infected with subtype D were older (median: 40 years, IQR: 39–58) at the time of diagnosis than subtype B cases (median: 35 years, IQR: 29–42). HIV-1 virial load at diagnosis among patients with rare CRFs & URFs variants was higher (mean: 5.5 log copies/ml, IQR: 4.5–6.5) than among subtype B infected cases (median: 4.6 log copies/ml, IQR: 3.7–5.5).

Multivariate logistic regression analysis of the whole dataset with inclusion of gender, transmission route, viral load at diagnosis, CD4 + T cell counts at baseline, *nadir* CD4 + T cell counts, age at diagnosis, and HIV infection stage, indicated that infection with HIV-1 non-B clades was independently associated with female gender (OR: 1.69, 95% CI 1.22–2.34, p = 0.002), higher age at diagnosis (OR: 1.03, 95% CI 1.002–1.052, p = 0.032) and HIV-1 viral load at diagnosis (OR: 1.35, 95% CI 1.05–1.74, p = 0.02).

### Temporal trends for subtype frequency

**Figure 8:**
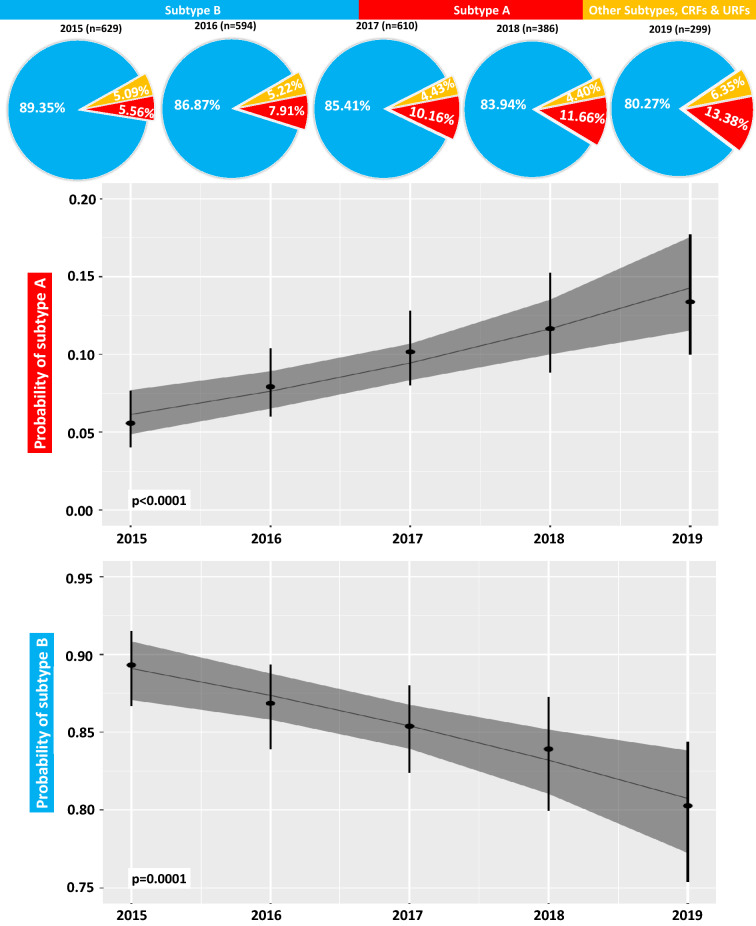
Logistic regression estimates for time trends between 2015–2019 for subtype B and subtype A, together with pie charts of subtype prevalence each year. Dots indicate the percentage per year and vertical bars 95% confidence intervals for the percentages with a vertically presented logistic regression time trend line with dark-grey shaded 95% confidence intervals for the regression estimate. Only limited numbers of subtypes other than B and A each year were found; these non-B, non-A subtypes were grouped as "other” on the pie charts.

During the observation timeline, annual changes in the proportion of subtype B individuals decreased significantly, from 89.3% in 2015 to 80.3% in 2019 (OR: 0.85, 95% CI 0.78–0.92, p = 0.0001) (Table 2, Fig. 8). This was related to the increasing number of subtype A infections (from 5.6% in 2015 to 13.4% in 2019 (OR: 1.26, 95% CI 1.14–1.40, p < 0,0001 ). Interestingly, subtype D frequency has dropped from 1.9 to 0.3% (OR: 0.63, 95% CI 0.43–0.87, p = 0.008) in the analyzed years.

**Table 2:** Time trends for non-B subtypes across transmission category, gender, clinical category of HIV infection and lymphocyte CD4 count.

Variable	Total	Non-B variants (%)	Non-B variant frequency in the sampling year n (%)					Odds ratio	95% confidence interval	p value for time trend	Slope
			**2015**	**2016**	**2017**	**2018**	**2019**				
Total non-B variants	2163	355 (14.10)	67 (10.67)	78 (13.1)	89 (14.6)	62 (16.1)	59 (19.7)	1.18	1.09–1.29	0.0001 *	3.31%
**Transmission category**											
Heterosexual	170	45 (26.5)	11 (17.5)	16 (30.8)	14 (34.1)	3 (25.0)	1 (50.0)	1.34	0.96–1.88	0.089	5.85%
Injection drug use	43	6 (13.9)	4 (20.0)	0	2 (15.4)	0	0	0.72	0.23–1.77	0.496	- 1.78%
Men who have sex with men	605	88 (14.5)	13 (8.6)	26 (12.8)	35 (16.9)	13 (37.1)	1 (12.5)	1.54	1.21–1.96	0.0005 *	8.51%
**Gender**											
Female	372	81 (21.8)	21 (21.2)	15 (18.1)	18 (20.5)	12 (21.4)	15 (32.6)	1.13	0.94–1.35	0.193	2.44%
Male	2146	274 (12.8)	46 (8.7)	63 (12.3)	71 (13.6)	50 (15.2)	44 (17.4)	1.20	1.09–1.32	0.0002 *	3.65%
**Age at diagnosis**											
≤ 30 years	796	104 (13.1)	21 (10.3)	20 (10.5)	29 (13.9)	17 (16.0)	17 (22.7)	1.24	1.06–1.46	0.0065 *	4.30%
31–40 years	996	149 (15.4)	27 (11.3)	38 (15.1)	36 (15.5)	23 (14.4)	25 (22.3)	1.16	1.02–1.33	0.025 *	2.97%
41–50 years	469	55 (11.7)	9 (7.7)	13 (13.7)	10 (9.3%)	11 (13.9)	12 (17.1)	1.20	0.98–1.47	0.080	3.65%
> 51 years	257	47 (18.3)	10 (16.1)	7 (12.3)	14 (23.0)	11 (26.8)	5 (13.8)	1.09	0.87–1.38	0.44	1.72%
**Clinical category at diagnosis**											
Asymptomatic	565	92 (16.2)	20 (12.1)	31 (16.8)	31 (16.8)	9 (29.0)	1 (100)	1.33	1.04–1.70	0.024 *	5.70%
AIDS	140	24 (17.1)	3 (6.5)	7 (16.7)	12 (26.7)	2 (28.6)	0	1.93	1.18–3.29	0.0083 *	13.15%
**Lymphocyte CD4 + T cell counts at baseline**											
> 200 cells/ml	623	107 (17.2)	22 (12.5)	38 (18.2)	33 (17.2)	14 (30.4)	0	1.30	1.04–1.63	0.021 *	5.25%
< 200 cells/ml	223	29 (13.0)	6 (7.7)	8 (11.1)	14 (24.1)	1 (7.1)	0	1.44	0.96–2.16	0.076	7.29%

**Figure 9:**
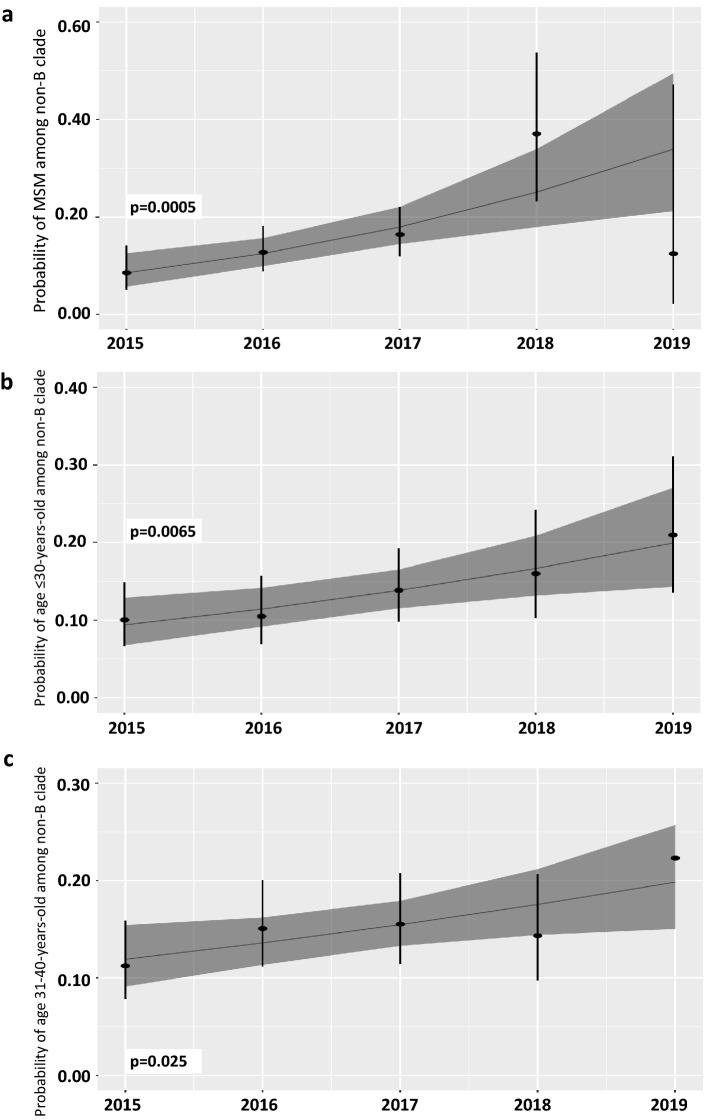
Logistic regression estimates for time trends between 2015–2019 for transmission category: men who have sex with men (MSM) (**a**), age at diagnosis for ≤ 30 years-old group (**b**) and 31–40 years-old group (**c**). Dots indicate the percentage per year and vertical bars 95% confidence intervals for the percentages with a vertically presented logistic regression time trend line with dark-grey shaded 95% confidence intervals for the regression estimate.

In non-B variant infected population, the proportion of the male gender increased steadily from 8.7% in 2015 to 17.4% in 2019 (OR: 1.20, 95% CI 1.09–1.32, p < 0.001—Fig. 9a). The frequency of non-B variants increased significantly from 10.3% in 2015 to 22.7% in 2019 among individuals aged < 30-years at diagnosis (OR: 1.24, 95% CI 1.06–1.46, p < 0.01—Fig. 9b). The percentage of the 31–40 age at diagnosis patients group increased from 11.3% in 2015 to 22.3% in 2019 (OR: 1.16, 95% CI 1.02–1.33, p < 0.05—Fig. 9c).

Additionally, the proportion of infections other than the B variants increased in asymptomatic cases from 12.1% in 2015 to 29% in 2018 and in AIDS patients from 6.5% in 2015 to 28.6% in 2018 (OR: 1.33, 95% CI 1.04–1.70, p < 0.05 and OR: 1.93, 95% CI 1.18–3.29, p < 0.01, respectively). In nearly half of the analyzed cases, data on the clinical category at diagnosis was missing. Accordingly, between 2015 and 2019, occurred a simultaneous increase of non-B infections occured in AIDS individuals and asymptomatic patients.

Also, among non-B variant infected individuals, the frequency of cases with lymphocyte CD4 count exceeding 200 cells/μl increased over time (from 12.5% in 2015 to 30.4% in 2018). The proportion of MSM was also significantly increased from 8.6 to 37.1% (OR: 1.53, 95% CI 1.20–1.95, p < 0.001. For the analysis of time trends of the last two parameters patients diagnosed in 2019 were excluded as their clinical data were not available.

## Discussion

Our results showed that increasing A-clade incidence in Poland is driven mostly by the heterosexual transmission mode, age at diagnosis, and female gender. The decline in subtype B has been observed in Europe, but the spread of HIV-1 variants is different in each country. This study is the first to characterise the cohort of A6 sublineage on Poland’s national scale. Together with evidence of novel A6/B URFs, this suggest considerable heterogeneity of HIV-1 in the polish population. Our results might support drug therapy interventions because infections with the A6 variant may bear resistance to cabotegravir, the long-acting antiviral soon to be implemented in the clinical practice^12^.

In this article, we delineated the distribution of HIV-1 variants in the period between 2015 and 2019 in a large group of diagnosed patients from Poland to reveal the countrywide molecular diversity of the virus. For the first time, based on phylogenetic studies, we report on the presence of the HIV-1 A6 sublineage in Poland. Subtype B remains the most common variant to date, shared by 85.9% of the surveyed population, and remains predominant among MSM. This observation is in line with our previous report on the period from 2008 to 2014, where 86.9% of recorded infections developed from HIV-1 B lineage^8^ and other Polish HIV epidemic studies^7,13,14^. However, the proportion of non-B clade increased significantly between 2015 and 2019, primarily due to increased number of infections with sublineage A6. The decline in subtype B has been observed in Europe. It may be driven by the economic migration and refugee crisis and the increasing circulation of non-B subtypes among the native European population^15–17^. Of note, in the past decade immigration in Poland was observed from the Former Soviet Union (FSU) nations, especially Ukraine^18^. Until the end of 2019, 1,351,418 Ukrainians lived in Poland, which constitutes 64% of all officially registered foreigners (https://stat.gov.pl/en). The increase in subtype A is likely to be related to immigration as this variant is ubiquitous in Eastern Europe^19^. Indeed, the highest percentage of the A6 variant have been recorded in northeast province, close to the former Soviet federation border (Supplementary Table 1 and Fig. 4). A6 sublineage was first identified among IDUs in the Ukrainian city of Odessa in late 1994, and was previously included in the A (FSU) subgroup. Immigration related HIV infection has remained common in Europe^20–22^. In Poland, up to date, a small percentage of immigrants (< 5%) have been observed among the HIV-1 diagnosed individuals, however, the observed data suggest introduction and spread of novel variants in the country^23^. Molecular surveillance over A6 variants is of importance due to possible emergence of resistance and virological failure after therapy containing the HIV-1 integrase strand transfer inhibitor cabotegravir^12,24^. In addition to A6 samples, ten samples of the A1 and one of the A3 variant were identified. The phylogeographic mixture of A sub-lineage variants has also been observed in Germany^22^ and Italy^20^.

Identification of three A/B recombination clusters is an important novel finding of this study. For the first time we identify two new clusters of A6B recombinant variants. Our former publication described one monophyletic group of A1B and a single sequence of A6B^8^. In the current analysis, A/B recombinants accounted for 5.9% of the non-B variants, while in the period 2008–2014, they were identified in 3.1% of sequences^8^. Thus, the frequency of mosaic sequences composed of subtypes A and B almost doubled over the analyzed years. Previously, we described one regional HIV center (Cracow) as the source of all clustered A1B sequences and the city of Bialystok as the origin of a single sample of the A6B mosaic form. In the present data, recombinant virus isolates were obtained from individuals followed up in seven cities, which are located across different provinces. Our analyses provide evidence that the described mosaic forms circulate in Poland and contribute to increasing genetic heterogeneity of non-B subtype strains. Although the sequences in A1B Cluster had similar recombination profile, the diversity of recombinant forms within the A6B Clusters was large. In A6B clusters, the number of recombination in the *pol* gene region vary from one up to four break events which reflect high recombination rate of HIV-1 variants. Since the subtype B and sub-subtype A6 are the most widespread in Poland, such a recombination was expected and might reflect the presence of a novel, yet unidentified CRF.

Cluster sequences may likely implicate transmission networks. The analysis of phylogenetic clusters, together with epidemiological and demographic data, are important to understand the factors underlying the growth of epidemics. Others, have published that MSM are more likely to cluster compared to all other risk groups^25^. Indeed, all sequences in the A/B clusters derived only from male individuals and apparently indicate the presence of MSM transmission group. However, there was a shortage of information relative to transmission mode for those patients. Furthermore, gender is presumably related to the differences in HIV-1 clade distribution within exposure groups^26^. The lack of women in the A/B recombination clusters probably reflect the MSM and/or IDU transmission mode. Both A6B clusters include A sequence regions with Eastern European ancestry. In the LANL-HIV CRF’s database (https://www.hiv.lanl.gov/content/sequence/HIV/CRFs/CRFs.html), only one example of subtype B and A recombination (CRF03_AB) has been identified so far.

The differences in demographic characteristics across the analyzed samples are due to the increase in the female gender and dominance of the heterosexual transmission route among individuals infected with non-B clades. Our results indicate that the male-to-female ratio in the non-B clade is 3.30 compared to 6.43 in subtype B. In the past five years of study, HIV-1 infections were mostly driven through MSM- and IDU-associated clades and as a consequence, predominance of men and subtype B were observed. Many authors have published similar findings^25,26^. It should be noted that the increase in the proportion of non-B clades among females is connected with migration and travel related introduction of infections, especially in sublineage A6 viruses. The ratio of male-to-female correlates with the number of non-B subtypes because more males have been diagnosed with subtype B. The Silesian and Lower Silesian provinces had a low incidence of non-B subtypes infections result in a high (above five) male-to-female ratio. South of these regions, a similar proportion was noted in the Czech Republic, where the male-to-female ratio elevates the European average^23^. As observed in Podlaskie and West Pomerania lower male-to-female ratio (under three) was correlated with frequent A6 subtype occurrence.

Interestingly and importantly, a significantly higher lymphocyte count at baseline among non-B variant infected individuals is in contrast to our previous records^8^. This is also associated with the increasing frequency of subtype A. Individuals infected with those virus strains clearly undergo care entry at the earlier stages of infection, with less pronounced immunodeficiency. Additionally, female gender possesses better immunovirological parameters, which is higher CD4 + T cells following primary HIV‐1 infection at the beginning of infection compared to males^27,28^.

Furthermore, among non-B subtype characteristics, notable time associated trends were observed. Firstly, the proportion of non-B clades among MSM is increasing. This is also reflected in the increasing frequency of males for non-B variants in the analyzed timeline, and may be associated with dense transmission networks and clustering of MSM^29^. Secondly, across HIV age at diagnosis, especially in young adults (under thirty and forty), non-B clade distribution is increased. Age at diagnosis is shifting towards younger individuals in non-B clades. This observation may correspond to the increasing trend in non-B variants frequency associated with MSM mode. Moreover, these findings could be related to less frequent testing of older heterosexual males.

Additionally, at the time of sampling differences in incidence among clinical symptoms in non-B variants were observed. Regardless of CDC clinical stage, approximately half of the data was missing in available clinical reports. Therefore, the representation of non-B clade asymptomatic and AIDS patients could mutually increase in the timeframe of the study. Patient clinical statuses at diagnosis vary due to prevention efforts and time of enrollment in care. The larger frequency of CD4 + cell count above 200 cells/μl at baseline and the diagnosis of patients at a younger age in the non-B clade may be associated with a higher proportion of registered asymptomatic patients. Moreover, a higher level of lymphocytes at HIV-1 diagnosis is noted in women than in men^28^. This point may explain the increase in non-B individual’s frequency associated with higher lymphocyte count as females were notably more common among non-B subtypes.

On the other hand, we observed a growing prevalence of AIDS individuals within non-B variants. This result is in line with previous findings that in Poland, 44.8% of newly diagnosed patients are diagnosed late (LP) or in the AIDS stage. Factors associated with LP/AIDS are older age, IDU, and HTX^30^. Diagnosis several years after an infection led to declining levels of CD4 + cells at the time of sampling. Nevertheless, cell count below 200 per μl, when AIDS is diagnosed, remains in a quarter of newly registered patients^31^.

According to the "HIV/AIDS surveillance in Europe 2019" report, the highest age-specific HIV diagnosis values in both genders is among the 25–39 years old group. The results of our research meet these data. In the present study, the under thirty-years age group was the most prevalent and the under forty-years age group followed. Moreover, the total male to female ratio in the current studied population was 5.77, almost identical to the value in central Europe (5.6). Unfortunately, the above report did not include the subtype distribution^23^.

The principal limitation of the study was the method of sampling. Individuals submitted for the report were originated from HIV-1 Regional Centers for primary transmitted drug-resistance or treatment failure analysis and may not fully reflect the prevalence of infections in the overall population. Nevertheless, the data obtained are based on a significant number of cases. Samples collected for our study come from 10 out of 16 provinces inhabited by 65% of the population (https://stat.gov.pl/en). Furthermore, we did not have access to complete patient’s documentation, so some parameters possessed fewer representations but still allowed for statistical modelling. The subtyping analyses performed in this study were based on only a fragment of the *pol* gene. Unfortunately, this makes detailed analysis of the sequence recombination profile impossible. Full HIV-1 sequencing would most likely allow to identify a higher number of recombinants and confirm the breakpoints in the identified ones. Furthermore, extensive characterization of the whole genome of the identified AB recombinants could provide valuable information about novel circulating variants.

Subtype B remains the predominant HIV-1 variant in Poland. However, there was a significant increasing trend in the prevalence of non-B clades, mostly due to a rising number of sublineage A6 HIV-1 infections. Our results showed a high frequency of recombinations between these two most prevalent subtypes and a small number of other URFs. We have identified three separate A/B sequence clusters, and the formation of novel recombinants is evidence of increased HIV-1 heterogeneity. The comparison of subtypes and transmission groups with demographic parameters suggests that HIV-1 disease is highly diverse. Non-B variants are associated with heterosexual transmission, age at diagnosis, and female gender. The study will be further extended as the identification of the novel recombinants is part of molecular surveillance for HIV-1 and may influence drug susceptibility.

## Methods

### Study group

We used 2518 samples collected from patients who undergo genotypic drug resistance testing in 10 of 27 Regional AIDS Centers for this study (cities: Bialystok, Bydgoszcz, Cracow, Chorzow, Gdansk, Lodz, Poznan, Szczecin, Wroclaw, Zielona Gora). The dataset included sequences obtained from both naive (84.3%) and treatment-experienced patients (15.7%) (one sequence per individual; if multiple sequences were available the earliest one was included in the analysis). The time frame of sampling was from 2015 to 2019. Plasma samples were collected locally and shipped to the genotyping laboratory. The collected data included gender, CD4 + lymphocyte count, HIV viral load at sampling, age at diagnosis (age at first positive HIV confirmatory test), transmission route (self-defined by the patient) and WHO clinical stage at sampling. HIV-1 RNA isolation and sequencing of the reverse transcriptase (RT) and protease (PR) domains were carried out using the ViroSeq HIV-1 Genotyping System v 2.0 (Abbot Molecular, Des PlainesIL, USA). The integrase region was sequenced using the methodology described by Laethem et al.^32^. Amplicons obtained by the nested PCR method were used for Sanger sequencing using the BigDye technology on an ABI 3500 platform (Applied Biosystems, Foster City, CA, USA). Integrase sequence assembly was performed with the Recall online tool^33^. All samples were sequenced at the Clinical Laboratory at the Department of Infectious, Tropical Diseases and Immune Deficiency at Pomeranian Medical University in Szczecin, Poland. PR/RT sequences were available for all samples, with addition of the integrase coding region for 28 (1.1%) samples.

### Ethics statement

The protocol of the study was approved by the Bioethical Committee of the Pomeranian Medical University, Szczecin, Poland, approval number (no. KB-0012/26/17 and KB-0012/08/12). All patients gave informed consent to the proceeding of the sample and clinical data processing to conduct this study. All samples in this study were de-identified to maintain participants anonymity. The research was conducted in accordance with the Declaration of Helsinki.

### Subtyping and phylogenetic analyses

The 1302 nucleotide fragments of the *pol* gene were used for subtyping. This region includes the protease and part of the reverse transcriptase sequence (corresponding to HXB2 genome location positions from 2253 to 3554). When sub-lineage breakpoints were detected, we also performed sequencing of the integrase 866 bp-long fragments (covering sites 4230–5096 of the HXB2 genome). All sequences were initially assessed with REGAv3 (http://www.bioafrica.net/typing-v3/hiv), COMETv2^34^, SCUEAL^35^, and Stanford subtyping tools^36^ and confirmed by phylogenetic analyses. For phylogenetic inference, sequences were aligned with Clustal Omega tool^37^. The GTR + I + G model with four gamma categories was selected as optimal for the analysed dataset using Modeltest-NG 0.1.3 software^38^. The calculated nucleotide frequencies using this model were as follows: freqA = 0.4373, freqC = 0.1556, freqG = 0.1840, freqT = 0.2228, gamma shape parameter = 0.6193, and p-inv = 0.2462. Reference sequences from LANL-HIV-1 compendium 2017 version were used for subtyping, and the dataset was further supplemented with sequences with high similarity (> 95%) from BLAST (Basic Local Alignment Search Tool) analysis. The DAMBE program was exploited for duplicates removal^39^. Phylogenetic trees were generated using PHYML v3.0 web-server (http://www.atgc-montpellier.fr/phyml/) with the maximum likelihood (ML) method, Nearest Neighbor Interchange (NNI) type of tree rearrangement and using likelihood ratio test (aLRT) based on a Shimodaira-Hasegawa-like procedure^40^. First, the trees for subtype B and A were inferred, subsequently non-B and non-A variants as well as other recombinants were analyzed. Breakpoints were identified using two methods: the jumping-profile hidden Markov model (jpHMM)^41^ and the Simplot v3.5.1 software using bootscanning with 300 bp window size and 20 bp increments, based on the NJ method using the 2-parameter Kimura model^42^. Mosaic viruses were confirmed by phylogenetic trees (PhyML online tool) containing breakpoint fragments with reference sequences to recognize parental subtypes. For alignment, fragments were treated as separate sequences with different bp length. Alignment was made to the reference sequences A-D, F–H, J, K. The length of the reference sequences was 2844 bp, which span continuously from the beginning of the PR gene to the end of the IN gene (2253–5096 bp of HXB2 genome) of the *pol* region. Thus, the phylogenetic analysis for the breakpoints fragments was independent of each other. Empty spaces in the analysed fragments were indicated as a "period" symbol. Such alignment serves to infer the phylogenetic tree with GTR + I + G model as described above. All trees have been prepared in the Interactive Tree of Life (iTOL)^43^. Trees were rooted with group O, however, this root was removed from the final figures.

### Statistical analyses

Statistical comparisons were performed with Fisher's exact and X^2^ test for nominal variables, as needed. The Mann–Whitney U-test was used to analyze continuous variables (lymphocyte counts, HIV viral load, age at diagnosis). Interquartile ranges (IQR) and confidence intervals (CI) were marked as appropriate. Statistical calculations were made with commercial software (TIBCO Software Inc. 2019. *Statistica Software: Release 13.6.* Palo Alto, CA: TIBCO Software Inc). Time trends and logistic regression were performed with the R (4.0.1.)^44^ platform package MASS^45^ (coding available on request).

## Supplementary Information

Supplementary Information 1.

Supplementary Information 2.

Supplementary Information 3.

Supplementary Information 4.

Supplementary Information 5.

Supplementary Information 6.

Supplementary Information 7.

Supplementary Information 8.

Supplementary Information 9.

Supplementary Information 10.

## Data Availability

Submission of almost all regional cohorts to public databases would allow identification and risk violation of patient confidentiality. We have followed earlier practice^46^, and submitted a random sample of 10% of each regional HIV Centre sequence to GenBank. The sequences can be found under the appropriate IDs: MZ468643—MZ468894. Additionally, sequences for the identified AB recombinant clusters were deposited under following accession numbers: MZ671788-MZ671823.
