# Supplementary material for: Molecular epidemiology and HIV-1 variant evolution in Poland between 2015 and 2019
Source: Sci Rep. 2021 Aug 16;11:16609. doi: 10.1038/s41598-021-96125-w (PMC8367969; doi:10.1038/s41598-021-96125-w)
Supplement: Supplementary file 1 — Supplementary Information 1. [file 41598_2021_96125_MOESM1_ESM.docx]

**Supplementary figures legends:**

**Supplementary Figure S1.** Phylogenetic tree inferred using the maximum likelihood method with HIV reference from HIV sequence compendium 2017, supplemented with sequences (of partial 1302 bp HIV-1 *pol* gene) for all non-B, non-A, with the exception of the URF variants that were found in the studied population. Branches containing the same HIV-1 clade have been collapsed. The tree were rooted with group O, however, this root was removed from the final figure. The Figure was made using iTol (43).

**Supplementary Figure S2.** Phylogenetic tree of partial HIV-1 *pol* sequences (1302 bp) inferred using ML method with HIV-1 reference from HIV sequence compendium 2017, supplemented with HIV-1 sequence database-deposited sequences for recombinants of subtypes different than A and B with 8 unique recombinant forms (composed of mosaic structures other than A and B variants) found in the studied population. Purple highlight indicates BF1 cluster sequences. The black triangles mark the URF sequences found in studied population. Branches containing the same HIV-1 clade have been collapsed. Approximate likelihood values are shown at the tree nodes. The tree were rooted with group O (accession no KY953205), however, this root was removed from the final figure. The Figure was made using iTol (43).

**Supplementary Figure S3.** Bootscanning analysis of the five partial HIV-1 pol sequences from the identified A6B Cluster 1. Analyses were conducted using a window size of 300 bp and step size of 20 bp along with reference variants of the representative sublineages A6, B, and F1 (as an out-group). Implemented in Simplot v3.5.1 software.

**Supplementary Figure S4.** Subtyping analyses of the five partial HIV-1 pol sequences from the identified A6B Cluster I. Phylogenetic analyses (ML tree) according to breakpoints located within the position 2253–5096 bp of HXB2 genome in the *pol* region. Subtype regions are labeled from 1 up to 5 according to breakpoints events on genomic map. Amongst A6 and B subtypes monophyletic clustering of identified regions was indicated. ML tree inferred with HIV-1 reference subtypes from HIV sequence compendium 2017. Branches containing the same HIV-1 clade have been collapsed. The Figure was made using iTol (43).

**Supplementary Figure S5.** Bootscanning analysis of the nine partial HIV-1 pol sequences from the identified A6B Cluster 2. Analyses were conducted using a window size of 300 bp and step size of 20 bp along with reference variants of representative sublineages A6, B and F1 or C (as an out-group). Implemented in Simplot v3.5.1 software.

**Supplementary Figure S6.** Subtyping analyses of the nine partial HIV-1 pol sequences from the identified A6B Cluster 2. Phylogenetic analyses (ML tree) according to breakpoints located within position 2253–5096 bp of HXB2 genome in the *pol* region. Subtype regions are labeled from 1 up to 3 according to breakpoints events on genomic map. Amongst A6 and B subtypes monophyletic clustering of identified regions was indicated. ML tree inferred with HIV-1 reference subtypes from HIV sequence compendium 2017. Branches containing the same HIV-1 clade have been collapsed. The Figure was made using iTol (43).

**Supplementary Figure S7.** Bootscanning analysis of the seven partial HIV-1 pol sequences from the identified A1B Cluster. Analyses were conducted using a window size of 300 bp and step size of 20 bp along with reference variants of representative sublineages A1, B and K (as an out-group). Implemented in Simplot v3.5.1 software.

**Supplementary Figure S8.** Subtyping analyses of the seven partial HIV-1 pol sequences from the identified A1B Cluster. Phylogenetic analyses (ML tree) according to breakpoints located within position 2253–5096 bp of HXB2 genome in the *pol* region. Subtype regions are labeled from 1 to 4 according to breakpoints events on genomic map. Amongst A1 and B subtypes monophyletic clustering of identified regions was indicated. ML tree inferred with HIV-1 reference subtypes from HIV sequence compendium 2017. Branches containing the same HIV-1 clade have been collapsed. The Figure was made using iTol (43).
