# Supplementary material for: Molecular epidemiology and HIV-1 variant evolution in Poland between 2015 and 2019
Source: Sci Rep. 2021 Aug 16;11:16609. doi: 10.1038/s41598-021-96125-w (PMC8367969; doi:10.1038/s41598-021-96125-w)
Supplement: Supplementary file 2 — Supplementary Information 2. [file 41598_2021_96125_MOESM2_ESM.docx]

|  | Subtype A | | Subtype B | | Subtype C | | Subtype D | | Subtype F1 | | Subtype G | | CRF01_AE | | CRF02_AG | | Other CRFs & URFs (nonAE, nonAG) | | Total non-B | |
| --- | --- | --- | --- | --- | --- | --- | --- | --- | --- | --- | --- | --- | --- | --- | --- | --- | --- | --- | --- | --- |
| Region (city of HIV centre) | n (%) | p* | n (%) | p* | n (%) | p* | n (%) | p* | n (%) | p* | n (%) | p* | n (%) | p* | n (%) | p* | n (%) | p* | n (%) | p* |
| **Upper Silesia** (Chorzów) n = 564 | 22 (3.90) | **<0**.**001** | 529 (93.79) | **<0**.**001** | 5 (0.89) | 0.70 | 0 | **0**.**0018** | 0 | 1 | 0 | 0.35 | 0 | 0.21 | 1 (0.18) | 0.32 | 7 (1.24) | 0.30 | 35 (6.21) | **<0**.**001** |
| **Lower Silesia** (Wrocław) n = 525 | 28 (5.33) | **<0**.**001** | 477 (90.86) | **<0**.**001** | 5 (0.95) | 0.84 | 3 (0.57) | 0.34 | 0 | 1 | 0 | 0.35 | 0 | 0.22 | 3 (0.57) | 0.74 | 9 (1.71) | 1 | 48 (9.14) | **<0**.**001** |
| **Lesser Poland** (Kraków) n = 445 | 60 (13.48) | **<0**.**001** | 360 (80.90) | **<0**.**001** | 8 (1.80) | **0**.**078** | 0 | **0**.**0091** | 2 (0.45) | 0.031 | 1 (0.2) | 1 | 1 (0.22) | 1 | 2 (0.45) | 1 | 11 (2.47) | 0.20 | 85 (19.10) | **<0**.**001** |
| **Pomeranian** (Gdańsk) n = 310 | 38 (12.26) | **0**.**011** | 255 (82.26) | **0**.**049** | 2 (0.65) | 0.76 | 0 | 0.068 | 0 | 1 | 2 (0.65) | 0.16 | 4 (1.29) | 0.01 | 1 (0.3) | 1 | 8 (2.6) | 0.23 | 55 (17.7) | **0**.**049** |
| **West Pomeranian** (Szczecin) n = 299 | 45 (15.05) | **<0**.**001** | 222 (74.25) | **<0**.**001** | 3 (1.00) | 1 | 21 (7.02) | **<0**.**001** | 0 | 1 | 1 (0.33) | 0.53 | 2 (0.67) | 0.24 | 3 (1.00) | 0.19 | 2 (0.67) | 0.16 | 77 (25.75) | **<0**.**001** |
| **Greater Poland** (Poznań) n= 110 | 9 (8.18) | 0.73 | 97 (88.18) | 0.48 | 0 | 0.63 | 0 | 0.63 | 0 | 1 | 1 (0.91) | 0.24 | 0 | 1 | 0 | 1 | 3 (2.73) | 0.44 | 13 (11.82) | 0.48 |
| **Lubusz** (Zielona Góra) n = 103 | 11 (10.68) | 0.57 | 87 (84.47) | 0.70 | 0 | 0.62 | 1 (0.97) | 1 | 0 | 1 | 0 | 1 | 0 | 0.56 | 3 (2.91) | **0**.**003** | 0 | 1 | 15 (14.56) | 0.67 |
| **Podlaskie** (Białystok) n = 97 | 15 (15.46) | **0**.**011** | 76 (78.35) | **0**.**029** | 3 (3.09) | 0.076 | 2 (2.06) | 0.28 | 0 | 1 | 0 | 1 | 0 | 1 | 0 | 1 | 1 (1.03) | 1 | 21 (21.65) | **0**.**03** |
| **Kuyavian-Pomeranian** (Bydgoszcz) n = 34 | 0 | **0**.**068** | 32 (94.12) | 0.17 | 0 | 1 | 0 | 1 | 0 | 1 | 1 (2.94) | 0.078 | 1 (2.94) | 0.10 | 0 | 1 | 0 | 1 | 2 (5.88) | 0.17 |
| **Łódź** (Łódź) n = 31 | 1 (3.26) | 0.36 | 28 (90.32) | 0.48 | 0 | 1 | 0 | 1 | 0 | 1 | 0 | 1 | 0 | 1 | 0 | 1 | 2 (6.45) | 0.10 | 3 (9.68) | 0.48 |

**Supplementary Table 1.** Differences in HIV variant prevalence by analyzed region. Statistical differences calculated for the regional subtype/recombinant form distribution versus the entire sample of 2518 patients. Calculated by X^2^ test, for samples <5 Fisher exact test was used
