# Supplementary figures and images for: Molecular epidemiology and HIV-1 variant evolution in Poland between 2015 and 2019
Source: Sci Rep. 2021 Aug 16;11:16609. doi: 10.1038/s41598-021-96125-w (PMC8367969; doi:10.1038/s41598-021-96125-w)

## Colored ranges

- C  
07\_BC  
F1  
06\_cpx  
G  
02\_AG  
01\_AE  
D  
03\_AB  
53\_01B  
56\_cpx  
42\_BF  
47\_BF  
12\_BF  
60\_BC

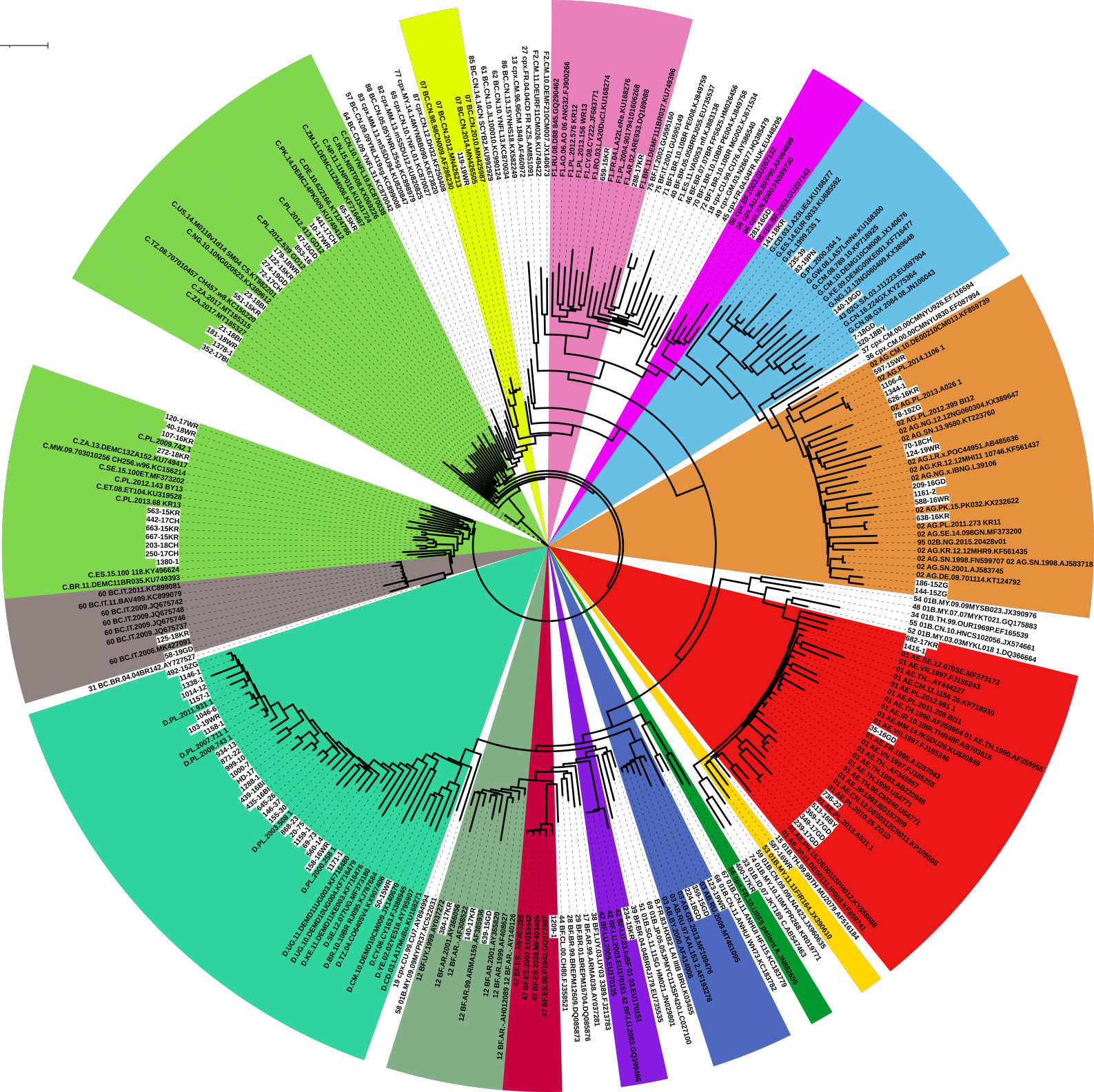

Supplement: Supplementary file 3 — Supplementary Information 3. [file 41598_2021_96125_MOESM3_ESM.pdf]

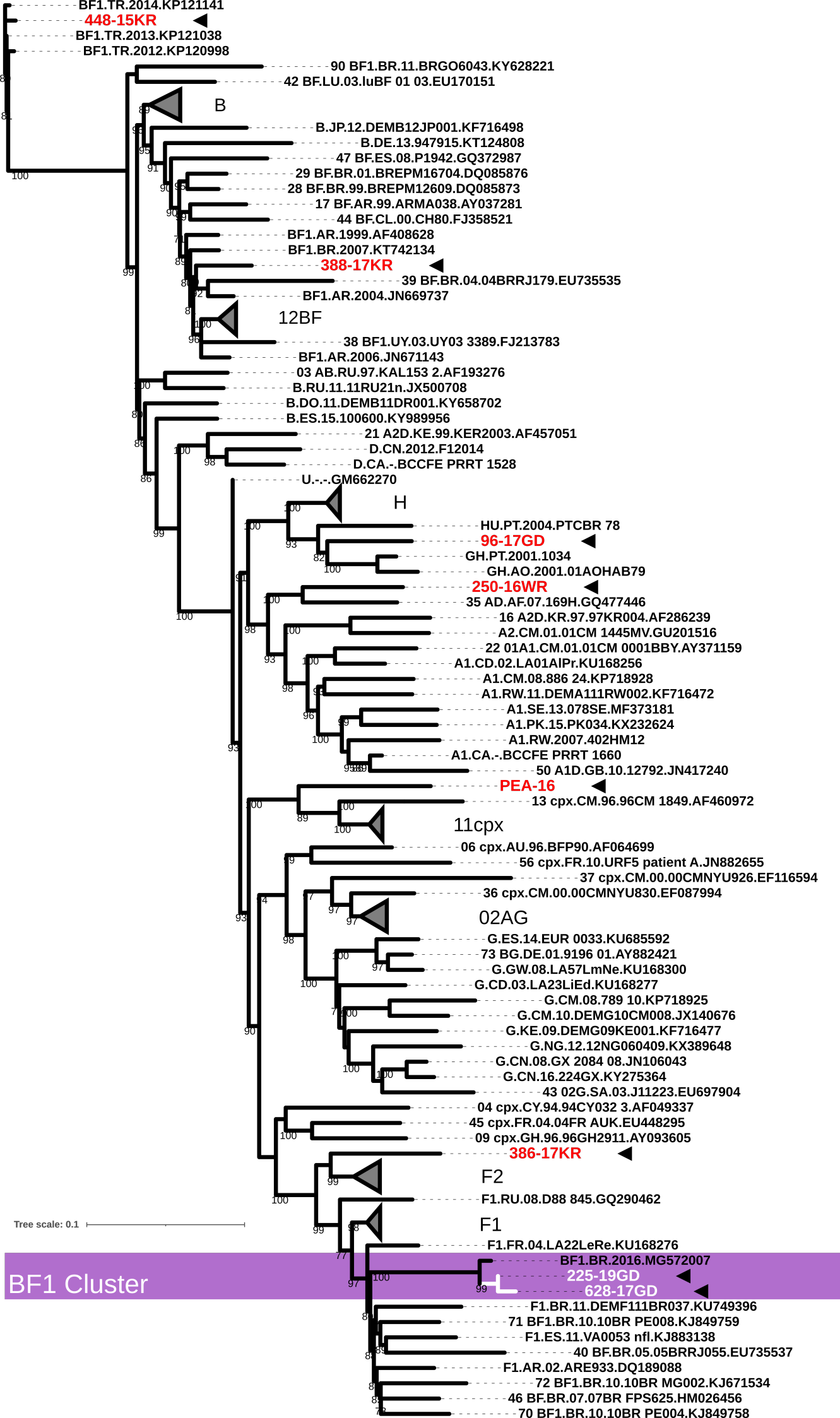

Supplement: Supplementary file 4 — Supplementary Information 4. [file 41598_2021_96125_MOESM4_ESM.pdf]

## A6

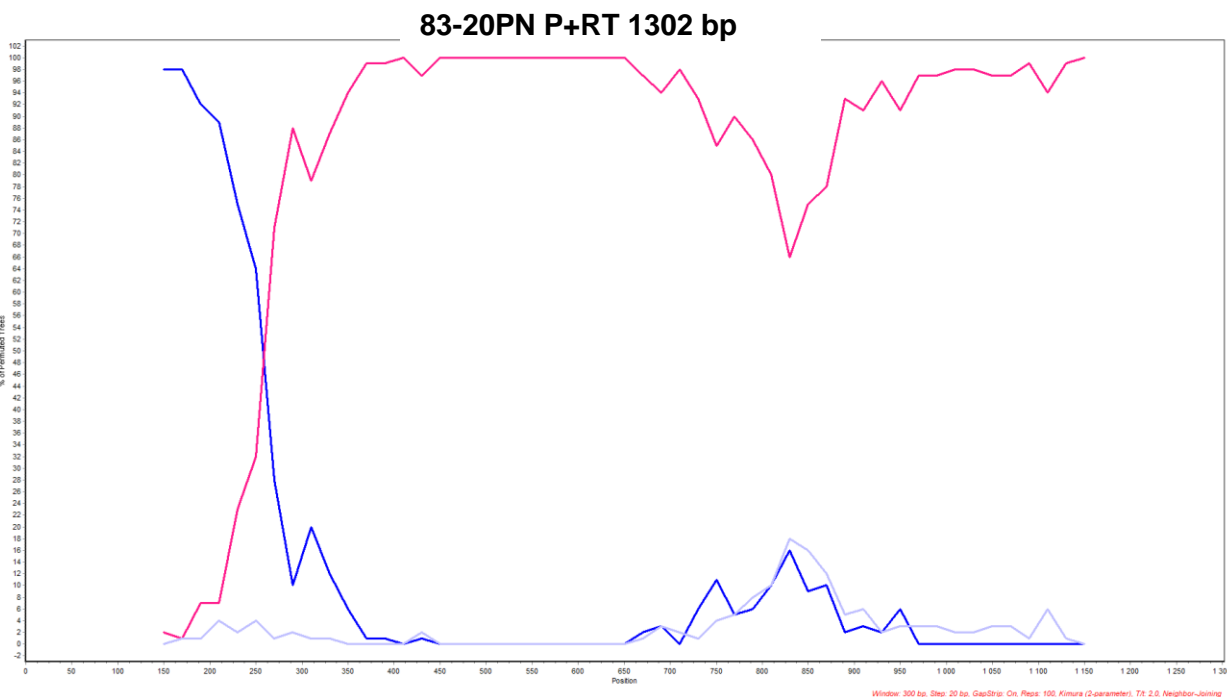

B

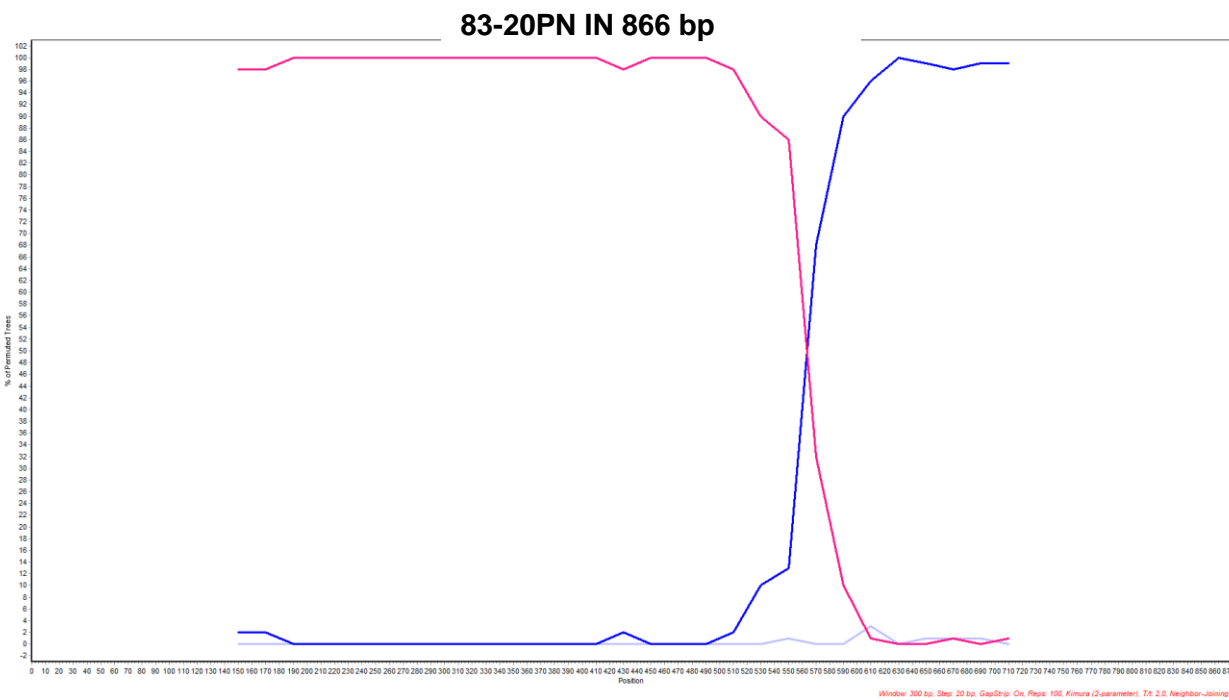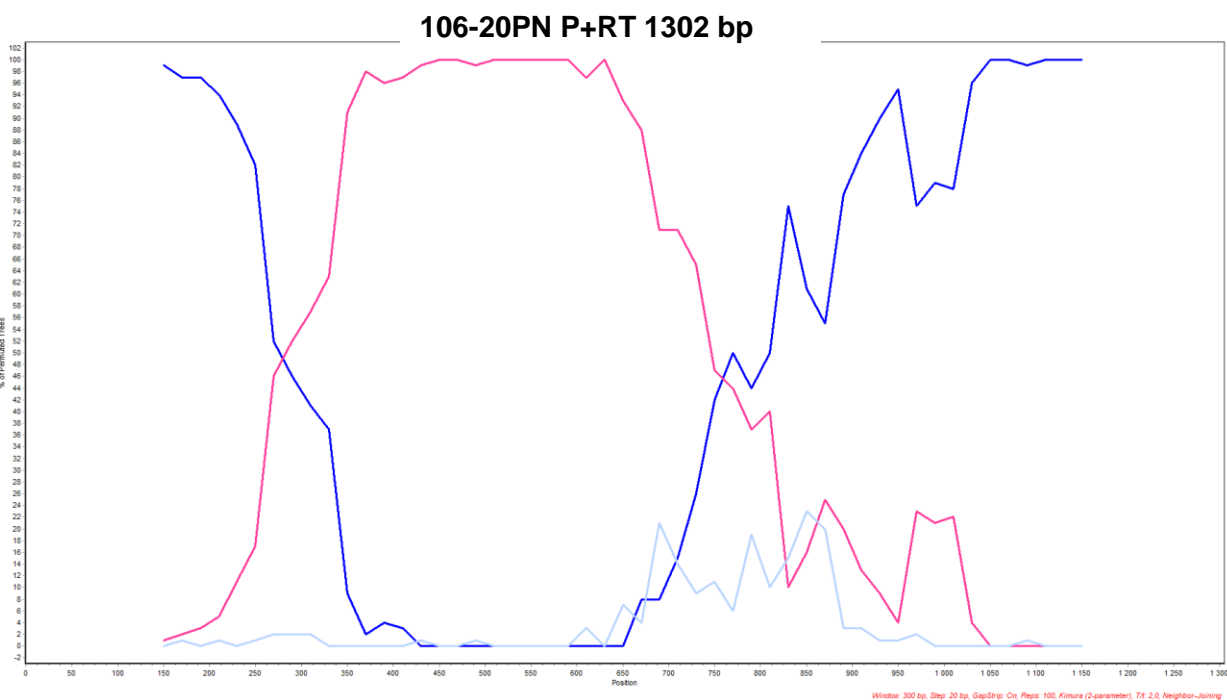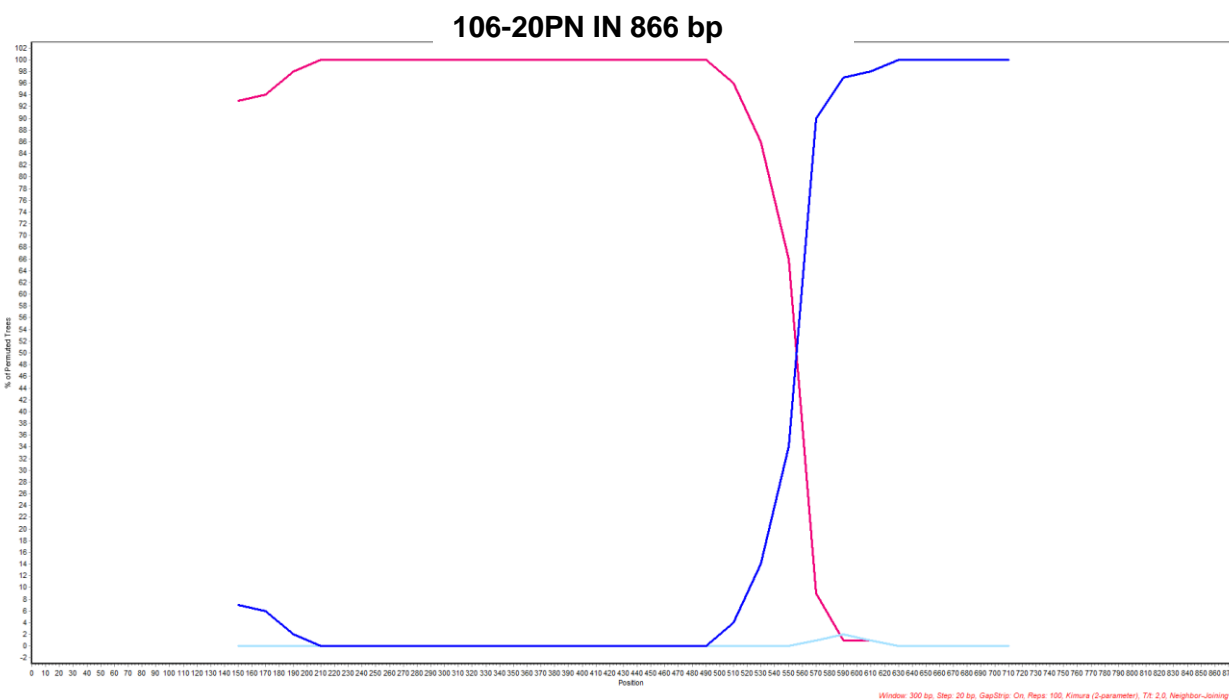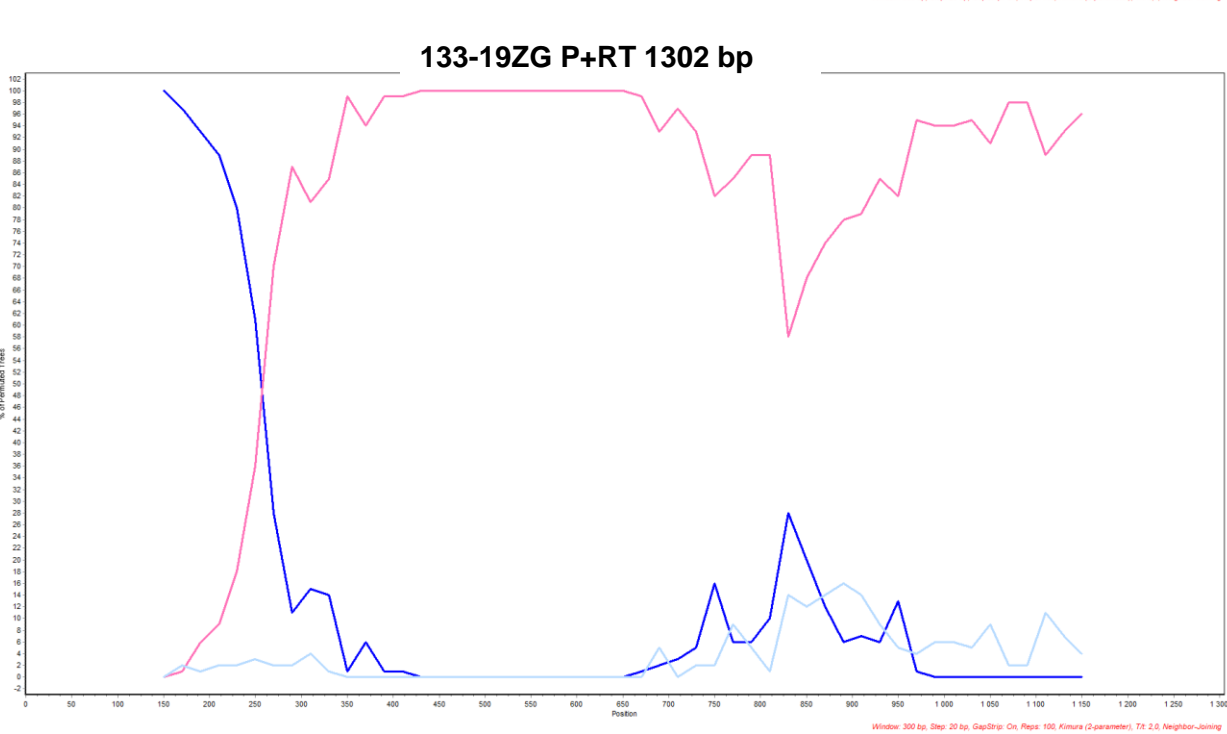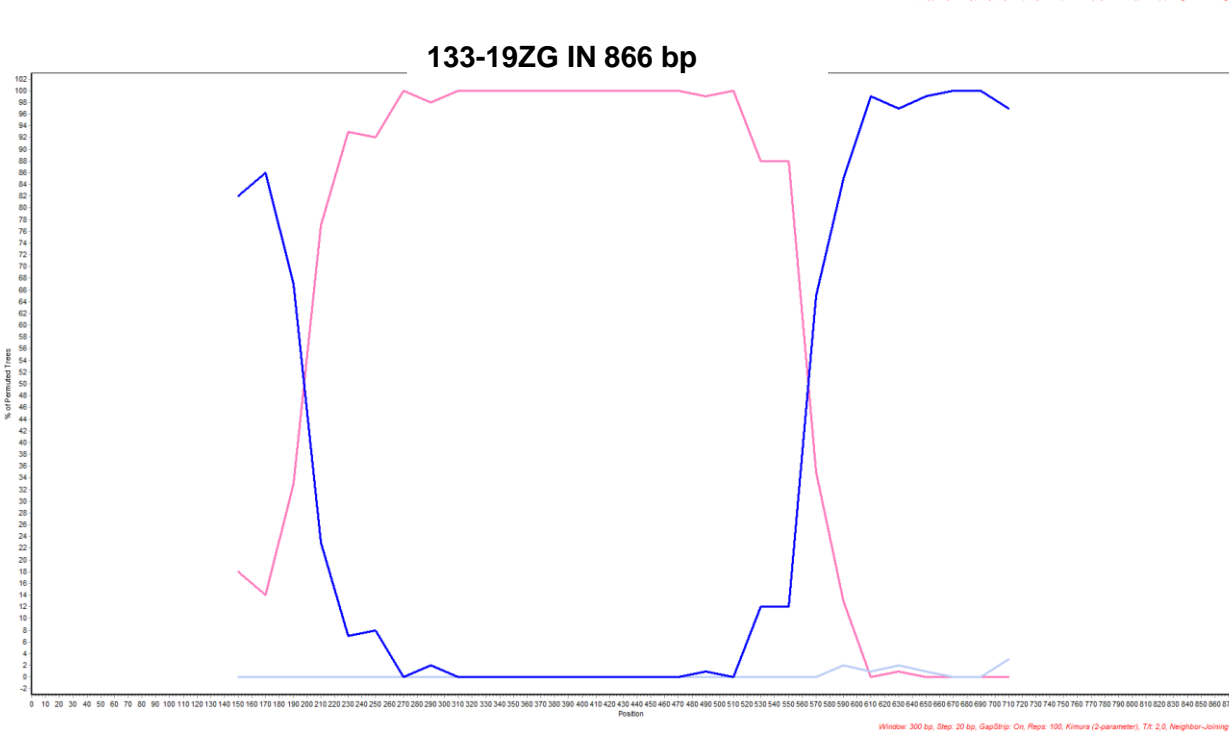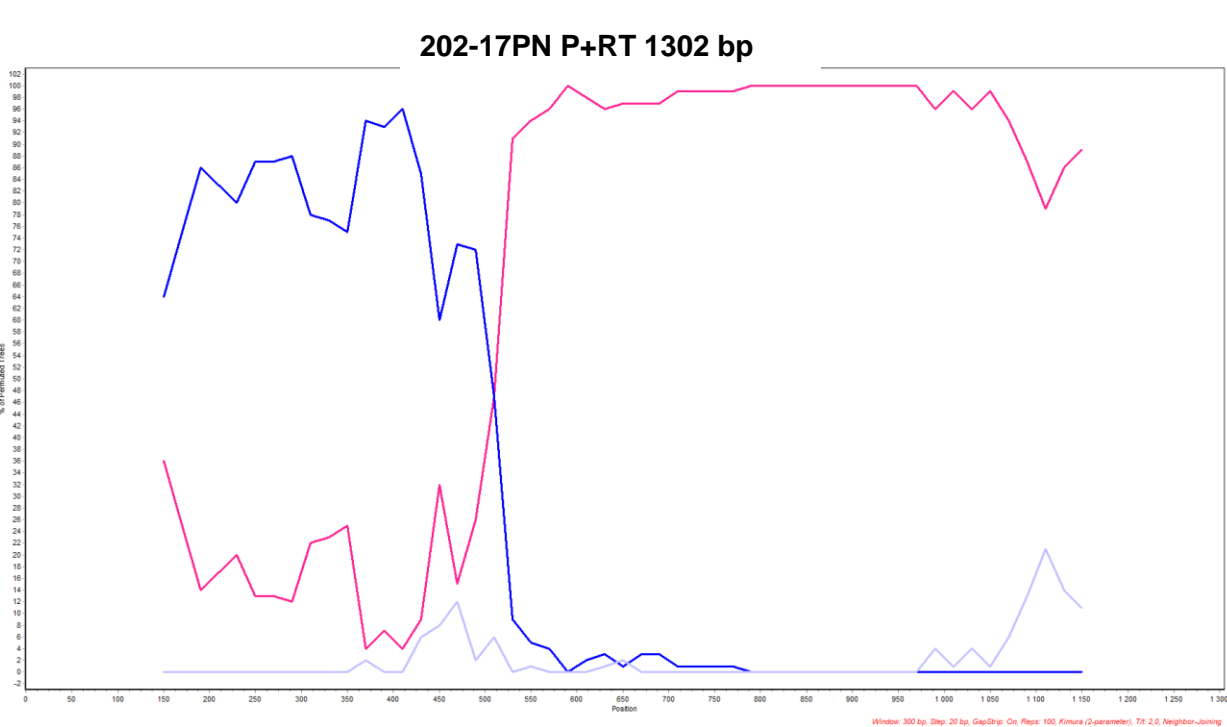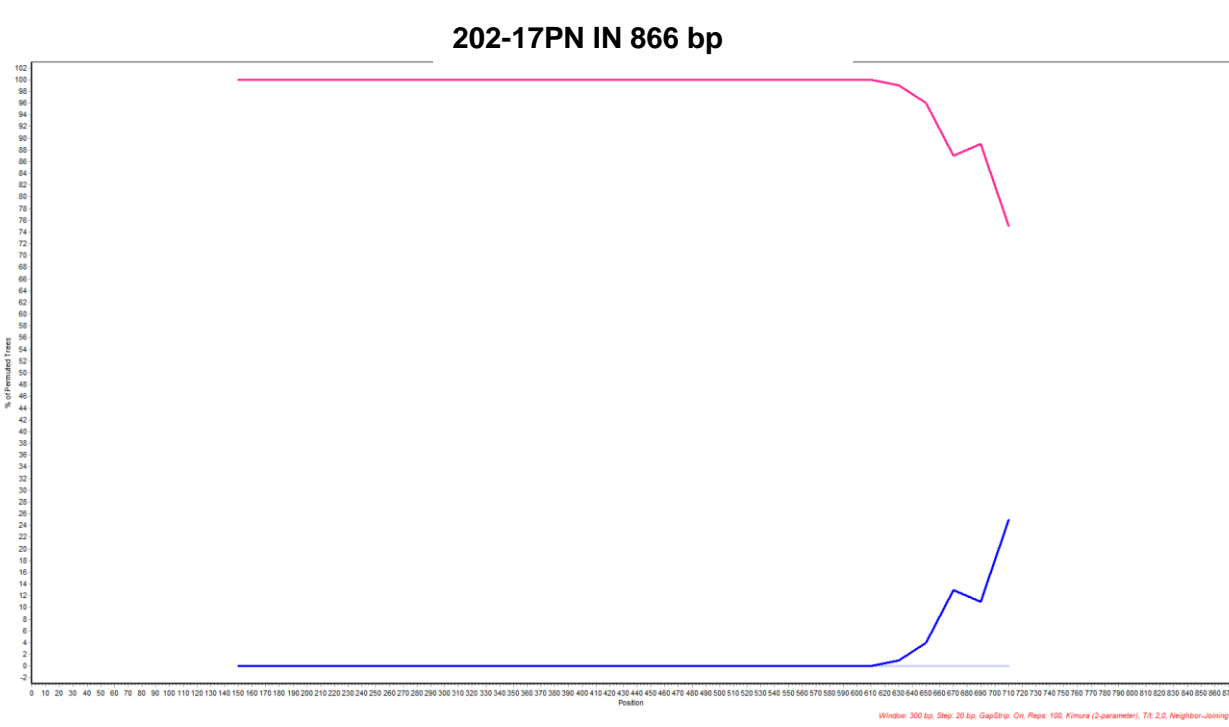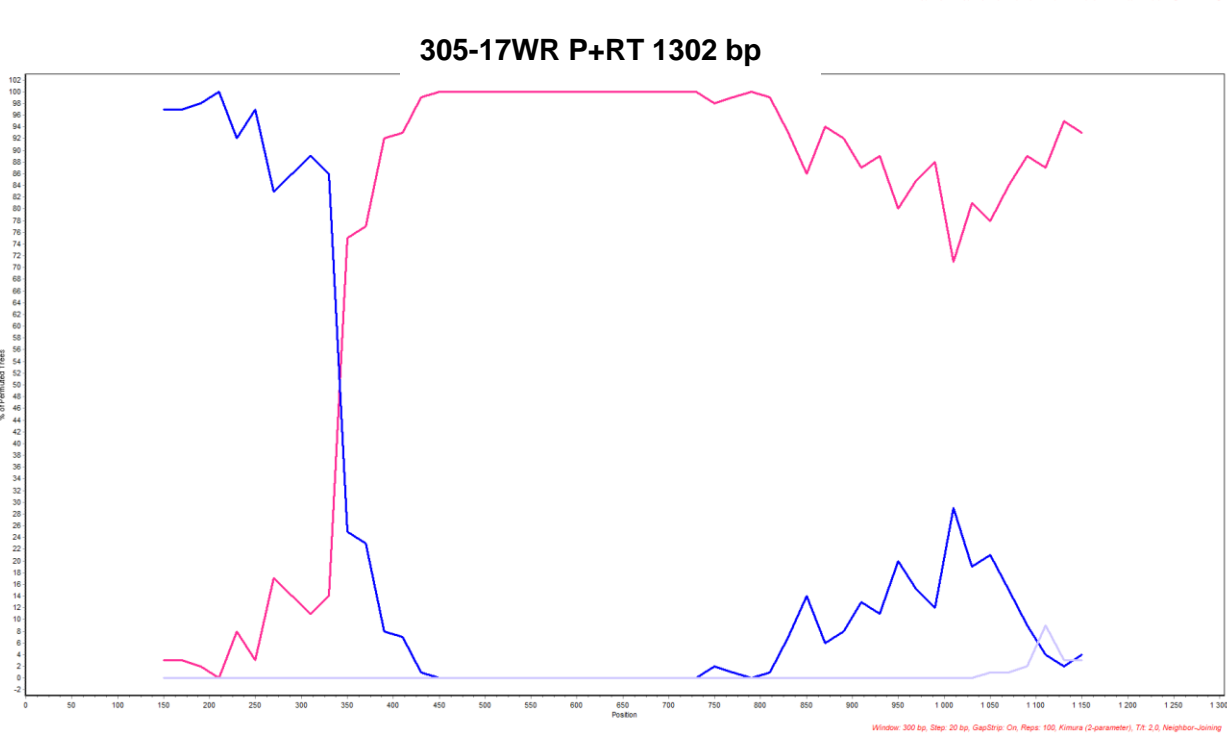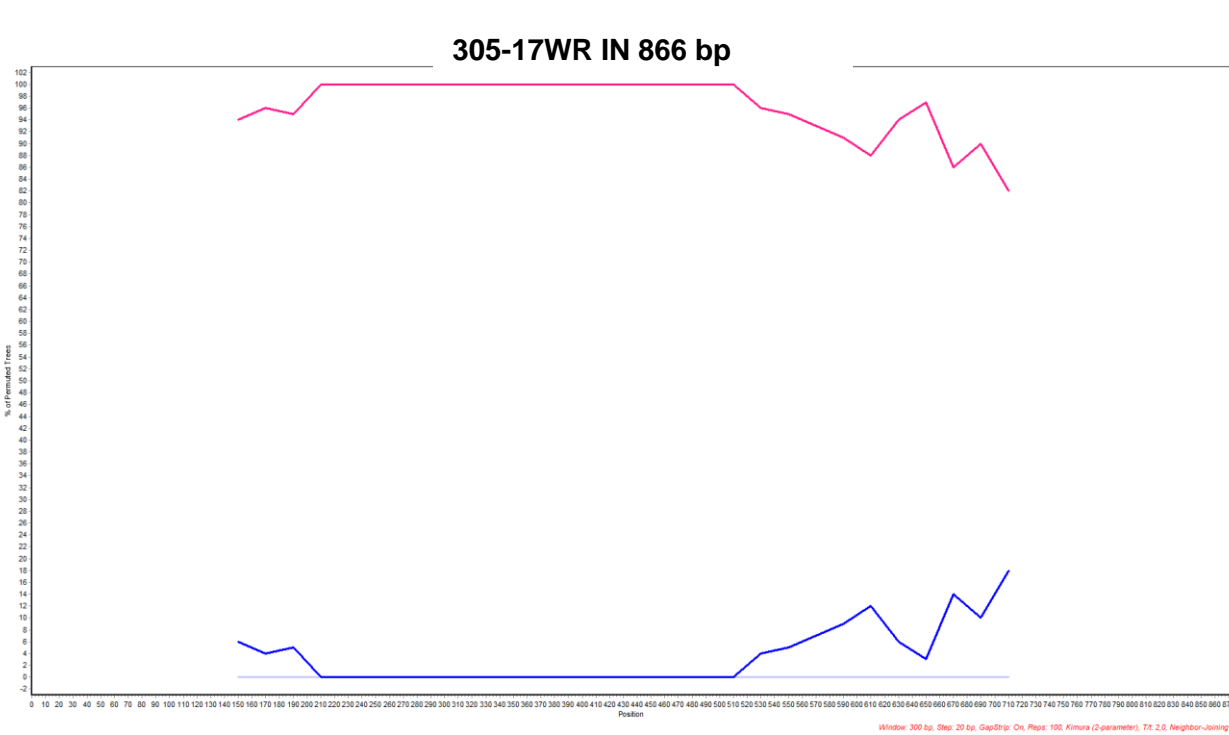

Supplement: Supplementary file 5 — Supplementary Information 5. [file 41598_2021_96125_MOESM5_ESM.pdf]

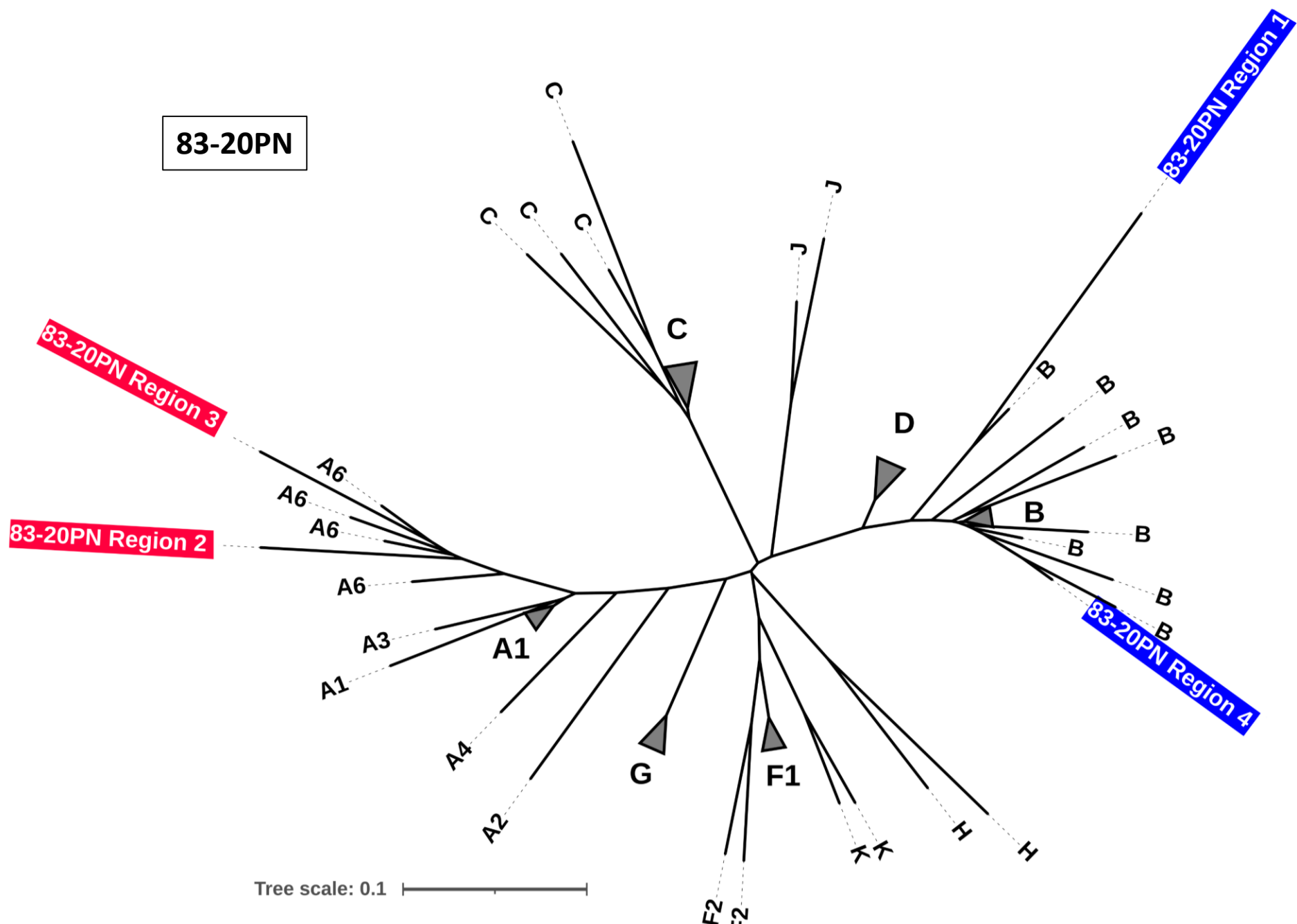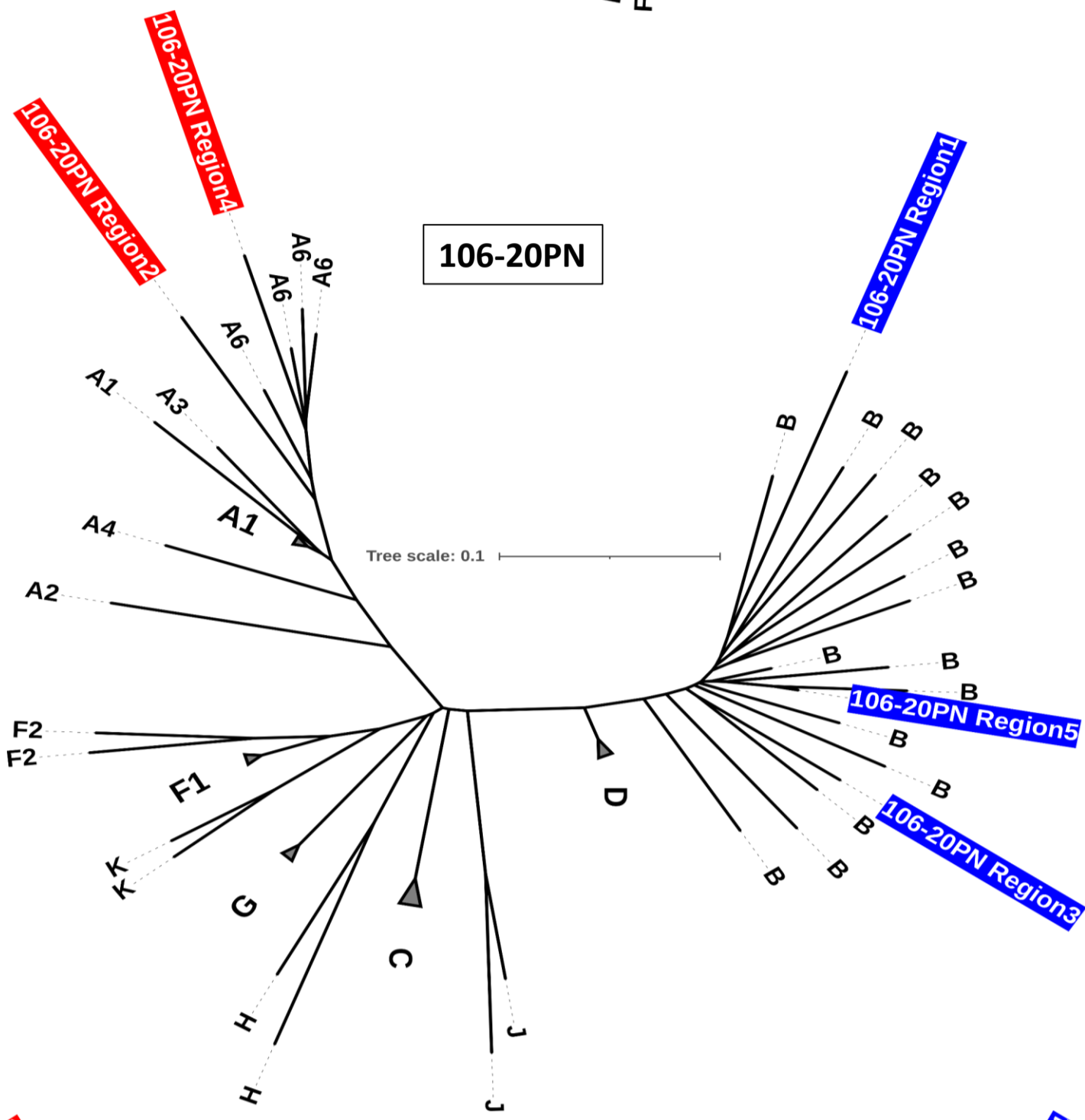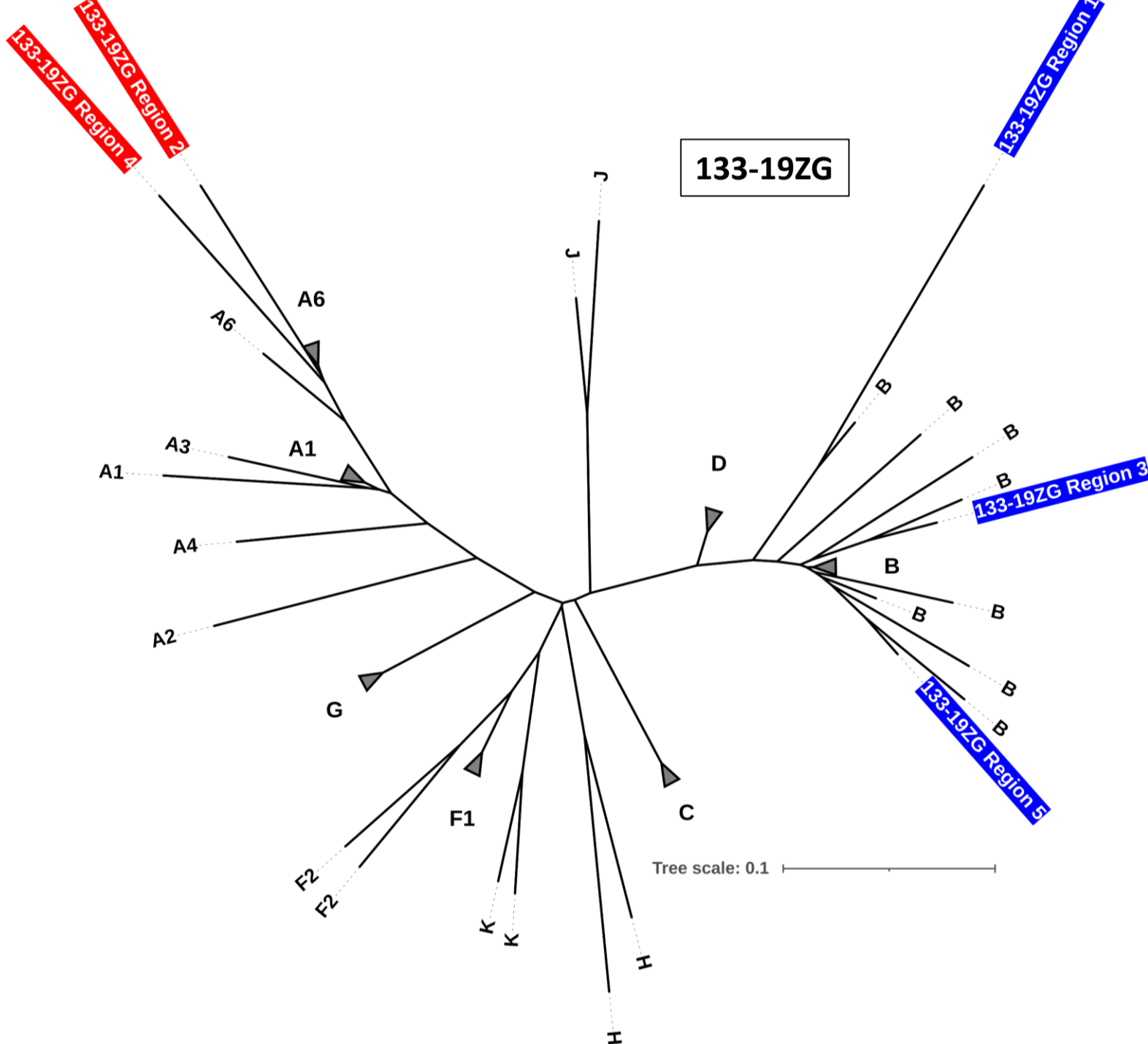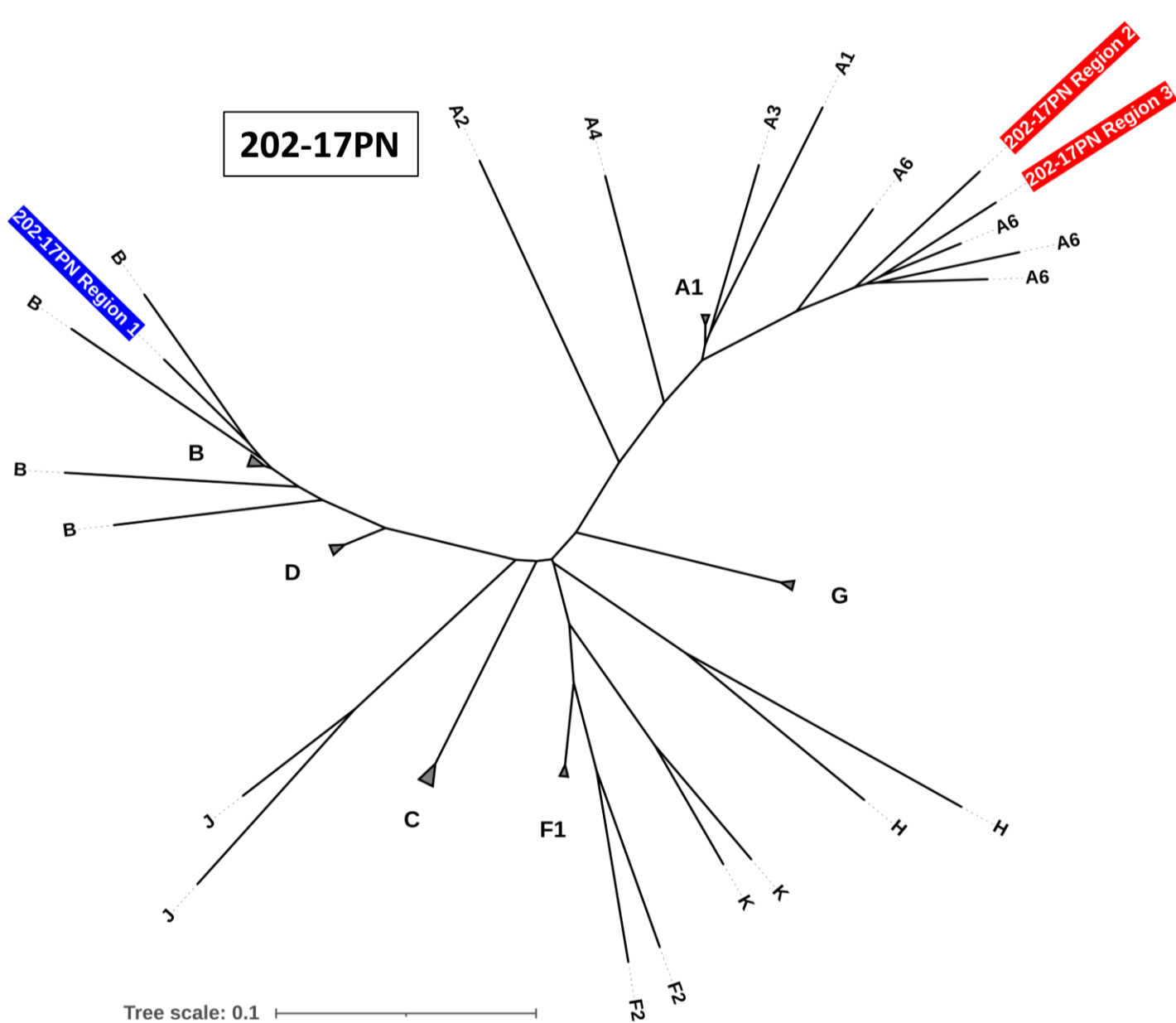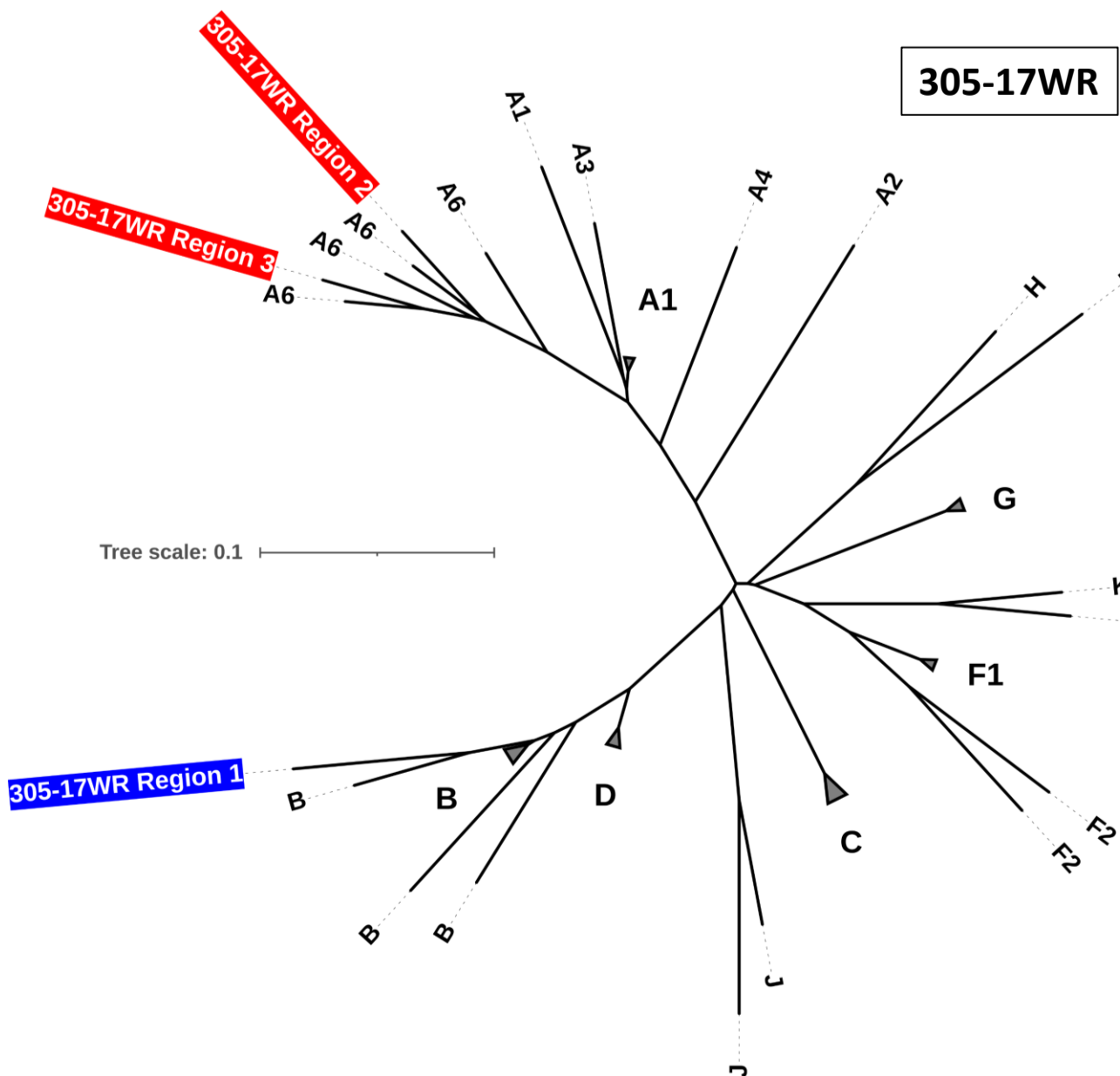

Supplement: Supplementary file 6 — Supplementary Information 6. [file 41598_2021_96125_MOESM6_ESM.pdf]

A6

B

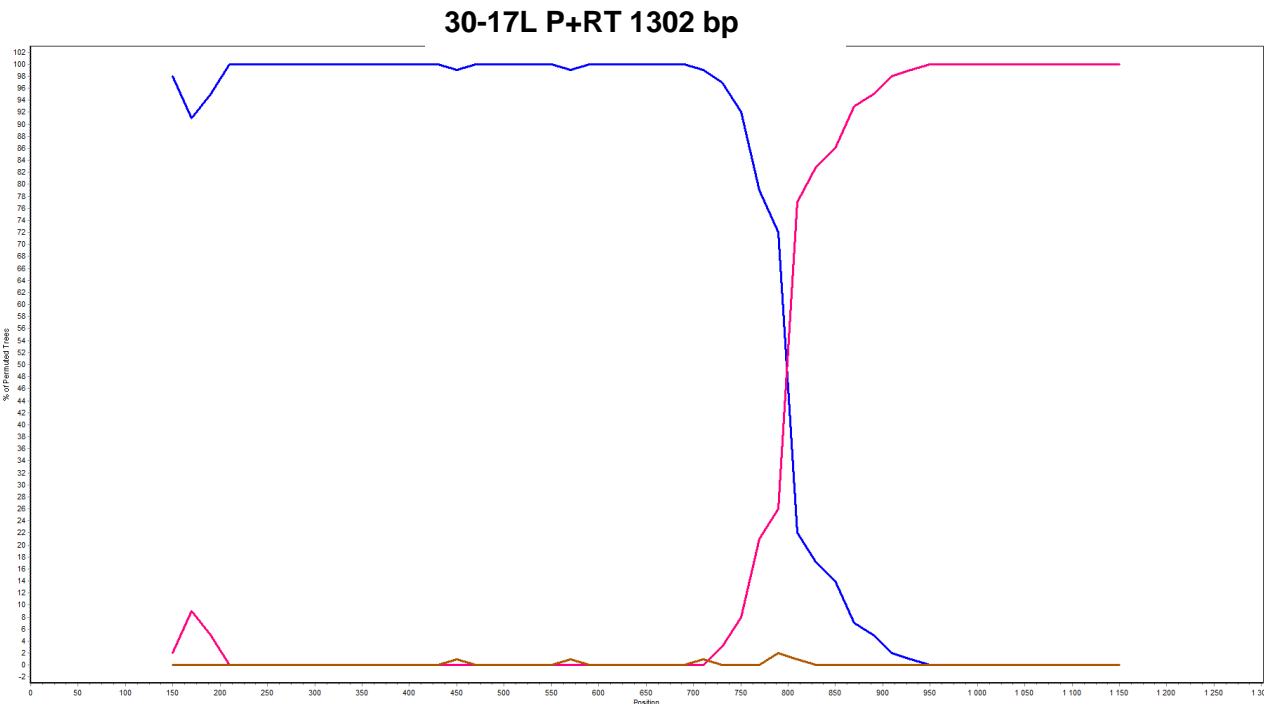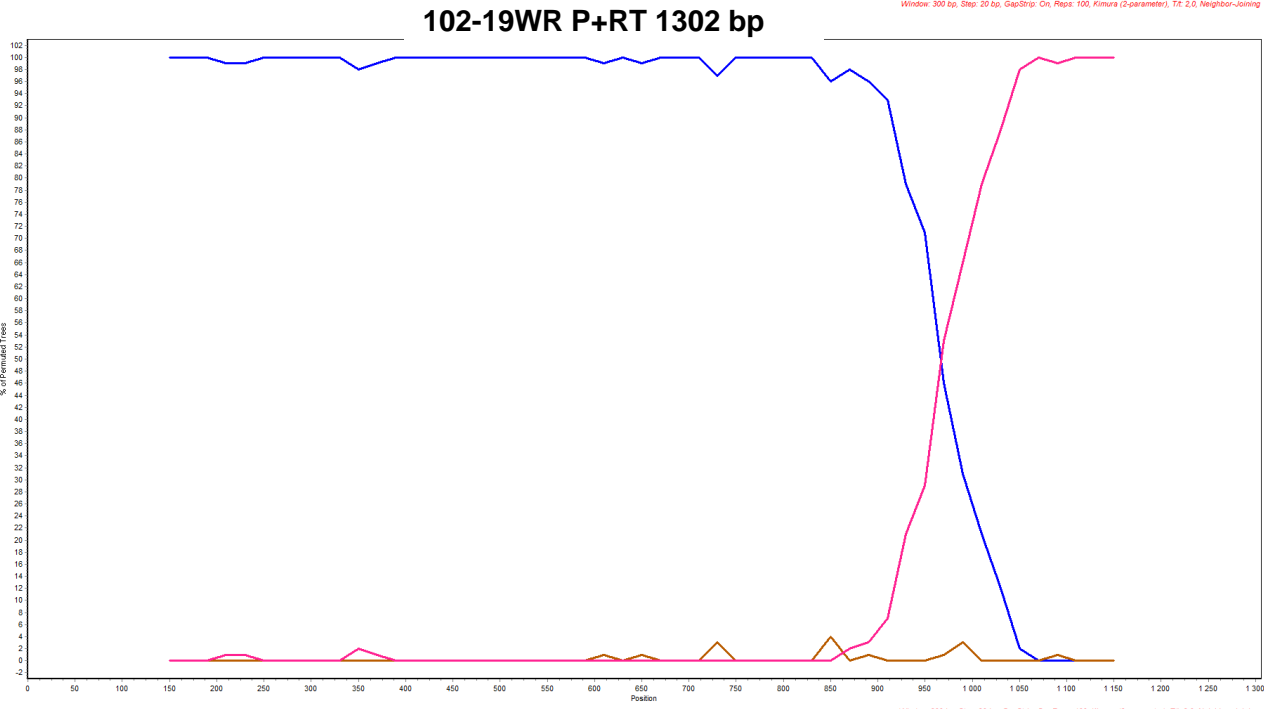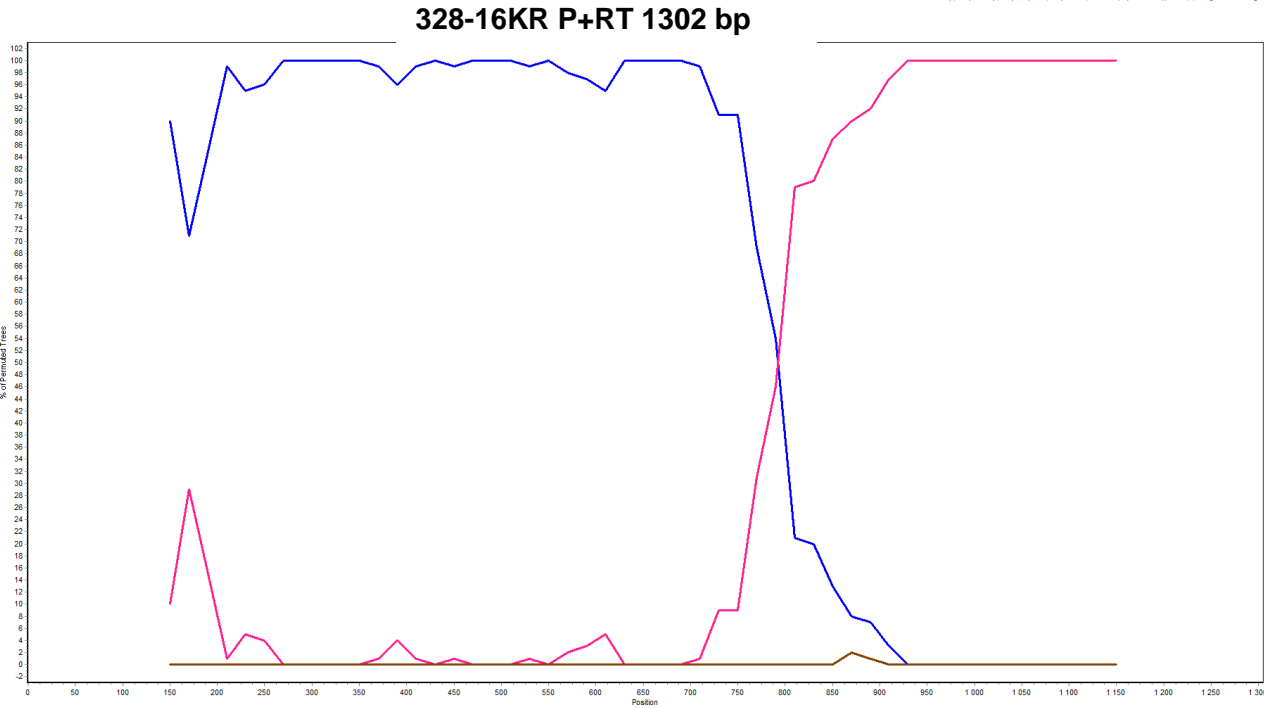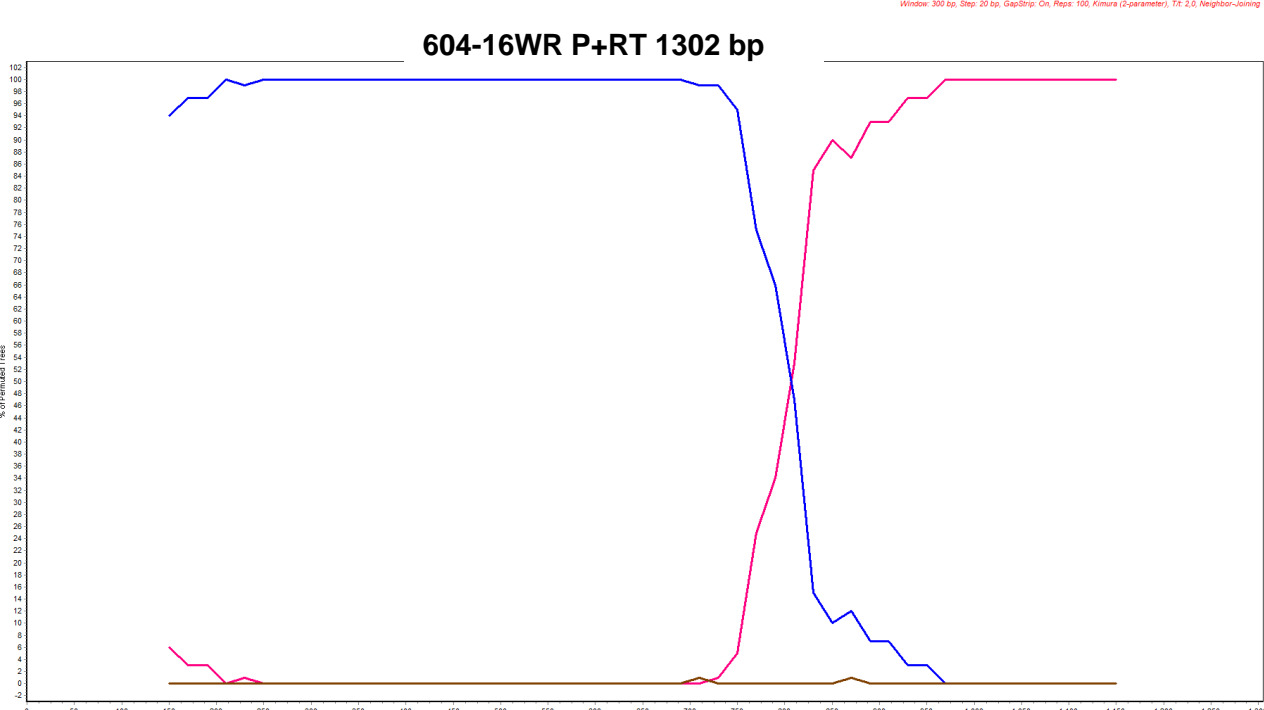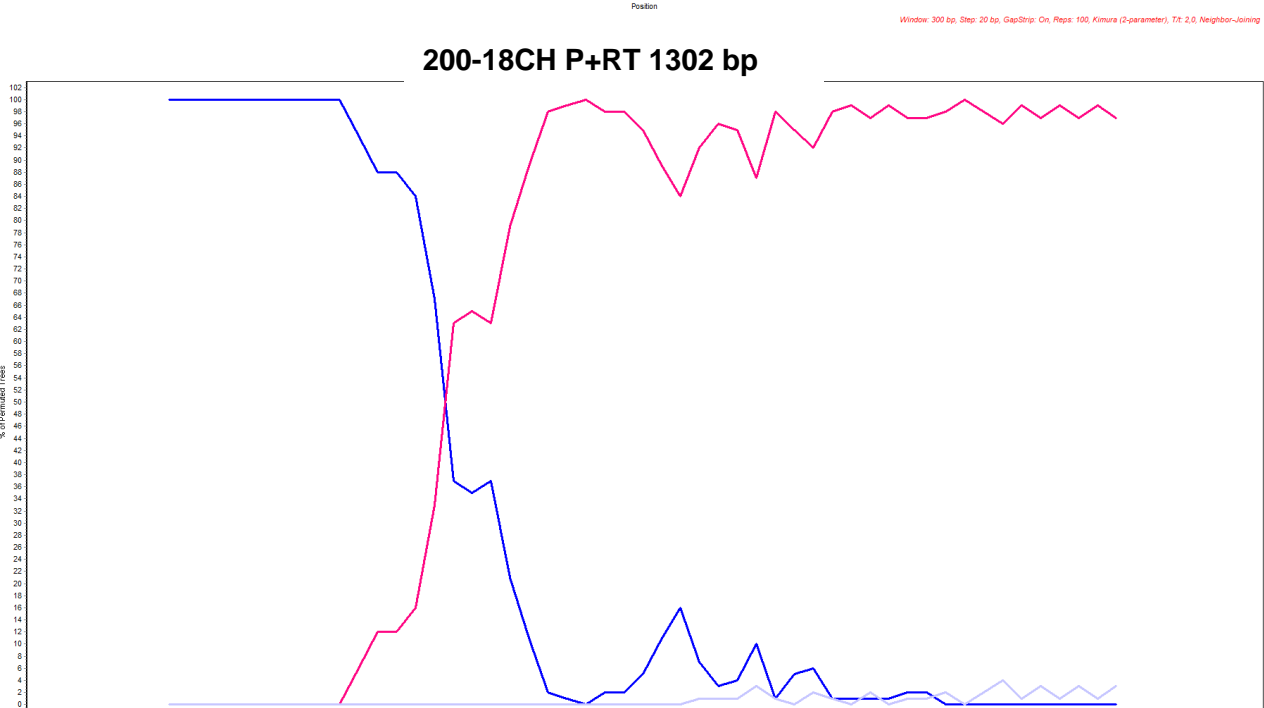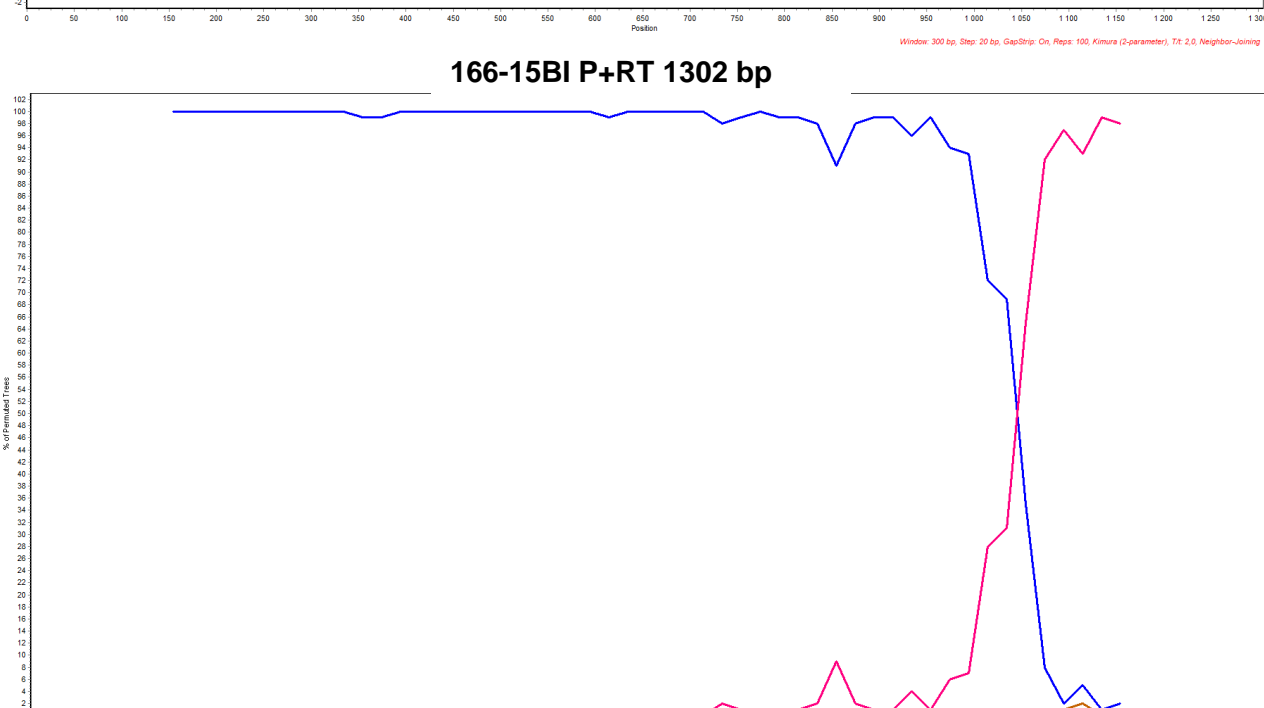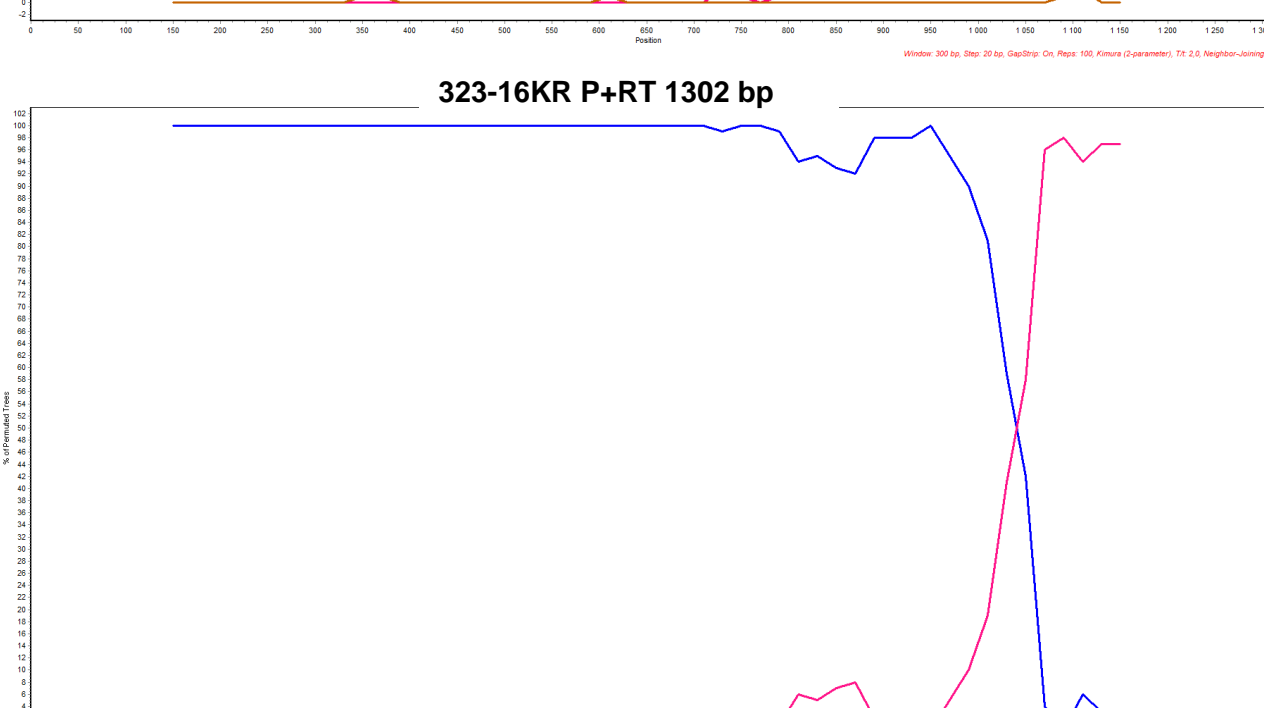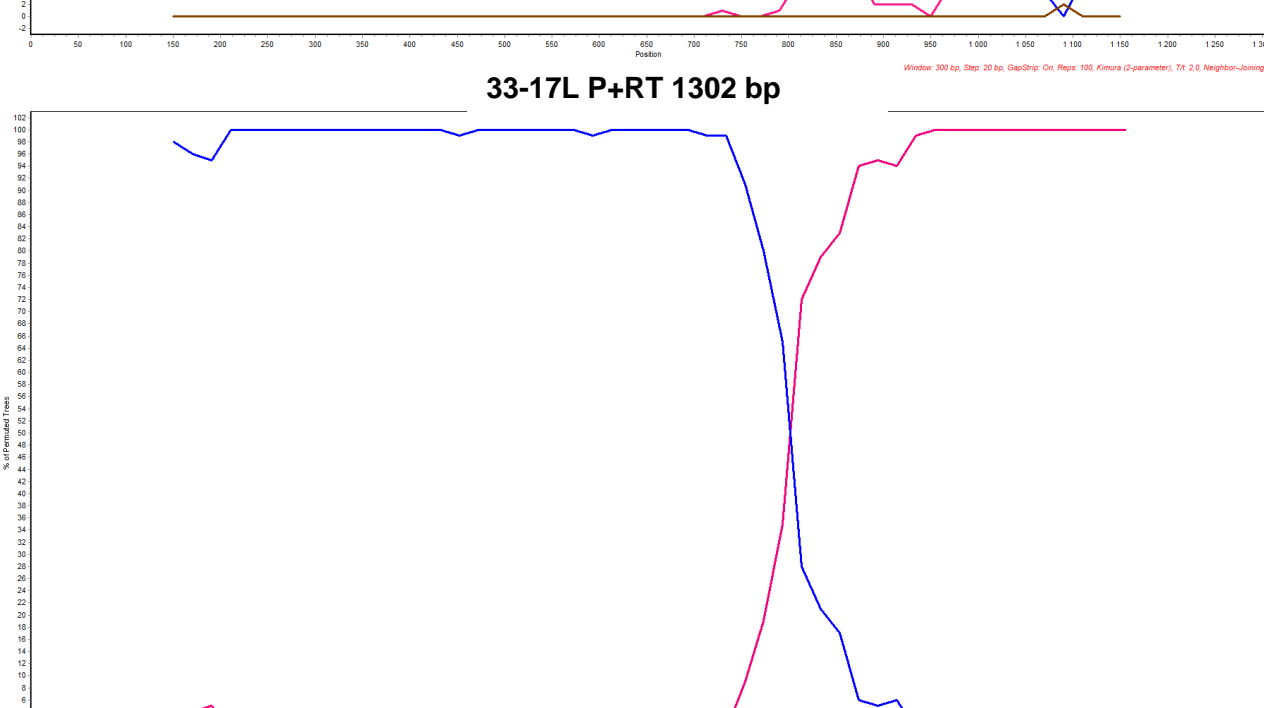

F1

C

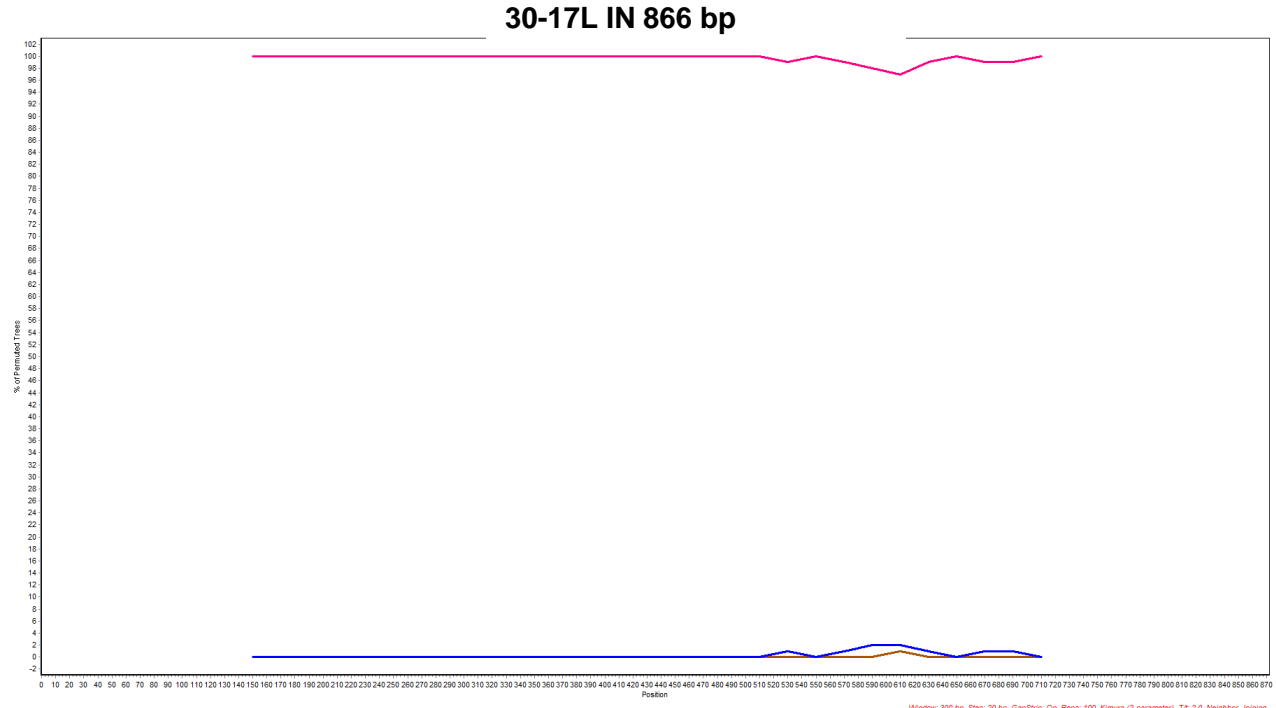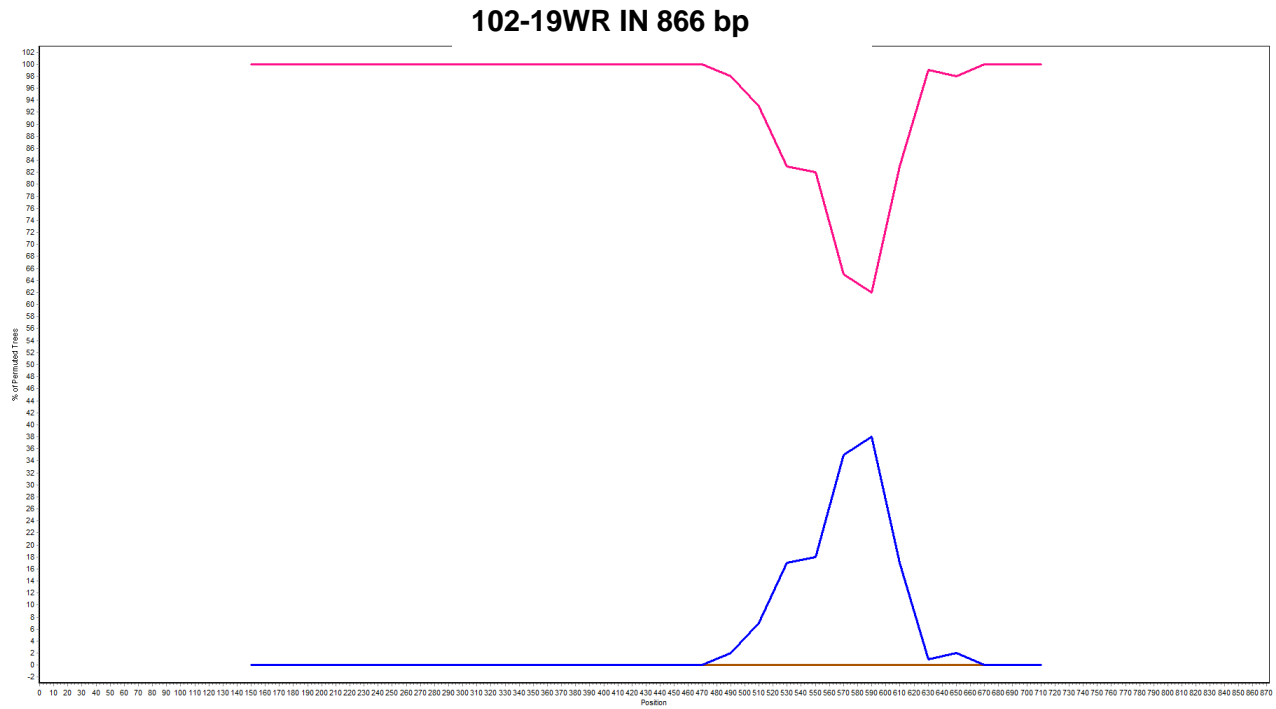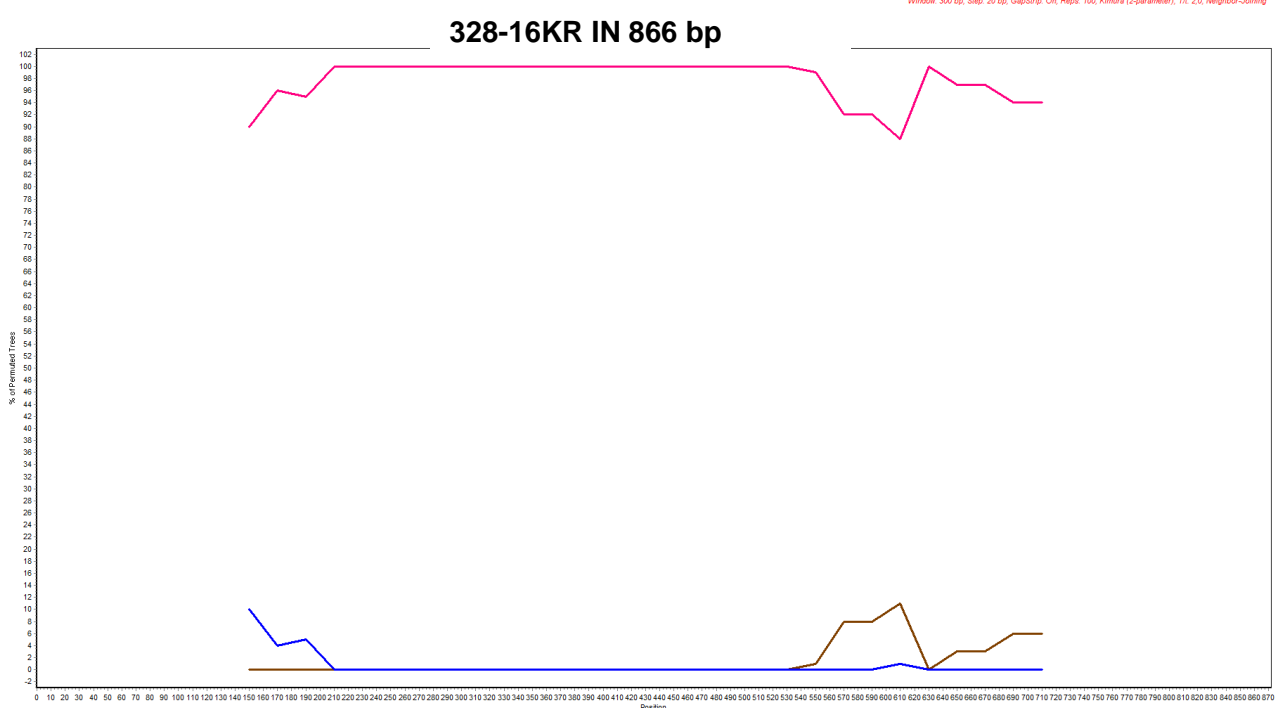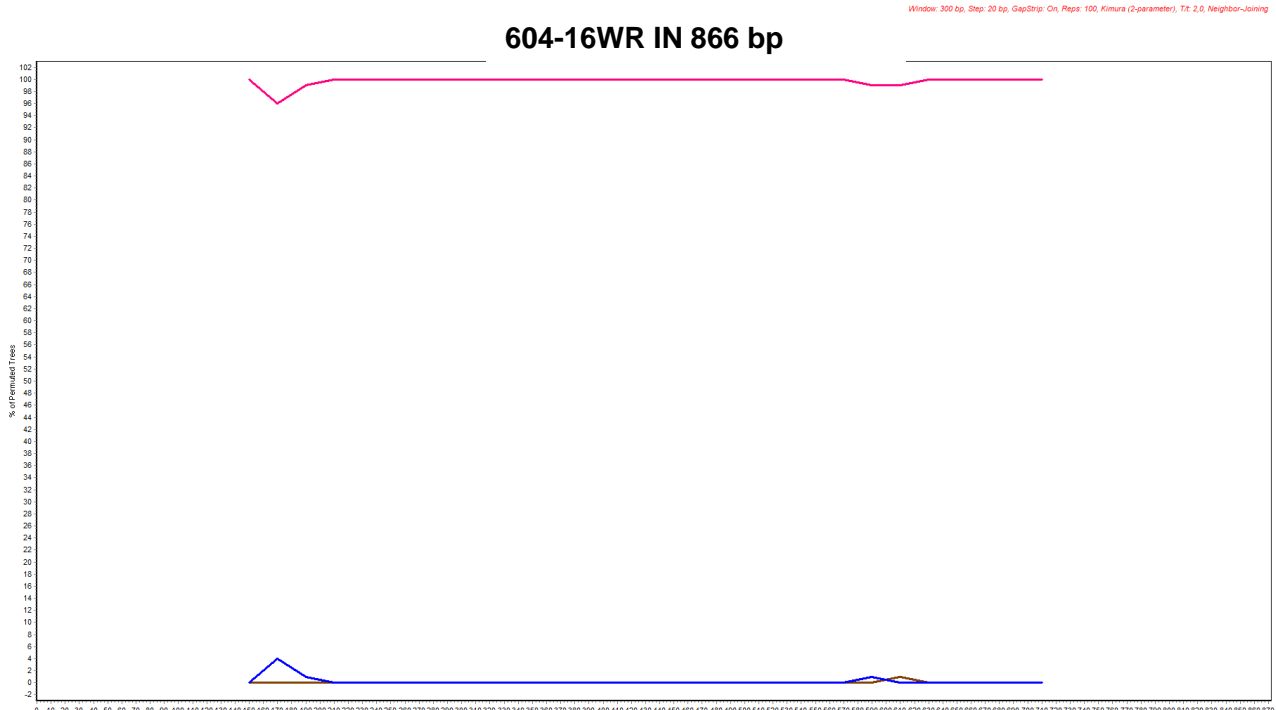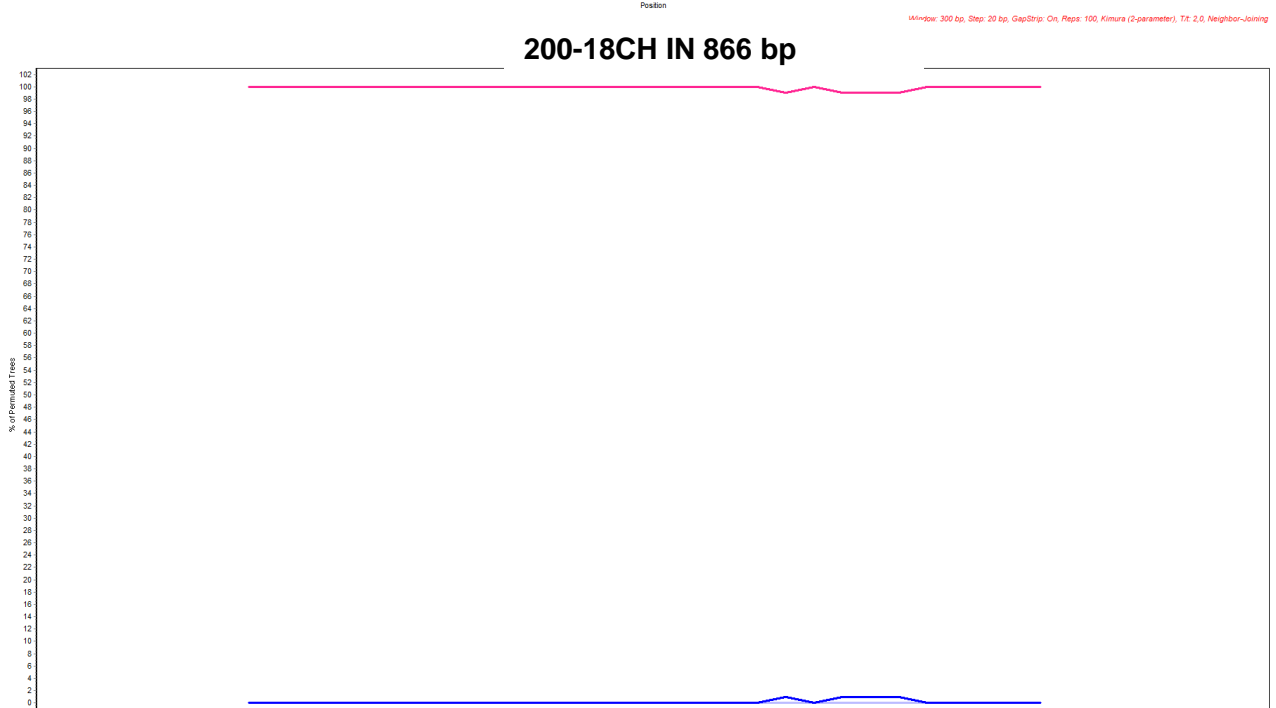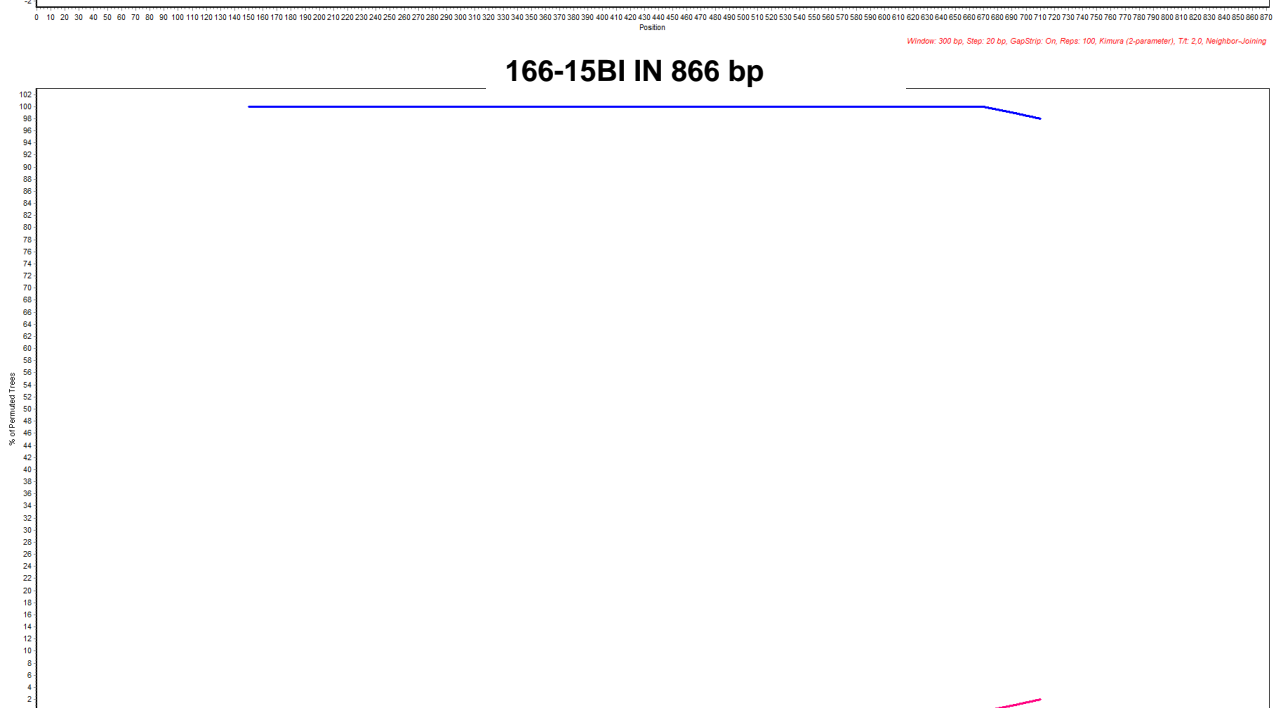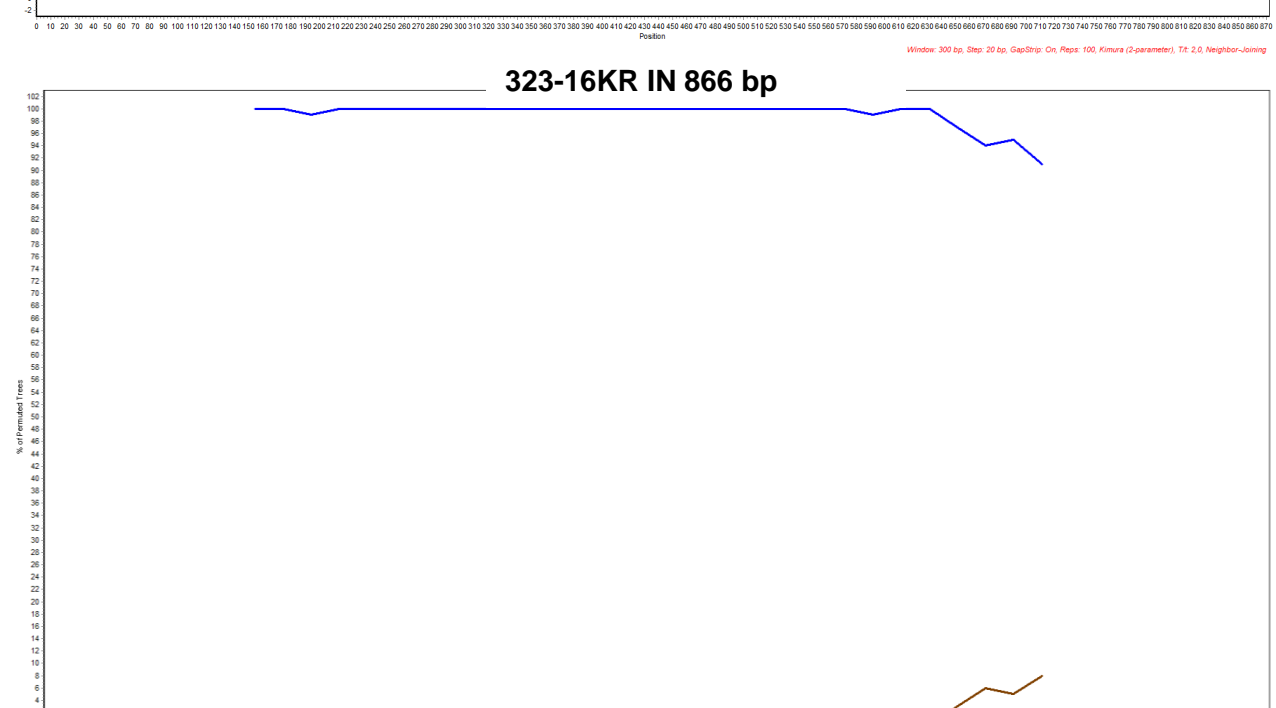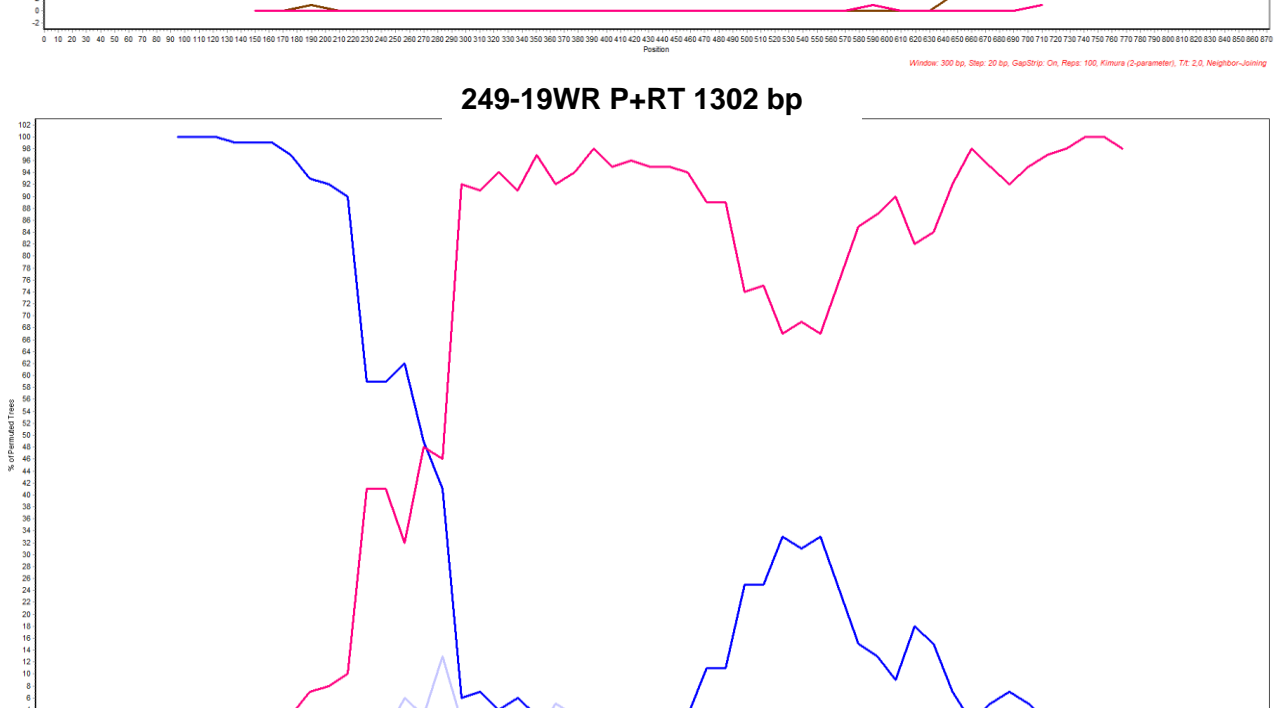

Supplement: Supplementary file 7 — Supplementary Information 7. [file 41598_2021_96125_MOESM7_ESM.pdf]

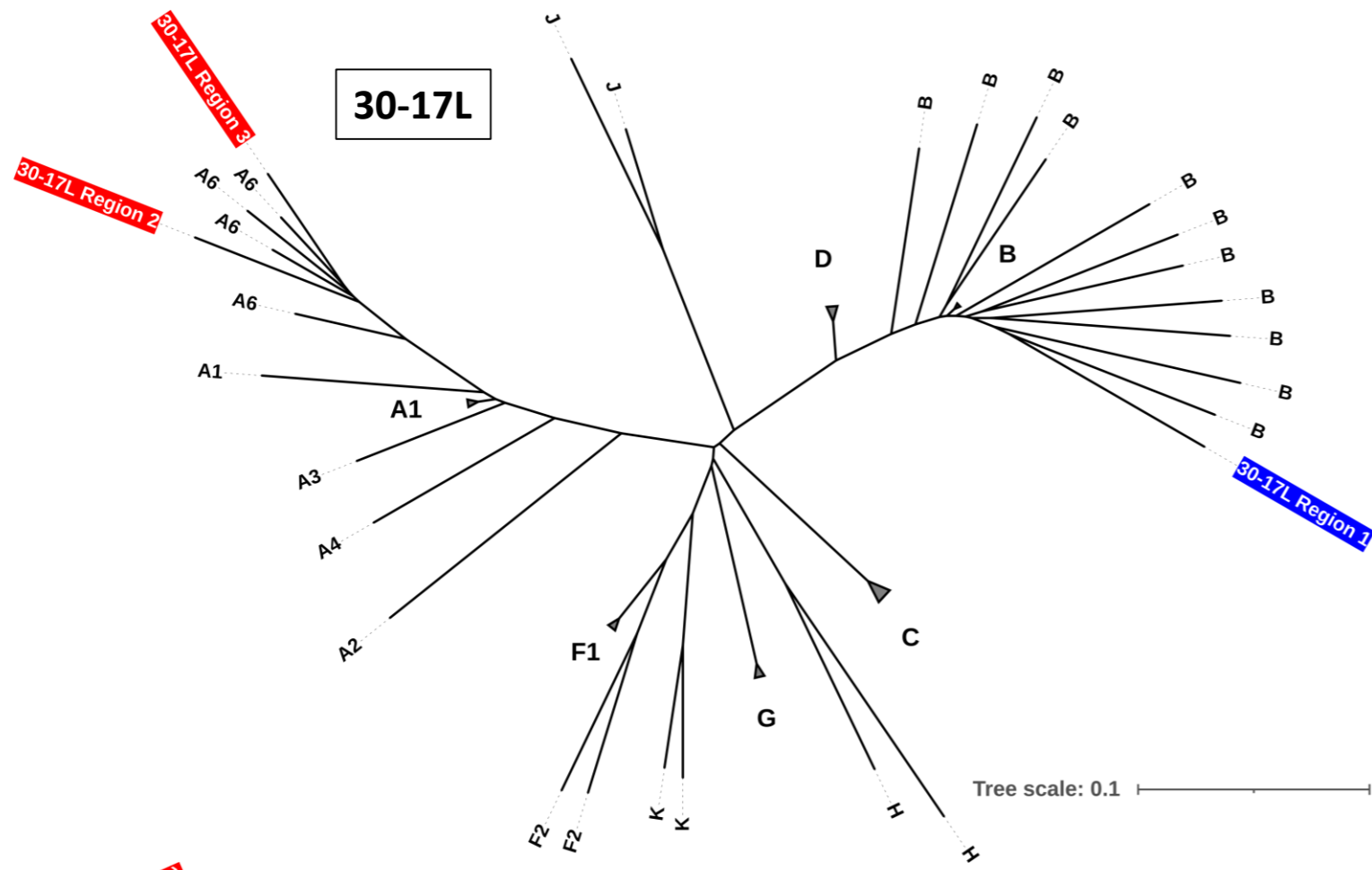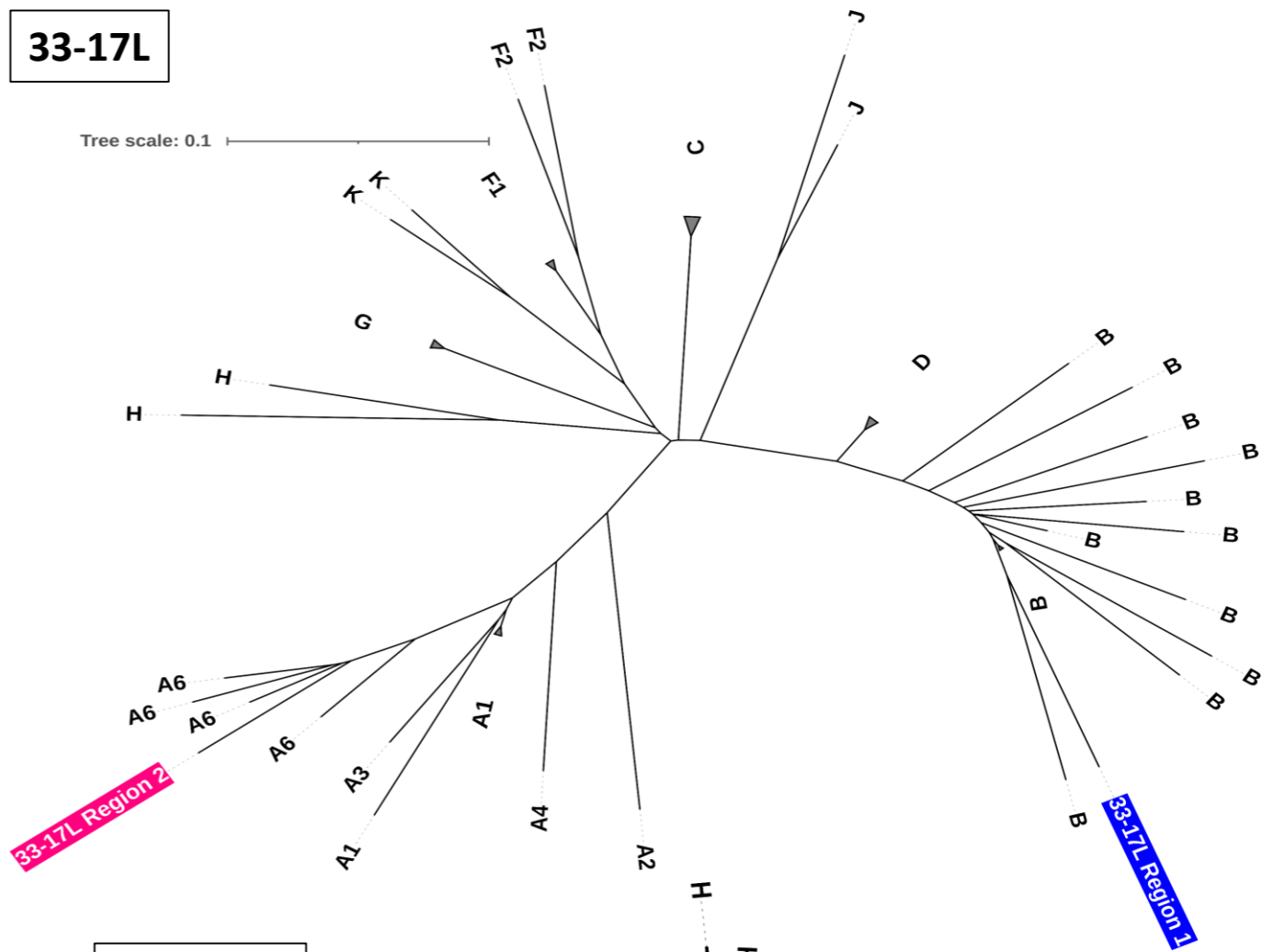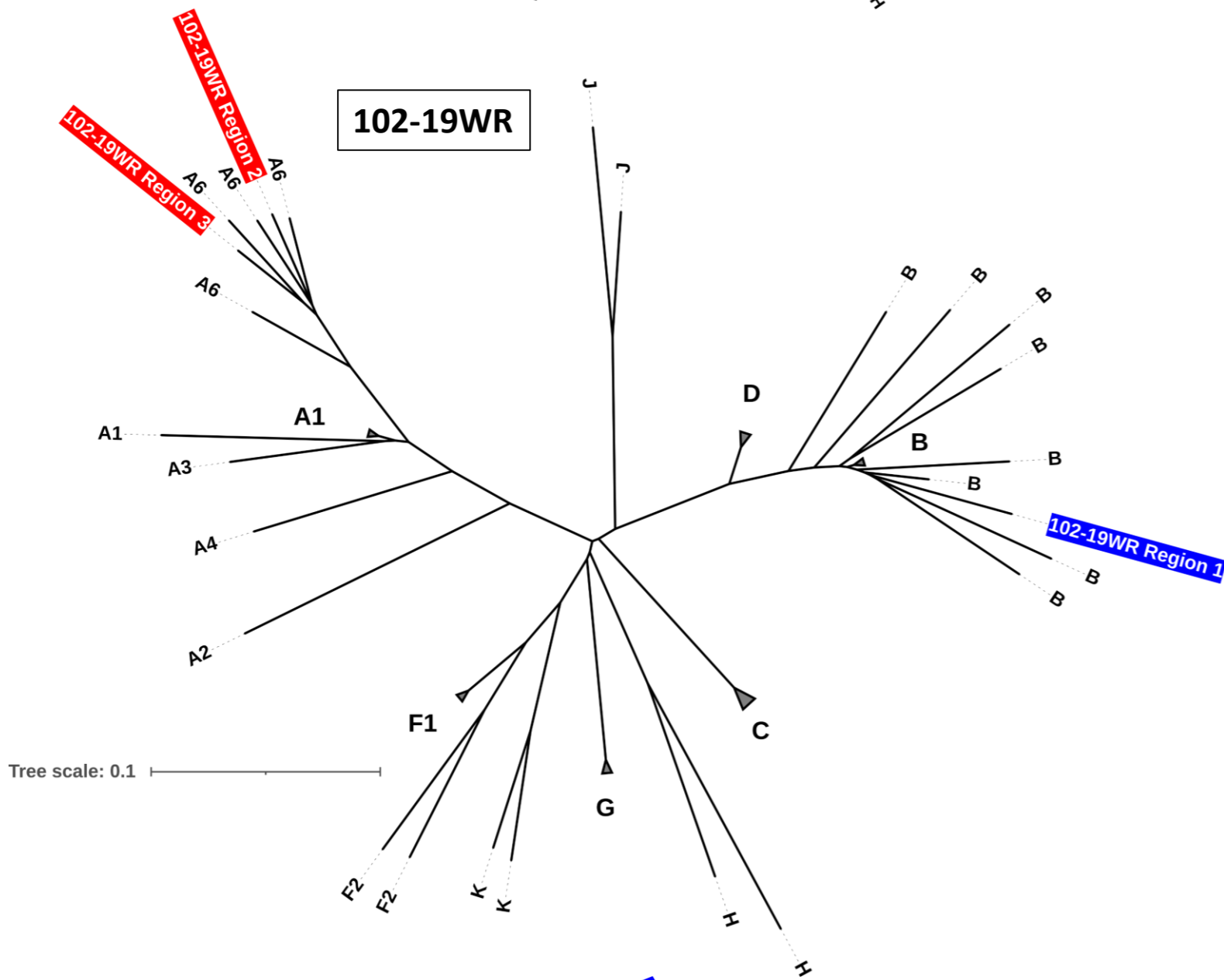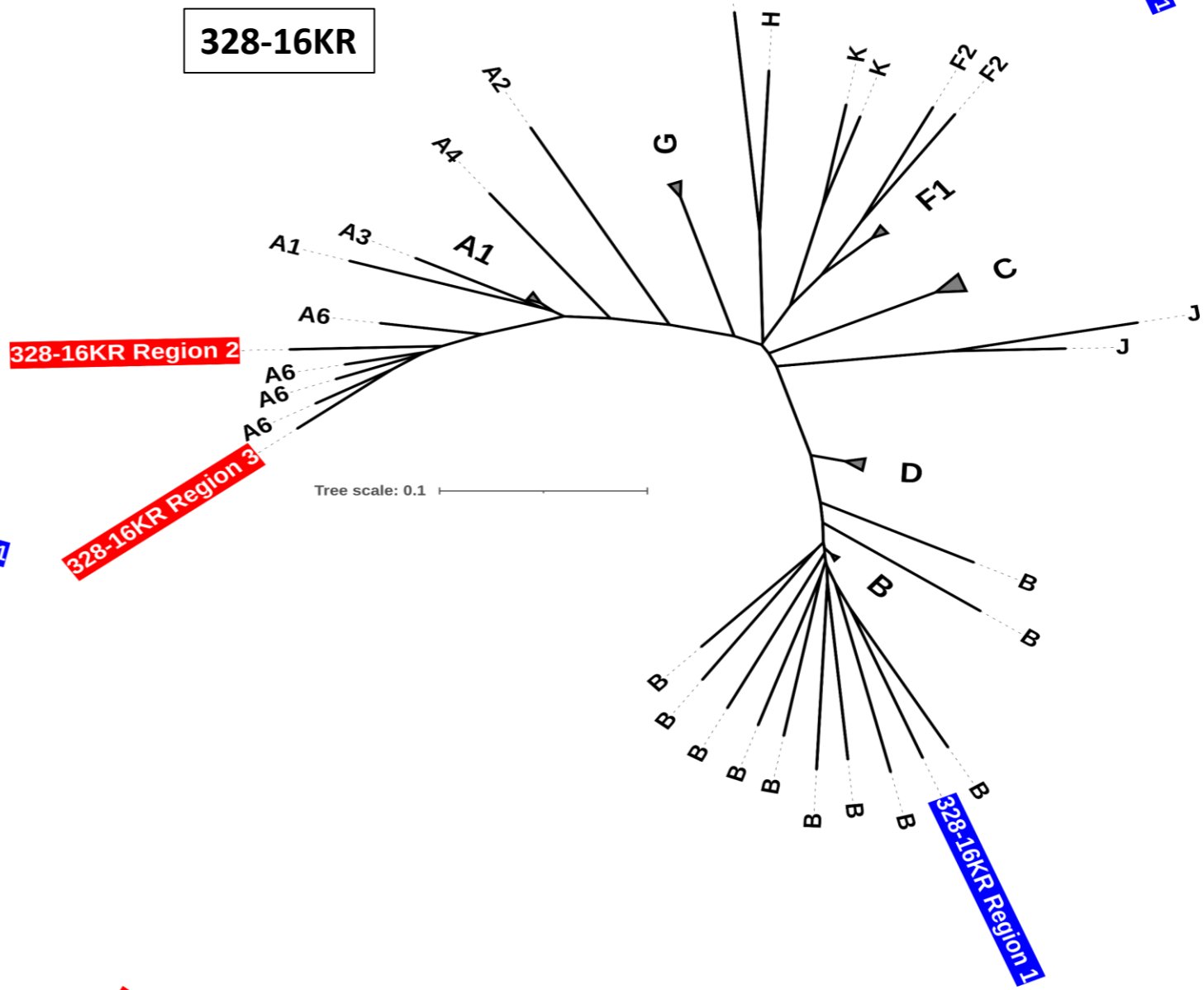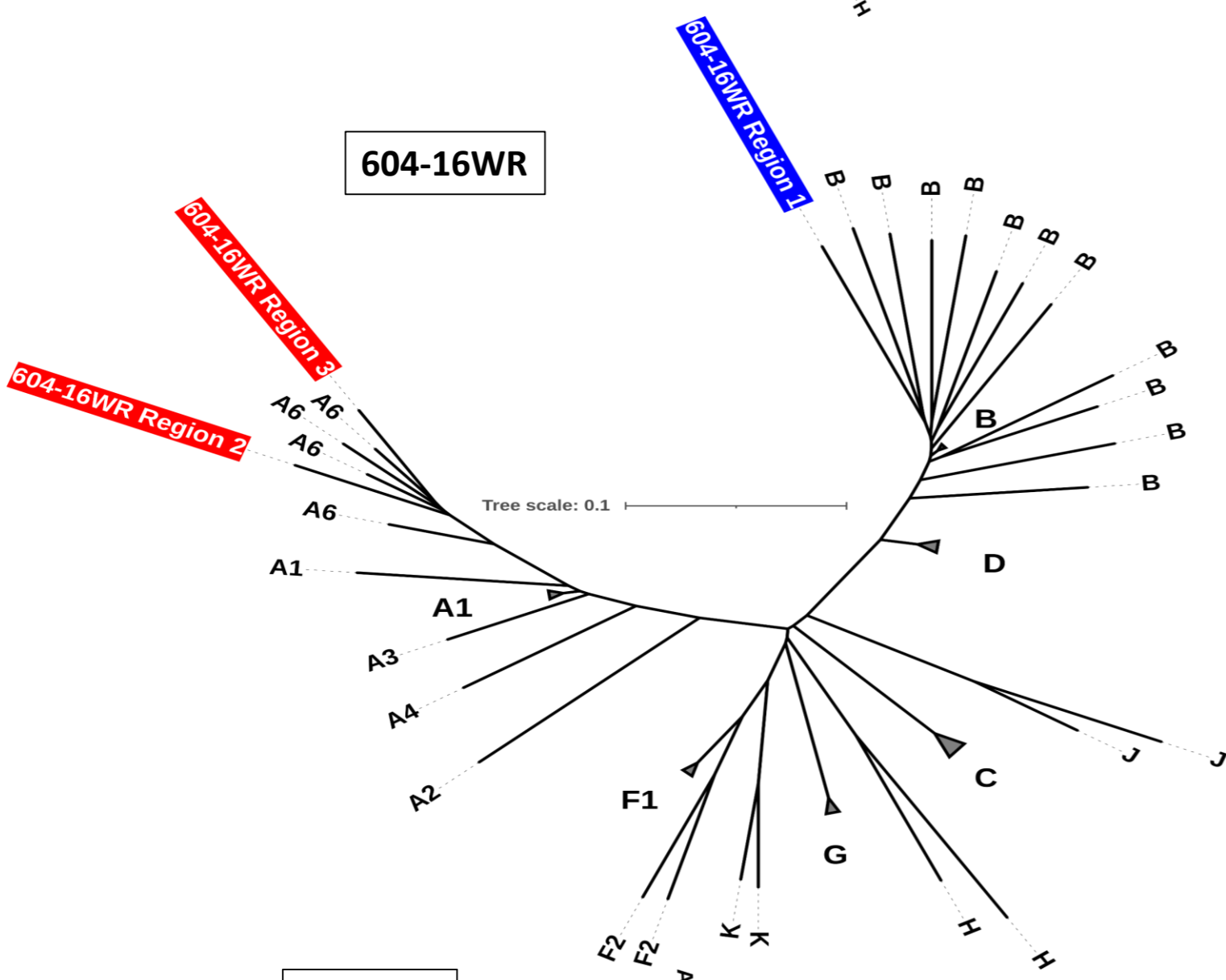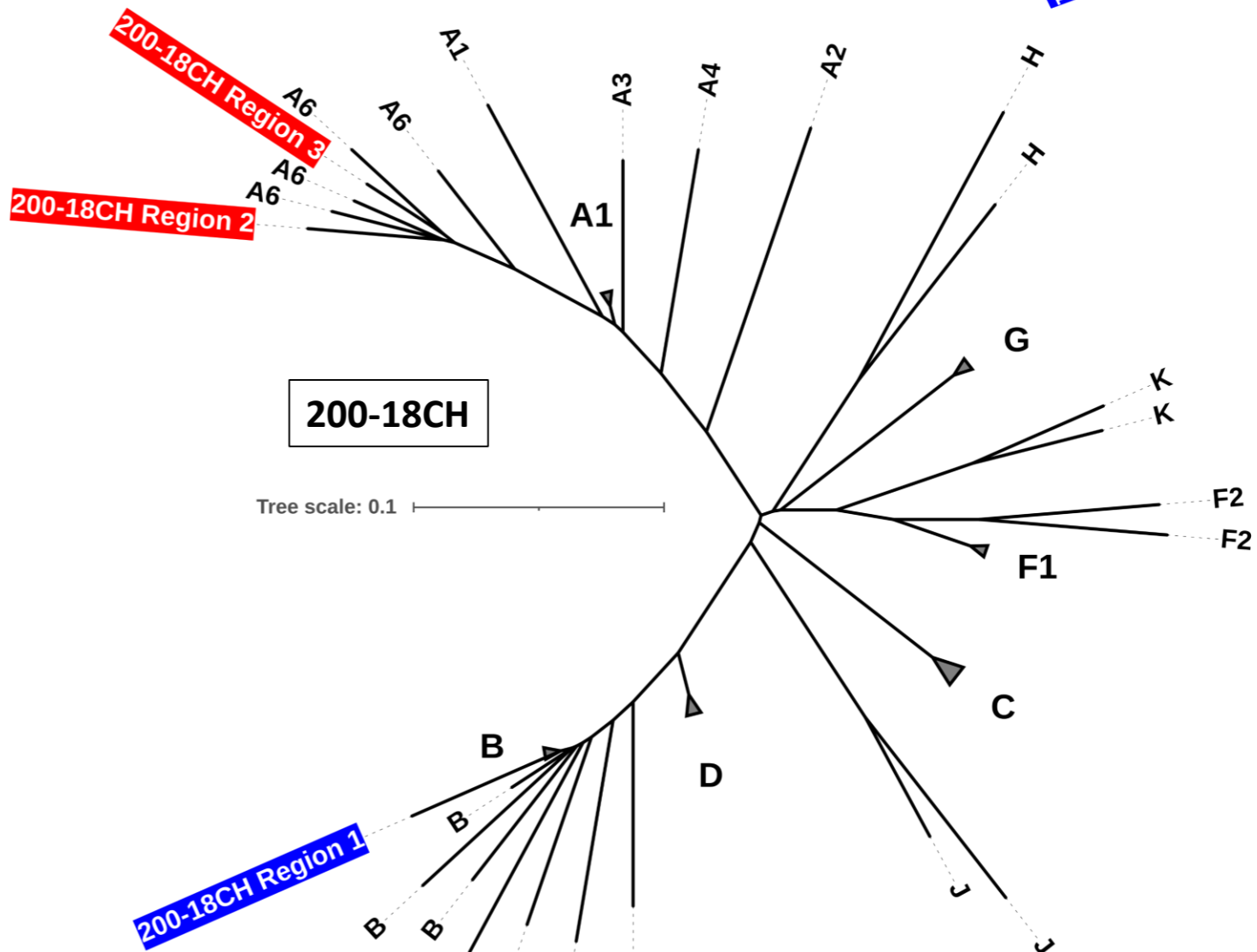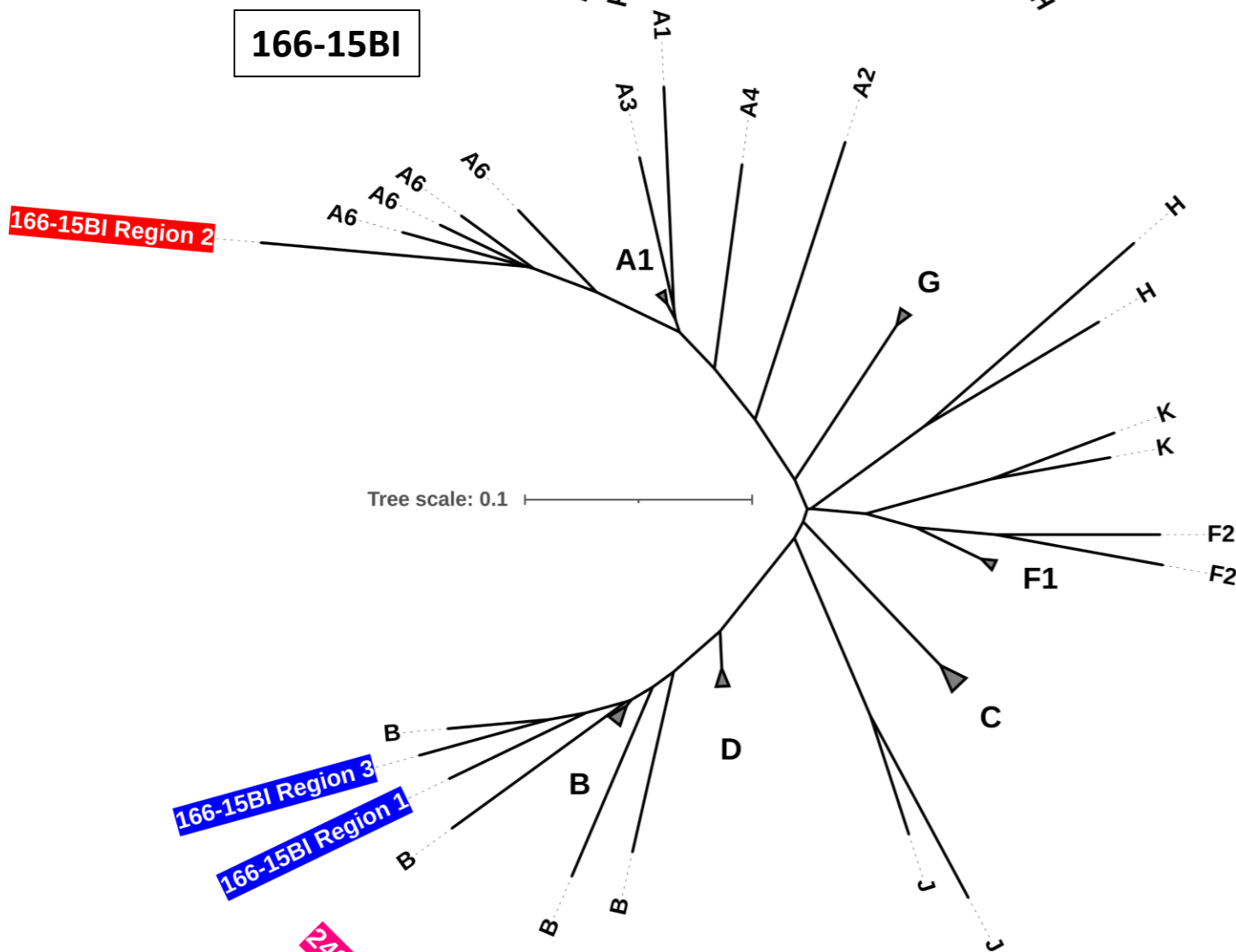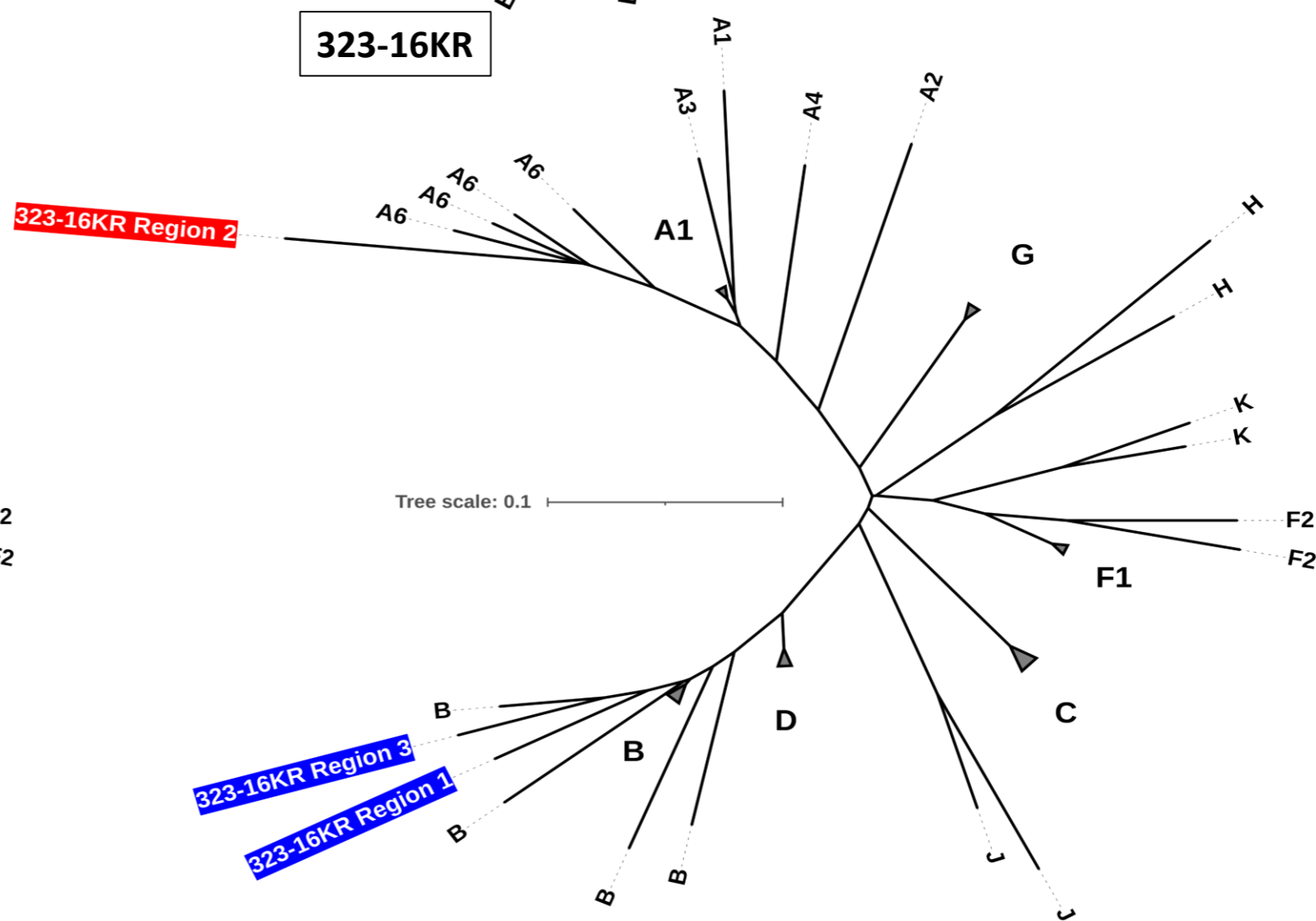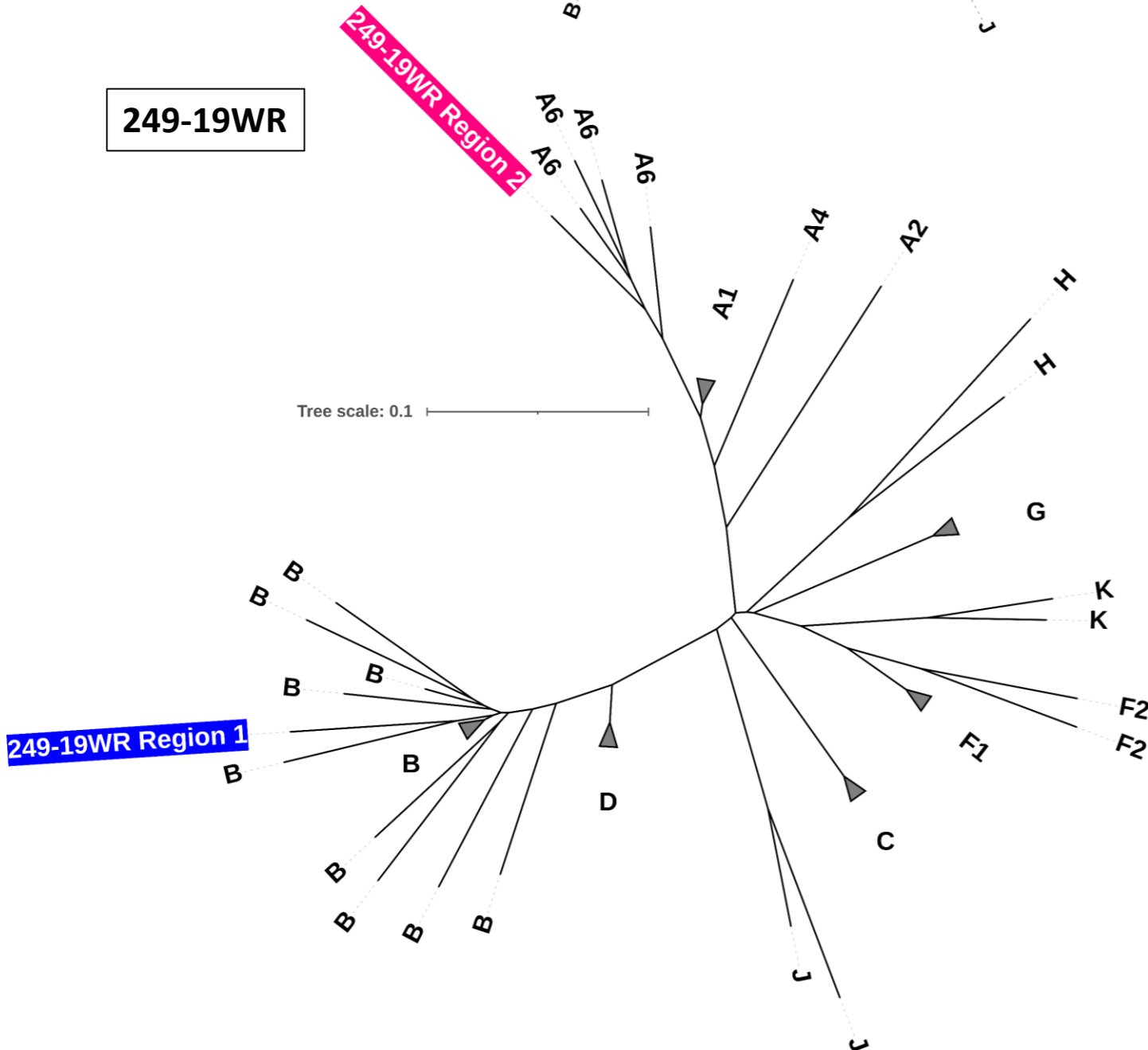

Supplement: Supplementary file 8 — Supplementary Information 8. [file 41598_2021_96125_MOESM8_ESM.pdf]

A1

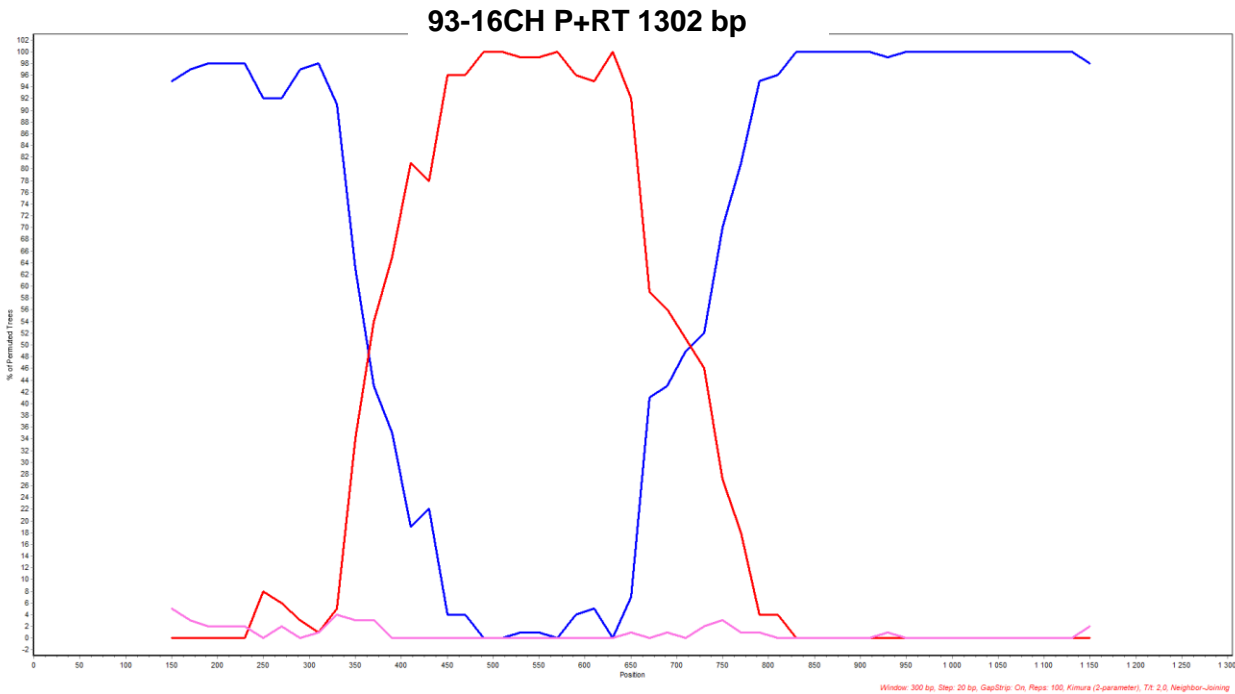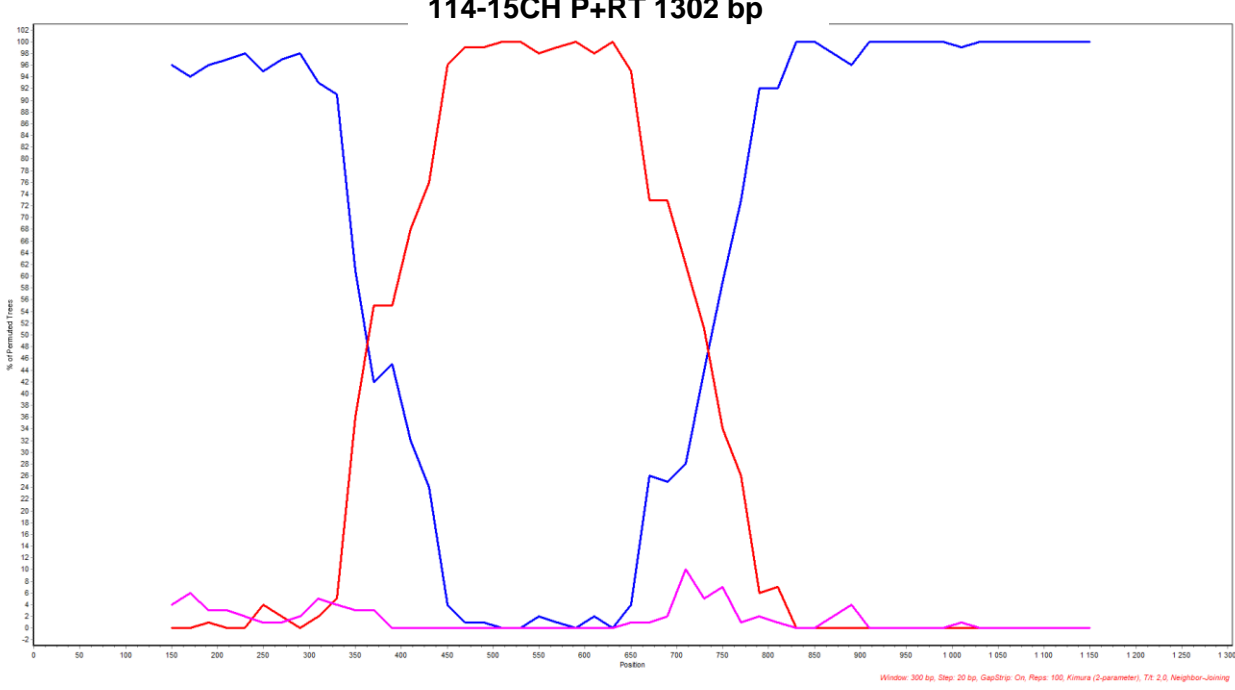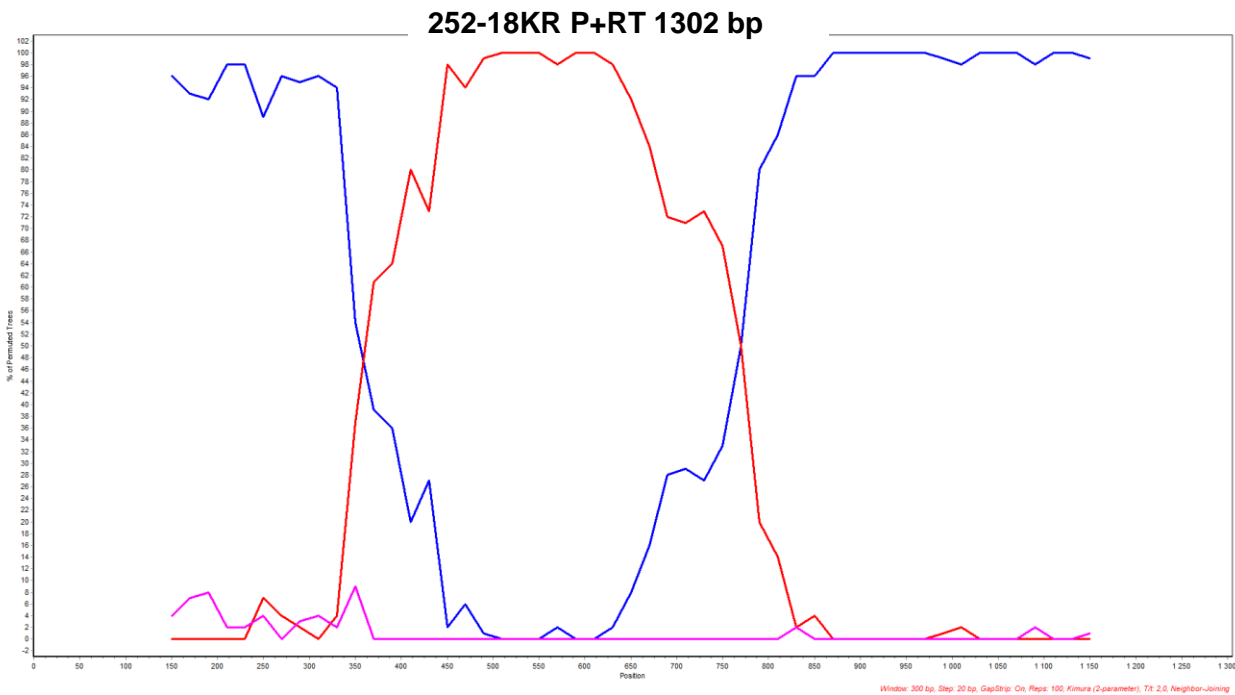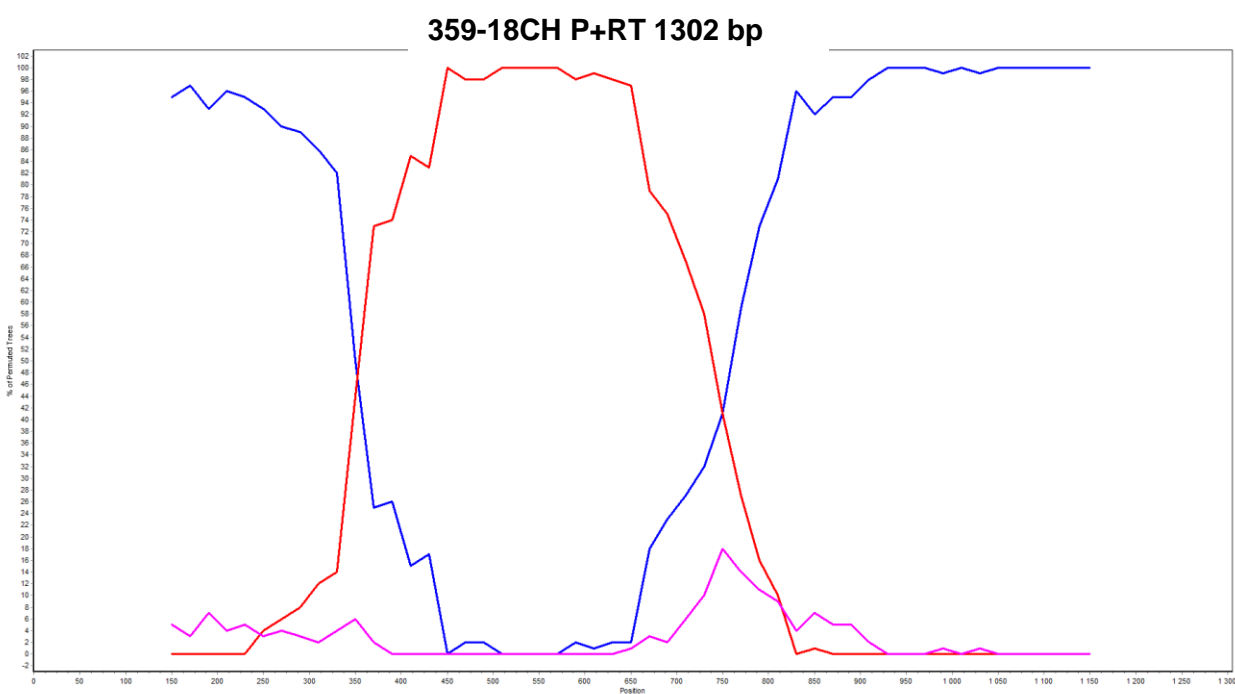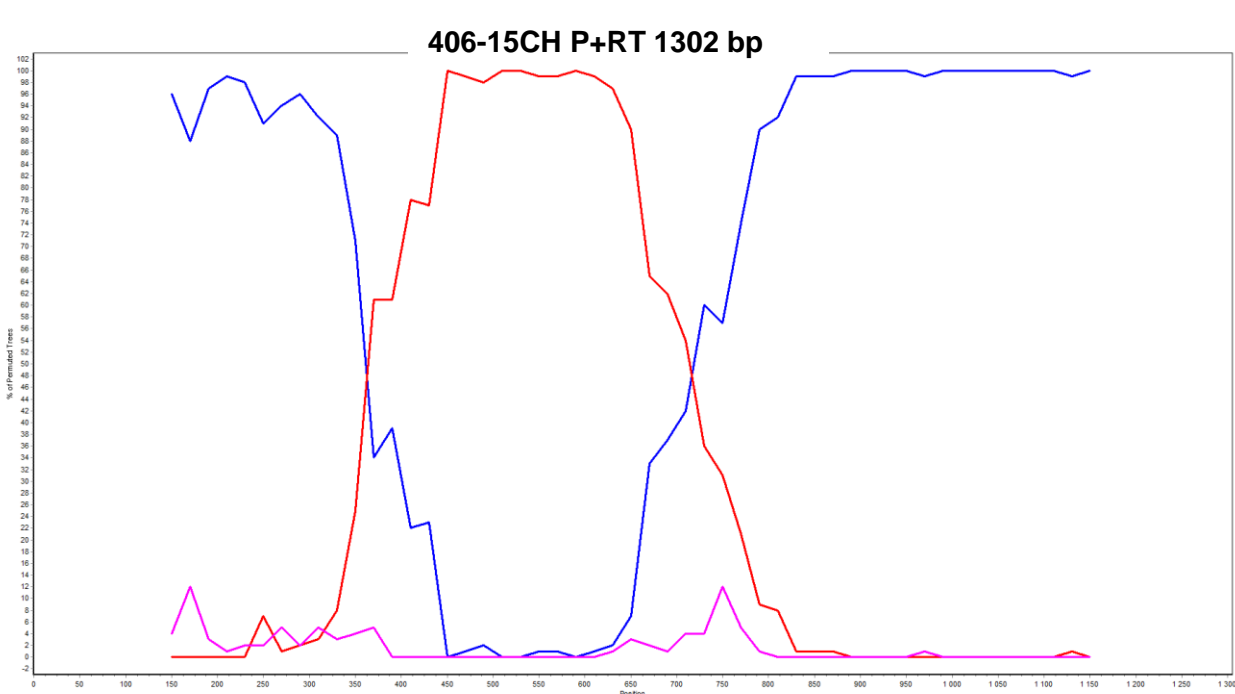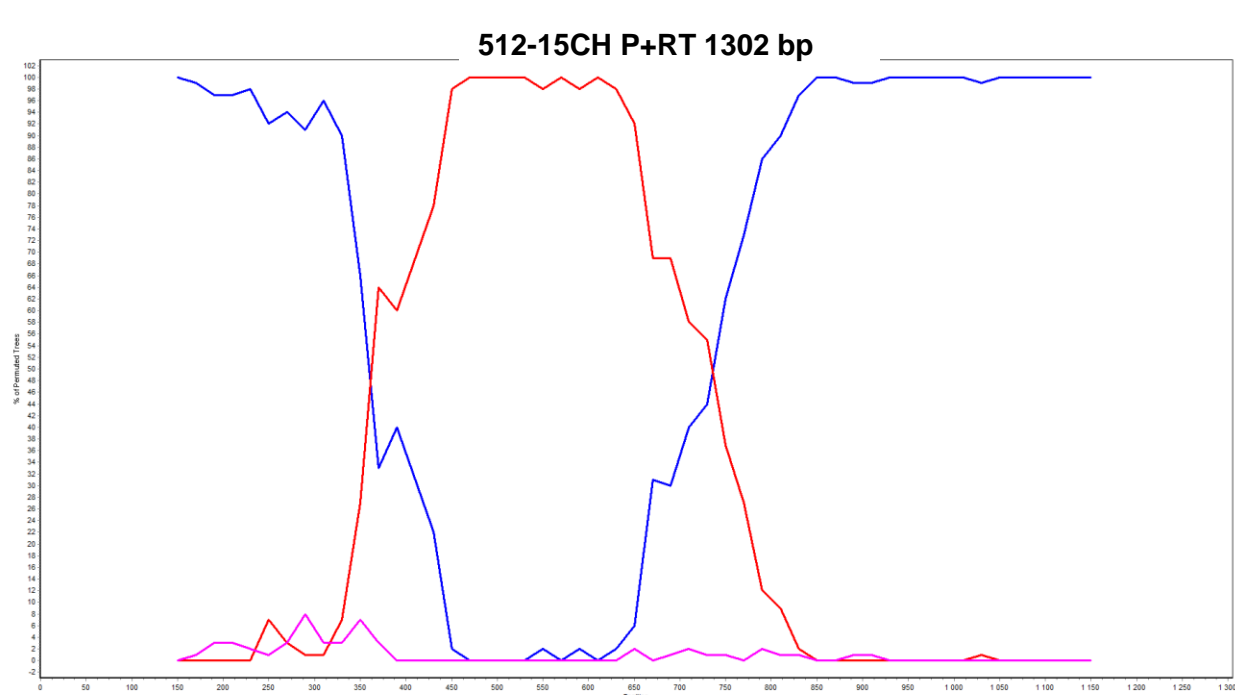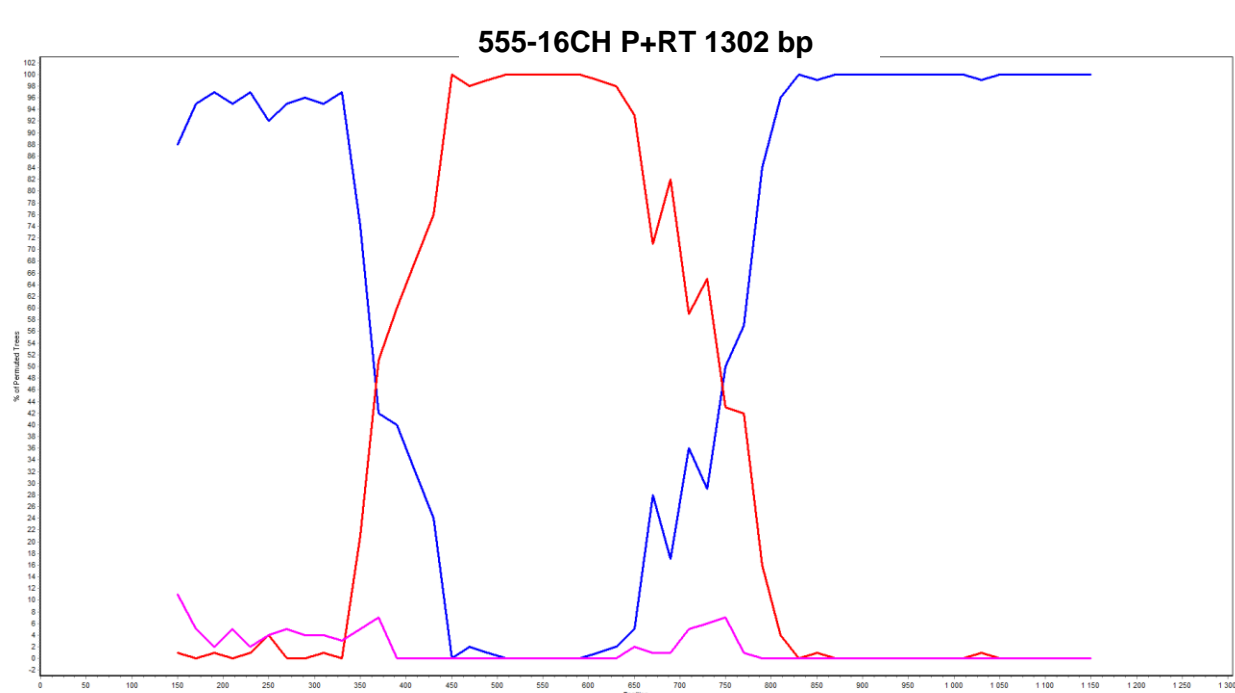

B

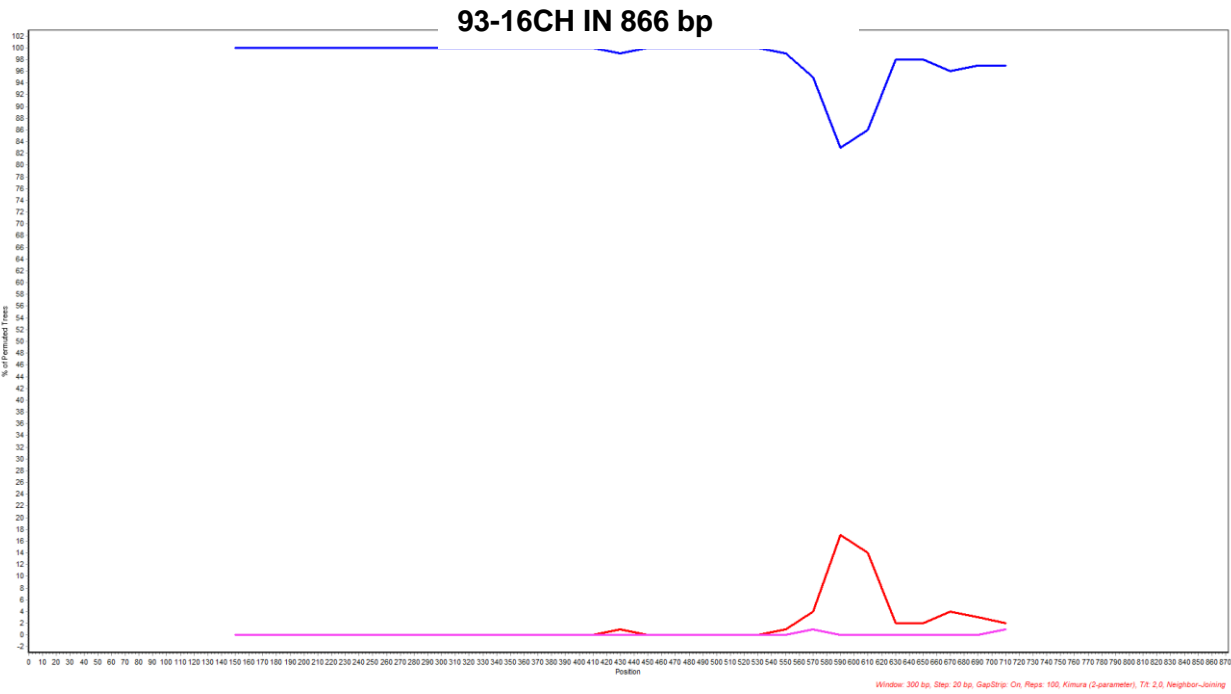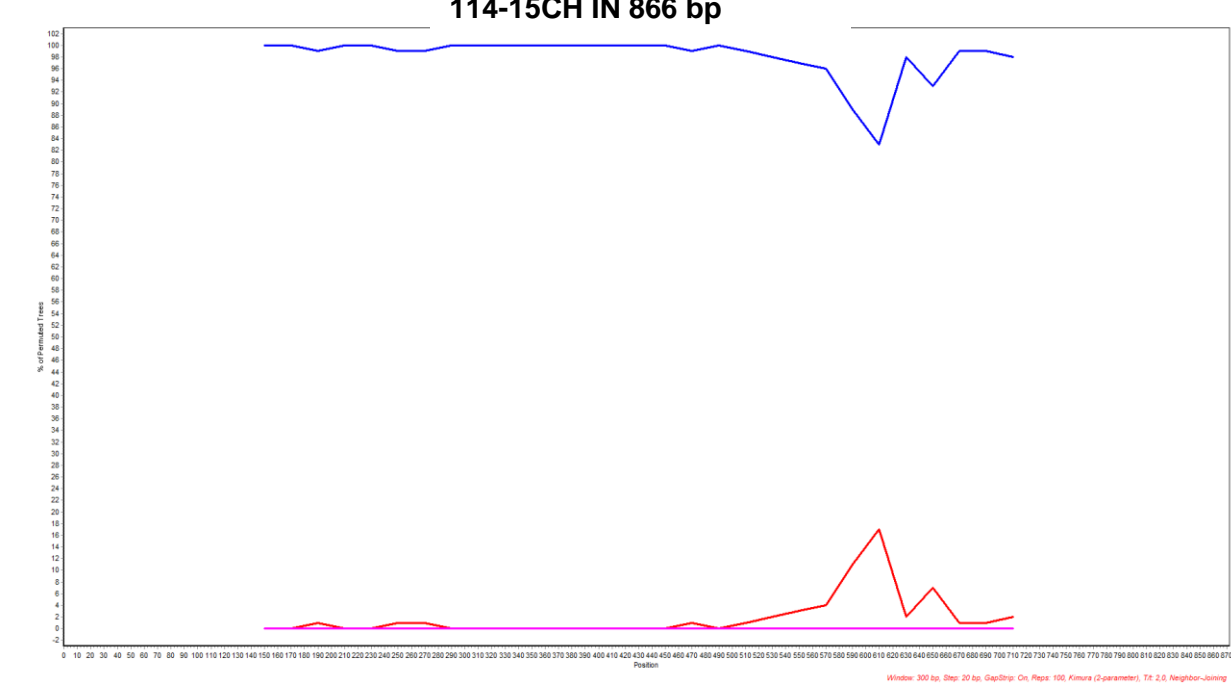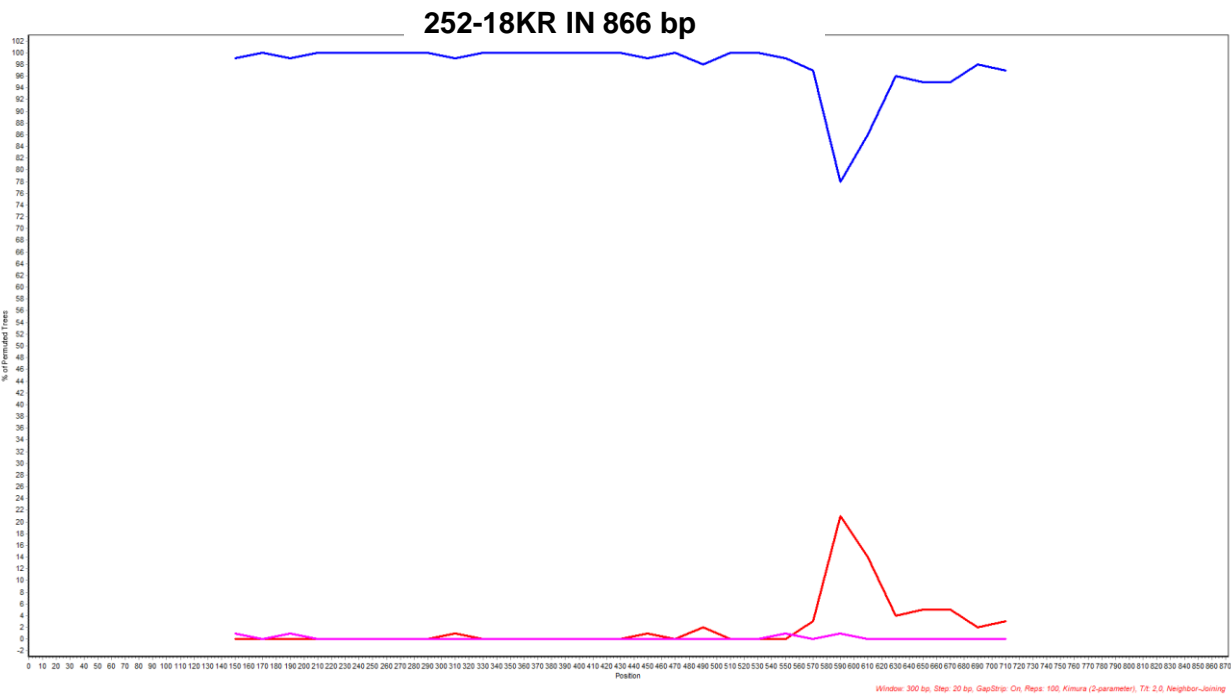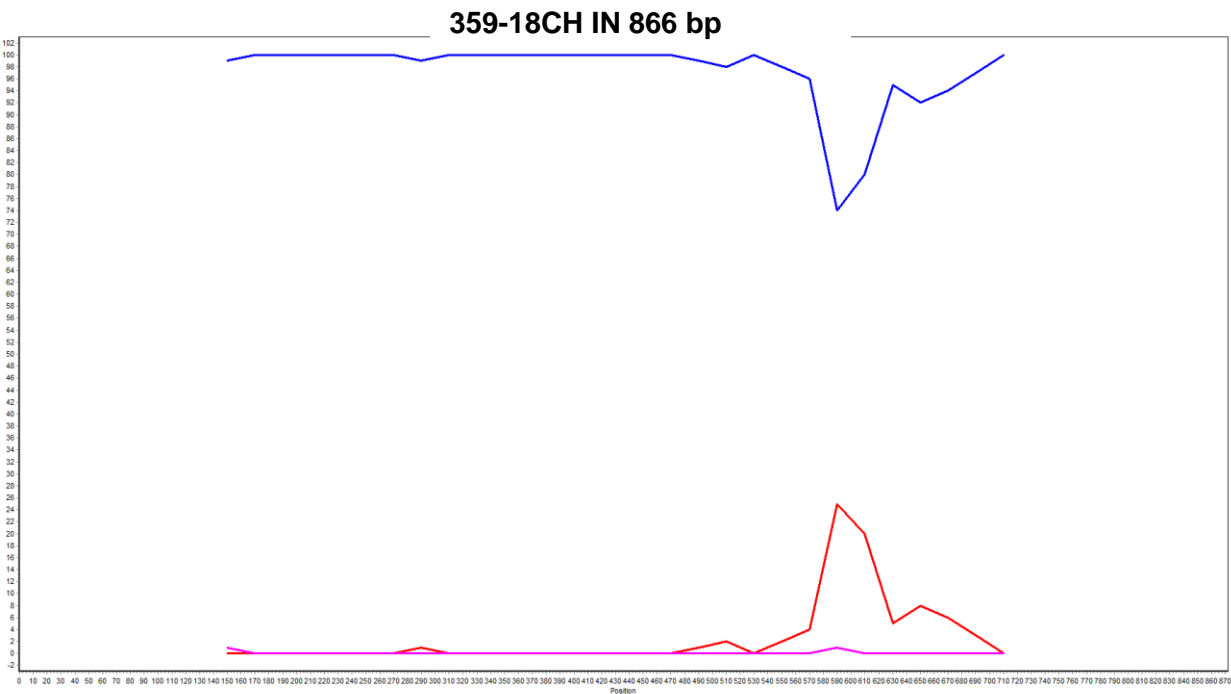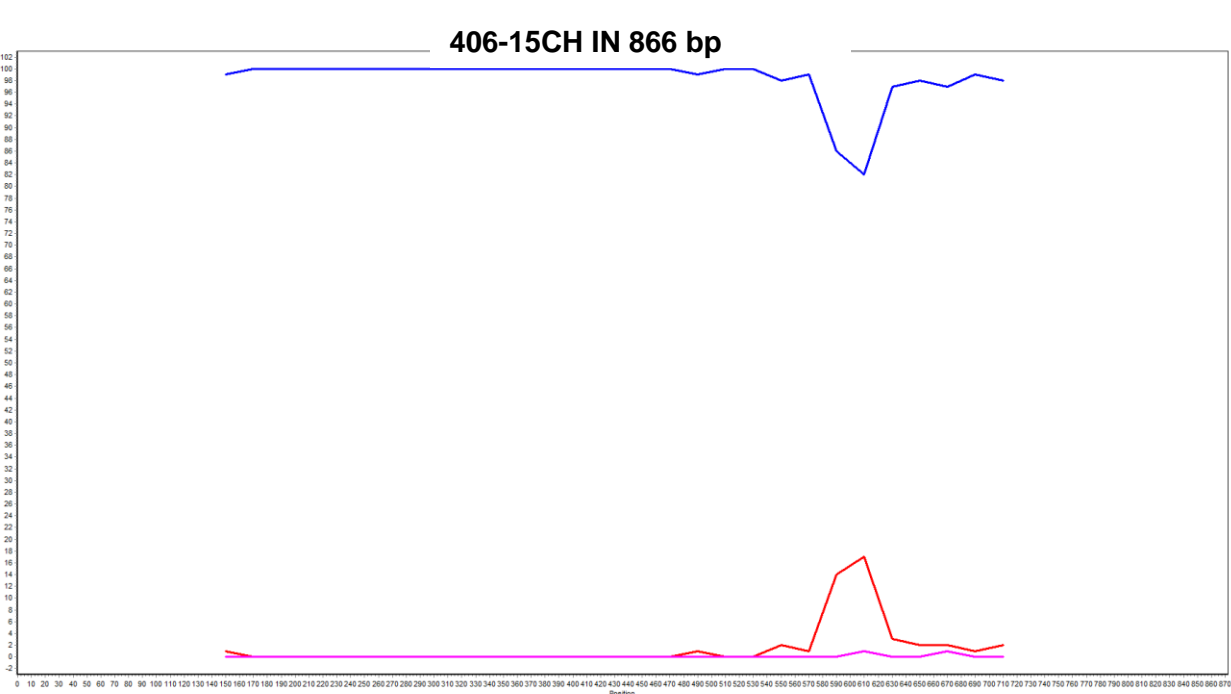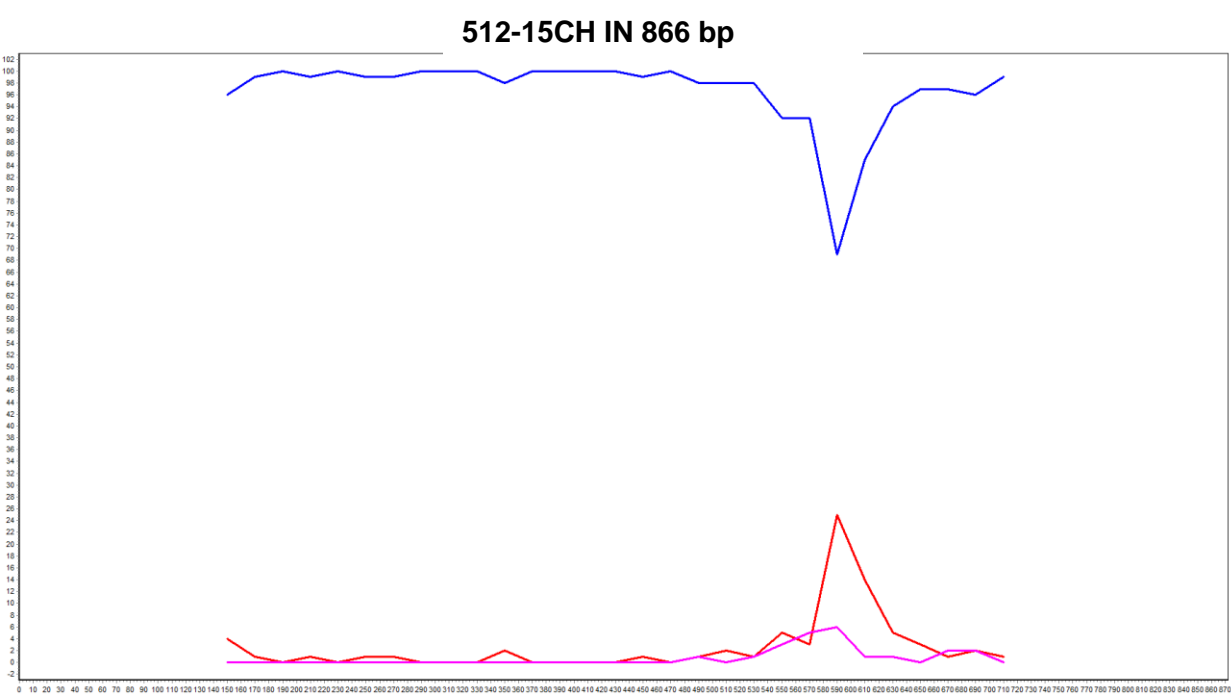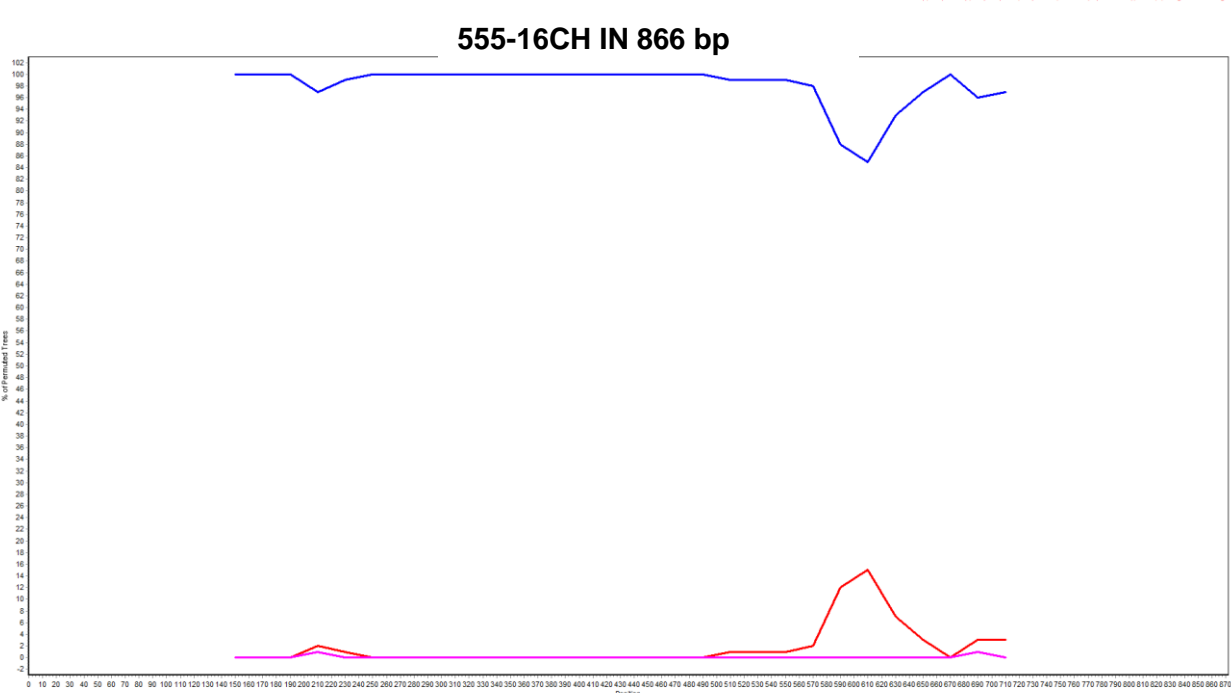

K

Supplement: Supplementary file 9 — Supplementary Information 9. [file 41598_2021_96125_MOESM9_ESM.pdf]

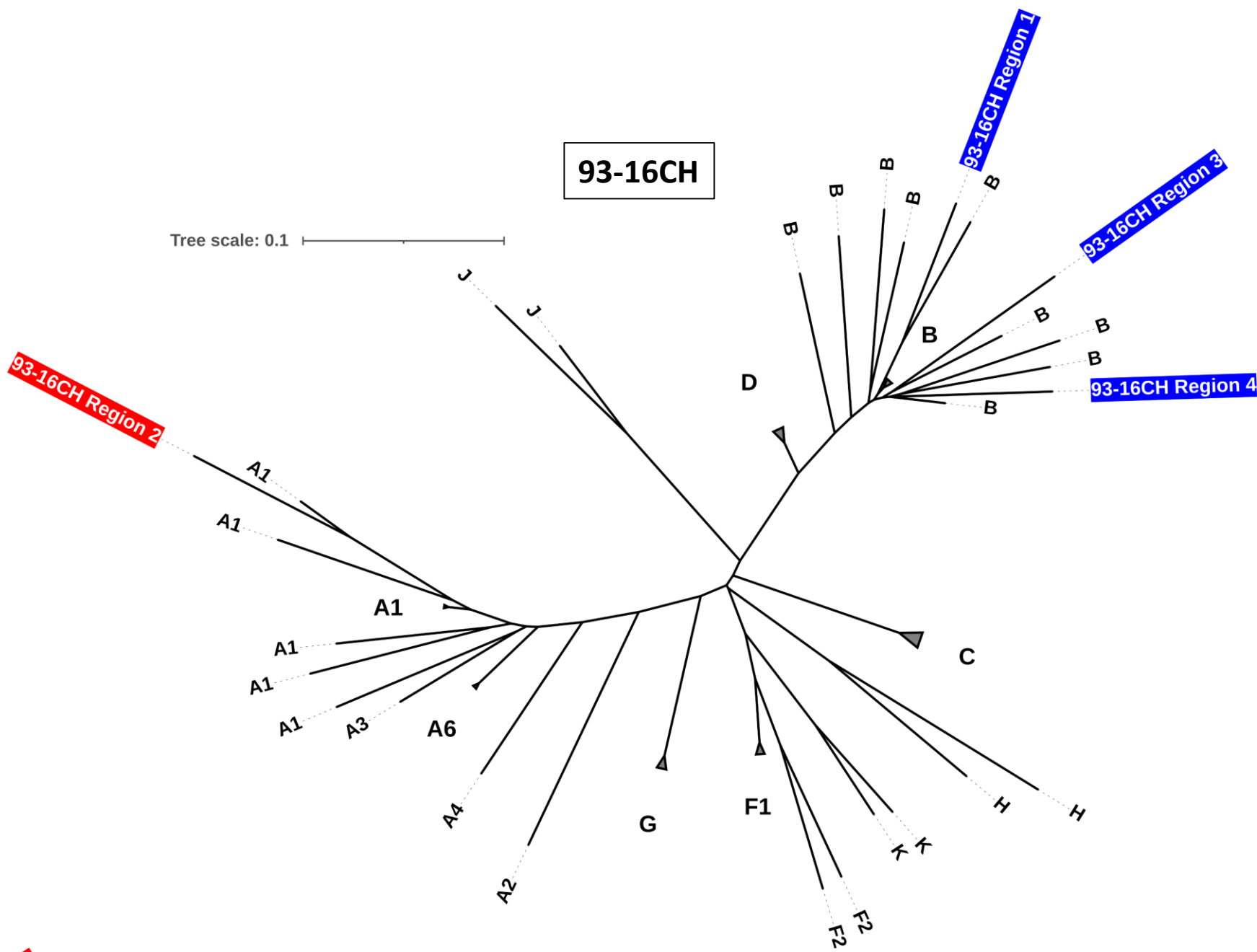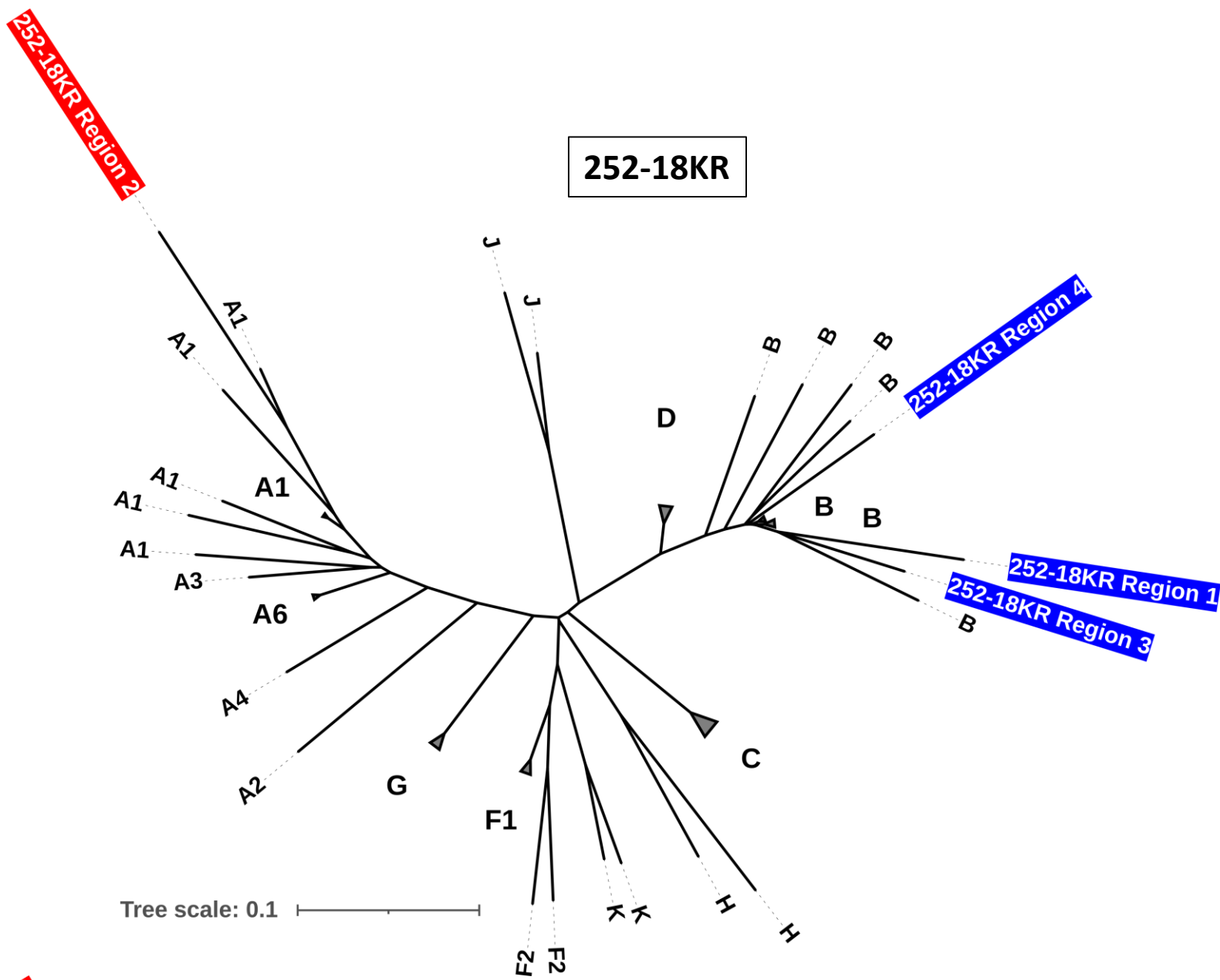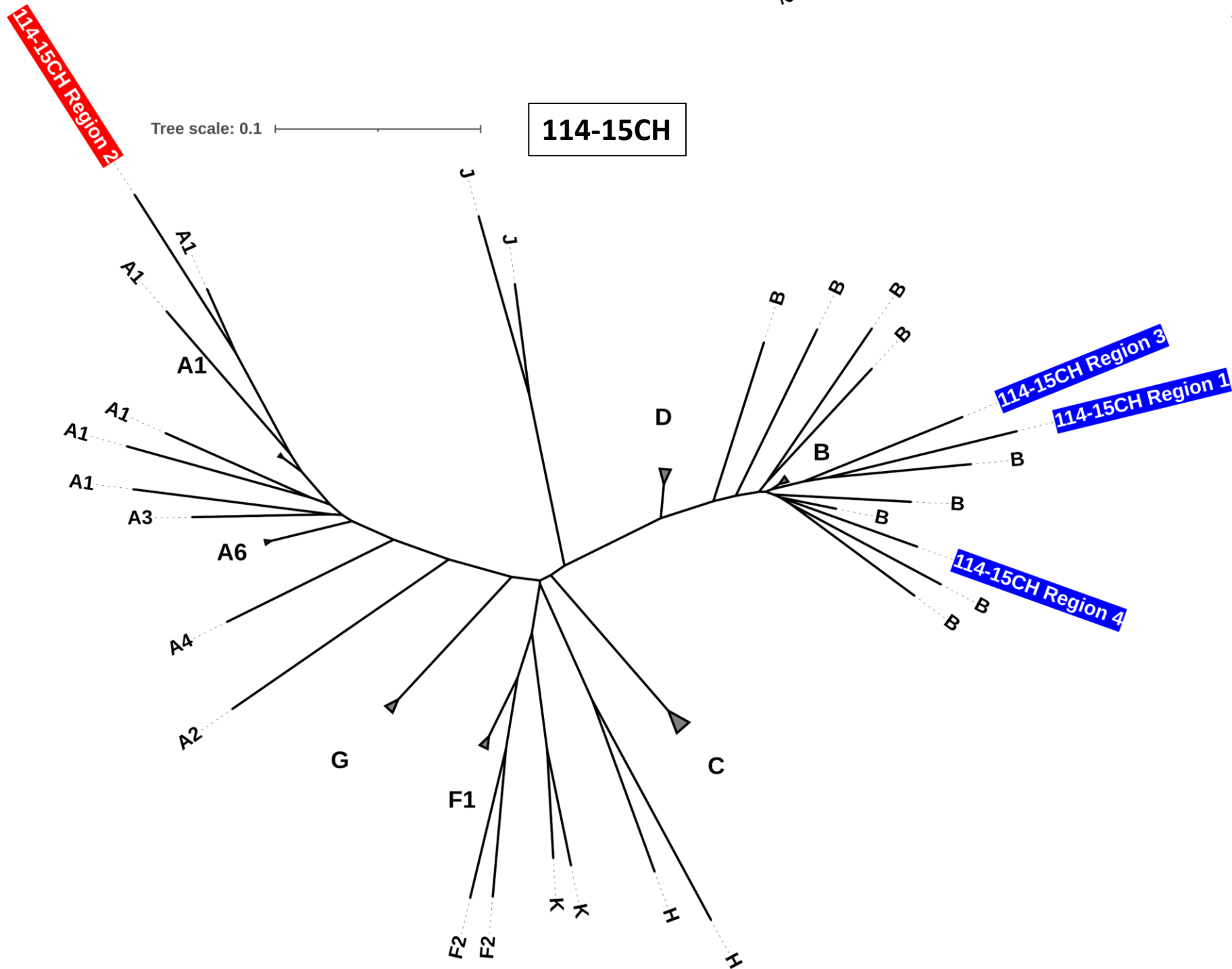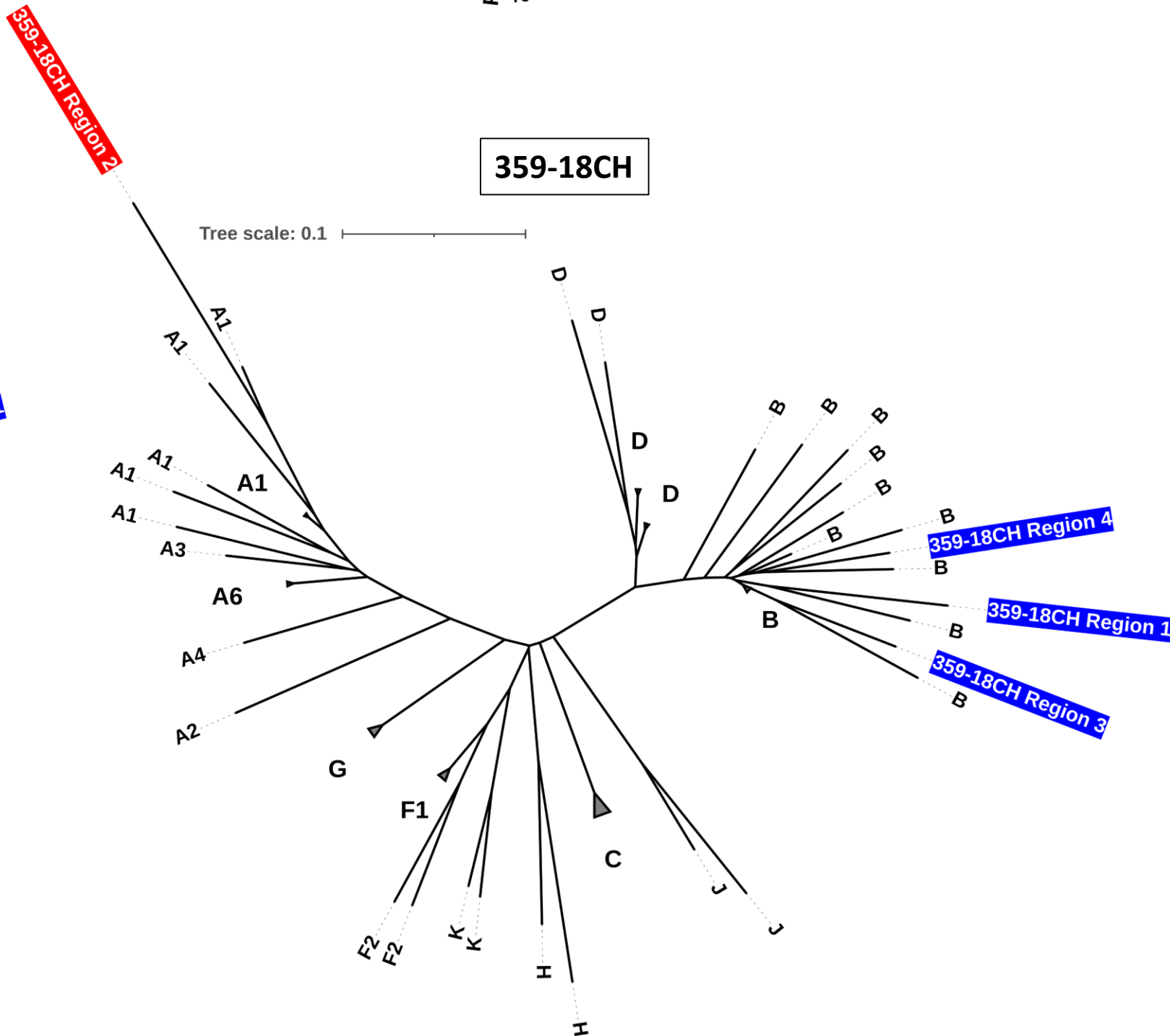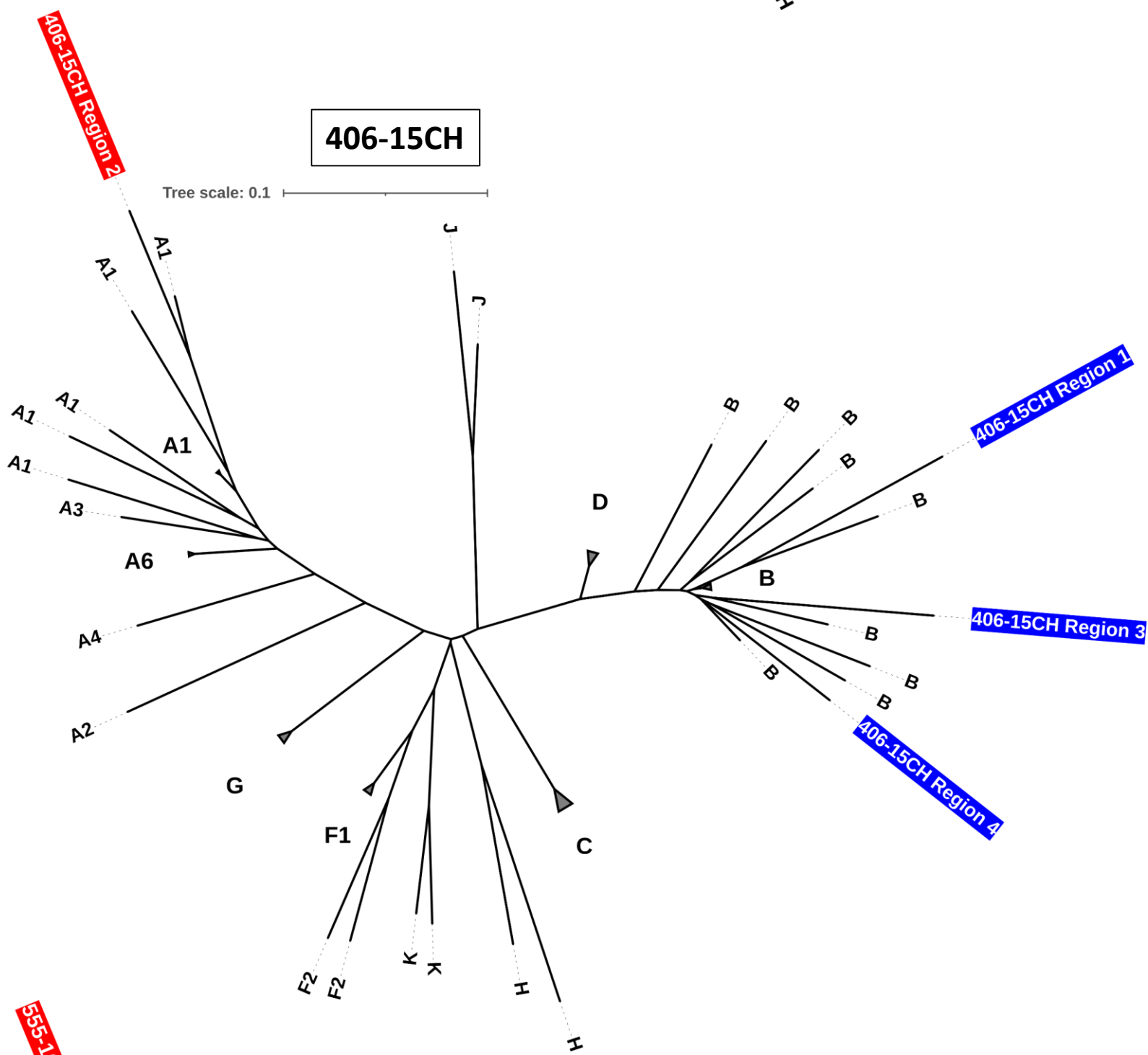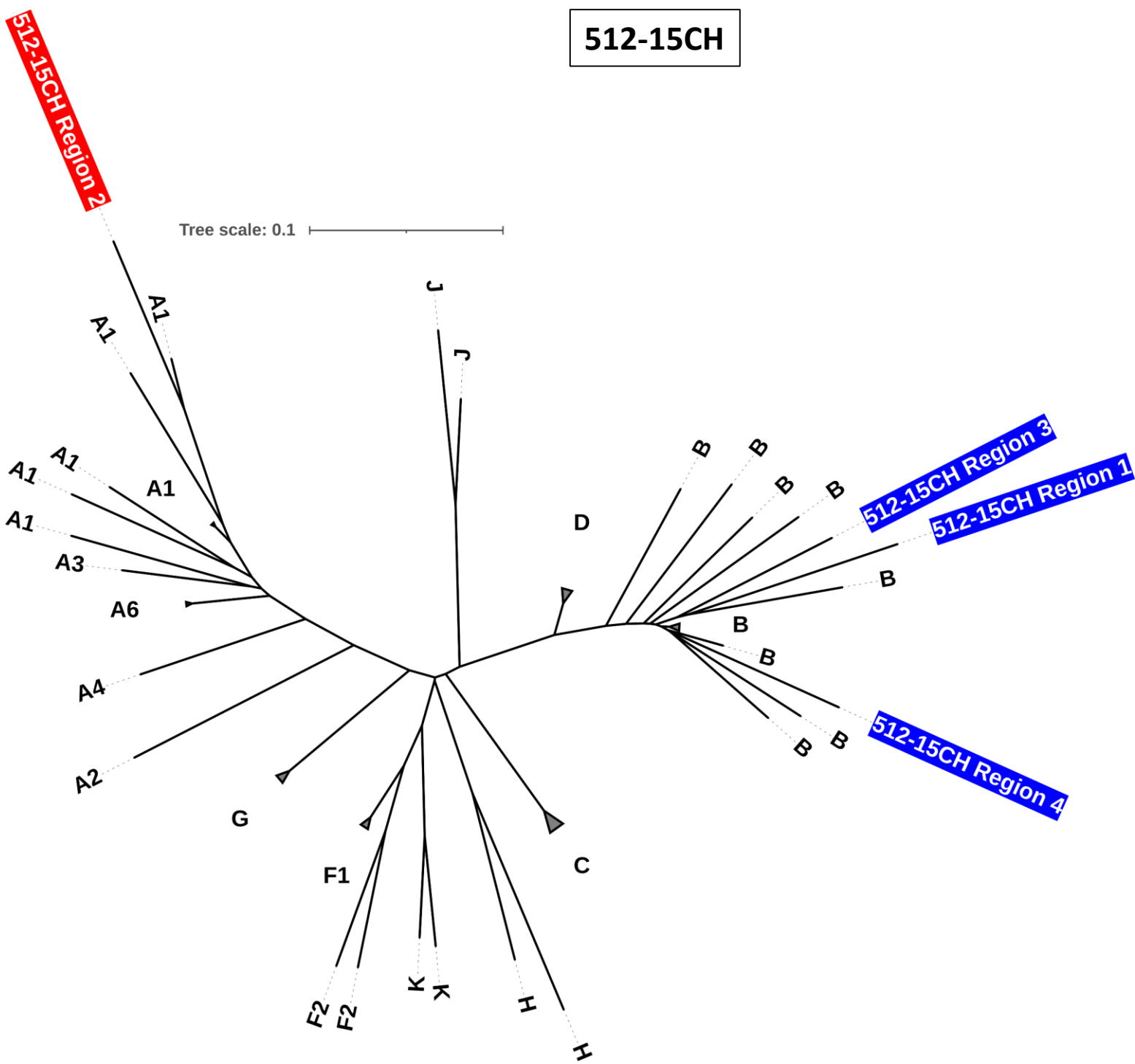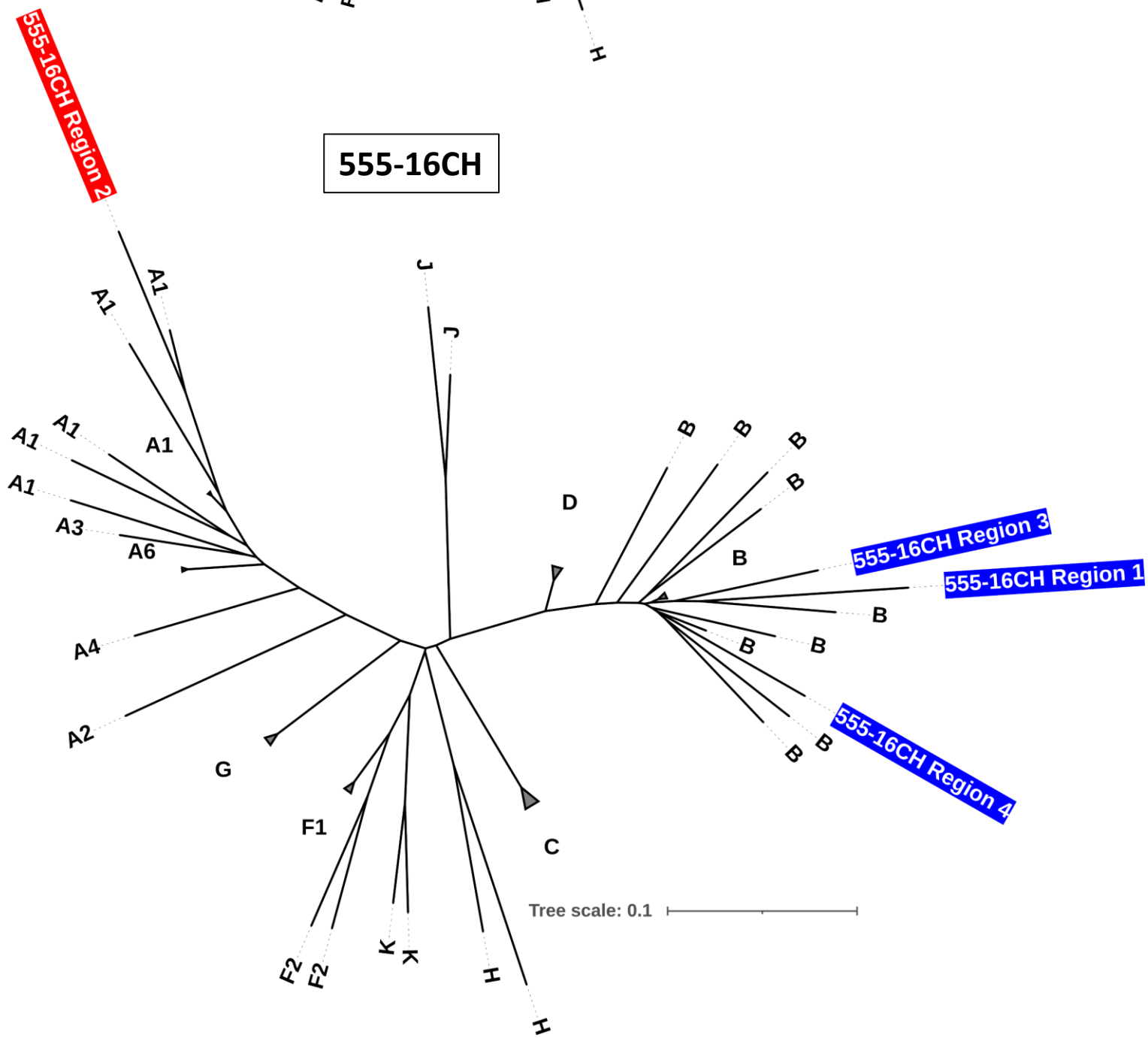

Supplement: Supplementary file 10 — Supplementary Information 10. [file 41598_2021_96125_MOESM10_ESM.pdf]
